# Supplementary material for: No pre-zygotic isolation mechanisms between Schistosoma haematobium and Schistosoma bovis parasites: From mating interactions to differential gene expression
Source: PLoS Negl Trop Dis. 2021 May 4;15(5):e0009363. doi: 10.1371/journal.pntd.0009363 (PMC8127863; doi:10.1371/journal.pntd.0009363)
Supplement: Attachment — Submitted filename: PNTD-D-20-01594_reviewer.pdf [file pntd.0009363.s003.pdf]

PLOS Neglected Tropical Diseases

Pre-zygotic isolation mechanisms between Schistosoma haematobium and Schistosoma bovis parasites: from mating interactions to differential gene expression

--Manuscript Draft--

**Manuscript Number:**	PNTD-D-20-01594
**Full Title:**	Pre-zygotic isolation mechanisms between Schistosoma haematobium and Schistosoma bovis parasites: from mating interactions to differential gene expression
**Short Title:**	Pre-zygotic barriers between schistosome species
**Article Type:**	Research Article
**Keywords:**	Schistosoma; Hybridization; Reproductive isolation mechanisms; RNA-sequencing
**Abstract:**	Species usually develop reproductive isolation mechanisms allowing them to avoid interbreeding. These preventive barriers can act before reproduction “pre-zygotic barriers”, limiting unfavourable mating, or during the life cycle, “post-zygotic barriers”, determining the viability and selective success of the hybrid offspring. Hybridization in parasites and the underlying reproductive isolation mechanisms allowing them to maintain their genetic integrity have poorly been explored and are still misunderstood. Using an integrated approach this work aims to quantify the relative importance of pre-zygotic barriers in Schistosoma haematobium x Schistosoma bovis crosses. These two co-endemic species cause schistosomiasis, one of the major debilitating parasitic diseases worldwide and can hybridize naturally. Using mate choice experiments we first tested if a specific mate recognition system exists between both species. Second, using RNA-sequencing we analysed differential gene expression between homo- and hetero-specific pairing in male and female adult parasites. We show that homo- and hetero-specific pairing occurs randomly between these two species, and few genes in both sexes are affected by hetero-specific pairing. This suggests that i) the choice of mate is not a reproductive isolating factor, and that ii) no pre-zygotic barrier except spatial isolation “by the host” seems to limit interbreeding between these two species. Interestingly, some of the genes affected by the pairing status of the worms can be related to pathways affected during male and female interactions and may also present interesting candidates for species isolating mechanisms and hybridization in schistosome parasites.
**Additional Information:**	
**Question**	**Response**
**Financial Disclosure**<br><br>Enter a financial disclosure statement that describes the sources of funding for the work included in this submission. Review the submission guidelines for detailed requirements. View published research articles from *PLOS NTDs* for specific examples.<br><br>This statement is required for submission and **will appear in the published article** if the submission is accepted. Please make sure it is accurate.	Funding<br>This work has been funded by the French Research National Agency (project HySWARM, grant no. ANR-18-CE35-0001). SM was supported by the Occitania region (project MOLRISK, award no. NREST 2019/1/059), the European “Fonds Européen de Développement Régional” (FEDER) and MRG by the Fellowship of "Estancias breves" (linked to the Programa de Ayudas de Formacion de Profesorado Universitario 2015, Ministerio de Ciencia, Innovación y Universidades, Spain).

Powered by Editorial Manager® and ProduXion Manager® from Aries Systems Corporation

**Unfunded studies**<br><br>Enter: *The author(s) received no specific funding for this work.*<br><br>**Funded studies**<br><br>Enter a statement with the following details:<br>• Initials of the authors who received each award<br>• Grant numbers awarded to each author<br>• The full name of each funder<br>• URL of each funder website<br>• Did the sponsors or funders play any role in the study design, data collection and analysis, decision to publish, or preparation of the manuscript?<br>• **NO** - Include this sentence at the end of your statement: *The funders had no role in study design, data collection and analysis, decision to publish, or preparation of the manuscript.*<br>• **YES** - Specify the role(s) played.<br><br>* typeset	
**Competing Interests**<br><br>Use the instructions below to enter a competing interest statement for this submission. On behalf of all authors, disclose any competing interests that could be perceived to bias this work—acknowledging all financial support and any other relevant financial or non-financial competing interests.<br><br>This statement **will appear in the published article** if the submission is accepted. Please make sure it is accurate. View published research articles from *PLOS NTDs* for specific examples.	The authors have declared that no competing interests exist.

Powered by Editorial Manager® and ProduXion Manager® from Aries Systems Corporation

**NO authors have competing interests**<br><br>Enter: *The authors have declared that no competing interests exist.*<br><br>**Authors with competing interests**<br><br>Enter competing interest details beginning with this statement:<br><br>*I have read the journal's policy and the authors of this manuscript have the following competing interests: [insert competing interests here]*<br><br>* typeset	
**Data Availability**<br><br>Authors are required to make all data underlying the findings described fully available, without restriction, and from the time of publication. PLOS allows rare exceptions to address legal and ethical concerns. See the PLOS Data Policy and FAQ for detailed information.<br><br>A Data Availability Statement describing where the data can be found is required at submission. Your answers to this question constitute the Data Availability Statement and **will be published in the article**, if accepted.	Yes - all data are fully available without restriction

**Important:** Stating ‘data available on request from the author’ is not sufficient. If your data are only available upon request, select ‘No’ for the first question and explain your exceptional situation in the text box.<br><br>Do the authors confirm that all data underlying the findings described in their manuscript are fully available without restriction?	
**Describe where the data may be found in full sentences. If you are copying our sample text, replace any instances of XXX with the appropriate details.**<br><br>• If the data are **held or will be held in a public repository**, include URLs, accession numbers or DOIs. If this information will only be available after acceptance, indicate this by ticking the box below. For example: *All XXX files are available from the XXX database (accession number(s) XXX, XXX.*).<br>• If the data are all contained **within the manuscript and/or Supporting Information files**, enter the following: *All relevant data are within the manuscript and its Supporting Information files.*<br>• If neither of these applies but you are able to provide **details of access elsewhere**, with or without limitations, please do so. For example:<br><br>*Data cannot be shared publicly because of [XXX]. Data are available from the XXX Institutional Data Access / Ethics Committee (contact via XXX) for researchers who meet the criteria for access to confidential data.*<br><br>*The data underlying the results presented in the study are available*	Raw sequences used in this study have been submitted to the Sequence Read Archive under the BioProject PRJNA491632, the transcriptome assembly have been submitted on the Figshare repository (https://figshare.com/s/2f299e6f53c94f4a6168) and they will be released upon publication.

Powered by Editorial Manager® and ProduXion Manager® from Aries Systems Corporation

from (include the name of the third party and contact information or URL).<br>• This text is appropriate if the data are owned by a third party and authors do not have permission to share the data.<br><br>* typeset	
Additional data availability information:	Tick here if the URLs/accession numbers/DOIs will be available only after acceptance of the manuscript for publication so that we can ensure their inclusion before publication.

Powered by Editorial Manager® and ProduXion Manager® from Aries Systems Corporation

Click here to access/download;Manuscript;MS.docx

Pre-zygotic isolation mechanisms between *Schistosoma haematobium* and *Schistosoma bovis* parasites: from mating interactions to differential gene expression

---

Julien Kincaid-Smith[1,2,§], Eglantine Mathieu-Bégné[1§], Cristian Chaparro[1], Marta Reguera-Gomez[3], Stephen Mulero[1], Jean-Francois Allienne[1], Eve Toulza[1], Jérôme Boissier[1]*

[1] IHPE, Univ. Montpellier, CNRS, Ifremer, Univ. Perpignan Via Domitia, Perpignan France.

[2] Centre for Emerging, Endemic and Exotic Diseases (CEEED), Department of Pathobiology and Population Sciences (PPS), Royal Veterinary College, University of London, Hawkshead Campus, Herts, AL9 7TA, UK.

[3] Departamento de Parasitología, Facultad de Farmacia, Universidad de Valencia, Av. Vicent Andrés Estellés s/n, 46100, Burjassot, Valencia, Spain.

* Corresponding author

§: Denotes co-first authors

Short title: Pre-zygotic barriers between schistosome species

Author contributions:

JB and ET conceptualized, supervised the study, and acquired the funding. JKS and SM carried out the experiments and conducted molecular analyses. CC, JFA curated the data. JKS, EMB, MRG performed bioinformatics and statistical analysis. JB, ET, JKS and EMB interpreted the data. JKS and EMB drafted and prepared the manuscript. JB, ET, JKS, EMB and CC revised the manuscript. All authors read and approved the final version of the manuscript.

Availability of data and materials

Raw sequences used in this study have been submitted to the Sequence Read Archive under the BioProject PRJNA491632, the transcriptome assembly have been submitted on the Figshare repository (https://figshare.com/s/2f299e6f53c94f4a6168) and they will be released upon publication.

Funding

This work has been funded by the French Research National Agency (project HySWARM, grant no. ANR-18-CE35-0001). SM was supported by the Occitania region (project MOLRISK, award no. NREST 2019/1/059), the European “Fonds Européen de Développement Régional” (FEDER) and MRG by the Fellowship of "Estancias breves" (linked to the Programa de Ayudas de Formacion de Profesorado Universitario 2015, Ministerio de Ciencia, Innovación y Universidades, Spain).

Competing interest statement

We have no competing interest.

Abstract

Species usually develop reproductive isolation mechanisms allowing them to avoid interbreeding. These preventive barriers can act before reproduction “pre-zygotic barriers”, limiting unfavourable mating, or during the life cycle, “post-zygotic barriers”, determining the viability and selective success of the hybrid offspring. Hybridization in parasites and the underlying reproductive isolation mechanisms allowing them to maintain their genetic integrity have poorly been explored and are still misunderstood. Using an integrated approach this work aims to quantify the relative importance of pre-zygotic barriers in *Schistosoma haematobium x Schistosoma bovis* crosses. These two co-endemic species cause schistosomiasis, one of the major debilitating parasitic diseases worldwide and can hybridize naturally. Using mate choice experiments we first tested if a specific mate recognition system exists between both species. Second, using RNA-sequencing we analysed differential gene expression between homo- and hetero-specific pairing in male and female adult parasites. We show that homo- and hetero-specific pairing occurs randomly between these two species, and few genes in both sexes are affected by hetero-specific pairing. This suggests that i) the choice of mate is not a reproductive isolating factor, and that ii) no pre-zygotic barrier except spatial isolation “by the host” seems to limit interbreeding between these two species. Interestingly, some of the genes affected by the pairing status of the worms can be related to pathways affected during male and female interactions and may also present interesting candidates for species isolating mechanisms and hybridization in schistosome parasites.

Keywords: Schistosoma, Hybridization, Reproductive isolation mechanisms, RNA-sequencing

Author summary

Understanding how species maintain their genetic integrity is a central question in evolutionary science. If isolation mechanisms are well documented in free-living organisms, it is currently not the case for parasite species. Yet, occurrence of parasite hybrids is a critical global health concern since these hybrids are expected to be more harmful than parental species. We addressed the question of reproductive isolation mechanisms in parasitic species by conducting an integrative experimental study (from mate choice to gene expression) on two schistosome species (*S. haemtobium* and *S. bovis*) that parasite human and cattle respectively and for which hybrids have been responsible of recent outbreaks, including out of endemic areas. We showed that *S. haematobium* and *S. bovis* mate randomly rather than have preferred homo-specific mate choice, but also that they only express a few genes differentially when involved in a hetero-specific pair compared to a homo-specific pair. We consequently suggest that these two schistosome species lack strong isolation mechanism but the one imposed by host specificity. Our results raise the concern that in absence of post-zygotic barriers, hybridization between both species might be more common than previously thought in areas where the two species overlap and encounter.

Introduction

Usually species cannot interbreed. A subset of obstacles has evolved in the course of speciation in order to limit gene flow via hybridization and maintain species boundaries. These obstacles are traditionally classified as pre- and post-zygotic barriers (also known as pre- or post-mating barriers) and can be defined as any mechanisms preventing or reducing gene flow between groups of potentially interbreeding individuals [1]. Pre-zygotic barriers include spatial isolation (two species live in different habitats), behavioural isolation (individuals can choose to mate with individual of their own species), temporal isolation (copulation does not occur at the same time *e.g.*, different seasons) mechanical isolation (sex organs are not compatible) and gametic isolation (the gametes encounters but fertilization does not occur). When the first barrier is crossed, post-zygotic isolation mechanisms can arise to prevent gene flow. Post-zygotic barriers include hybrid unviability (hybrids die prematurely), reduced fitness with low fertility (hybrids are poorly fertile or infertile) or hybrid breakdown (a longer process where the hybrids lines are counter-selected compared to their parental forms). The strength and/or the order of each barrier are variable among species making difficult to predict the evolution of reproductive isolation mechanisms [2]. Moreover, reproductive isolation is often the result of an accumulation and interaction of multiple pre- and post-zygotic mechanisms restricting most gene flow [3]. However, it is generally admitted that the pre-zygotic isolation barriers are enhanced in sympatric species [4] and are the most effective because they act early to prevent producing un-adapted progeny [5].

Despite their importance in term of biodiversity [6] and animal or human health, parasite species have received less attention than free living organisms both in terms of hybridization and understanding the role of pre- or post-zygotic mechanisms in their reproductive isolation [7]. The pre-zygotic barriers usually include additional and stronger obstacles to overcome compared to free living organisms. For instance, the "habitat barrier" includes both the

geographic area, the host species and tropism within the host. For parasites, hosts are inimical habitats imposing strong selective pressures (co-evolutionary arms race) requiring constant adaptation of parasites for the completion of their life cycle. The specialisation of parasite species to a particular host is thus a strong pre-zygotic isolation mechanism shaped through its hosts potentially preventing hybridization and favouring speciation. However, some related species manage to retain their genetic identity while parasitizing the same host, meaning that they have acquired selective mechanisms for reproductive isolation. Hybridization and pre-zygotic reproductive barriers have been studied on very few parasite models: plasmodium, cestodes and schistosomes [7–9]. Partial pre-zygotic barriers have been evidenced between *Plasmodium berghei* and *P. yoeli* [8]. It was not the case between *Schistocephalus solidus* and *S. pungitii* [7], suggesting in this last case that post-zygotic selection against hybrids is presumably the most important driving force limiting gene flow between these two parasite sister species [7].

Schistosomes are among the world greatest concerns in terms of human and animal health because they cause schistosomiasis debilitating diseases affecting over 230 million of people in the world [10]. There are currently six *Schistosoma* species infecting humans and 19 species infecting animals [9]. These parasites have a two-host life cycle, which includes a mammalian definitive host in which sexual reproduction occurs and a mollusc intermediate host in which asexual multiplication takes place. Schistosomes have the particularity of having separate sexes among other trematodes [11,12] and have therefore been intensively studied for their sexual features including male-female interactions [13,14], sex-ratio [15,16], mating system [17,18] and mating behaviour [19]. One direct consequence of dioecism in these species is the necessity of individuals of both sexes to infect the same definitive host. This constraint can lead to interaction of species infecting the same host, and in the case of porous reproductive pre-zygotic barriers to hybridization.

To conserve their genetic identity, schistosomes that inhabit the same definitive host are expected to present pre-zygotic isolating mechanisms. Among these barriers, habitat and behavioural isolation have a great interest in schistosome's sexual interactions. First habitat isolation is a three-level constraint that initially has to be overcome: the geographic area, same host and same localisation in the host. Indeed, schistosomes species are distributed worldwide (the majority in Africa), the vertebrate host specificity depends on the parasite species, and while the majority of species live in the mesenteric vein system, one species (*S. haematobium*) lives in the veins surrounding the bladder of humans. Second, behavioural isolation is more complex in schistosomes than in other species because mate choice is followed by a pairing-dependent differentiation of the female sexual organs [13,14]. Studies have clearly established that the presence of the male is necessary not only for the female sexual development but also for the maintenance of sexually mature and active state and does not depend upon species-specific pairing [20–22]. It was also demonstrated that female schistosomes stimulate the males through changes in level of glutathione and lipids, and stimulates tyrosine uptake in the male worms [13]. Hence, while males transfer glucose and lipid secretions to females, females also release factors affecting male worms physiology [23–26]. Thus, male and female schistosomes are strongly co-dependent, in terms of behaviour (*i.e.*, they have complementary roles in the hosts) but also physiologically [11] and an intimate and permanent association between sexes is necessary for reproduction to occur.

Nevertheless, several hybrid schistosomes have been evidenced and like in several other clades the isolation mechanisms increase with divergence time between taxa [4,9]. Hence, studies on Schistosome mating choices have revealed that depending on the parasite species some combinations readily pair (*S. haematobium x S. intercalatum* and *S. bovis x S. curassoni*, *S. mansoni x S haematobium*), whereas others may be more selective and present mate choice systems (*S. mansoni x S. intercalatum*, *S. haematobium* x *S. mattheii*, and *S. mansoni x S.*

margrebowiei crosses [27–30]). In the former case and for many related species capable of hybridizing host specificity hence may be the sole barrier preventing interbreeding. This isolation mechanism “by the host” may be so efficient that species may lack any post-zygotic or other pre-zygotic mechanisms ultimately allowing them to hybridize when the opportunity arises. Therefore, the lack of reproductive incompatibility (*i.e.*, isolation by behaviour and physiology) between schistosome species infecting humans and animals may facilitate gene flow if the host isolating barriers are broken down.

Schistosoma haematobium x S. bovis hybrids are today the most studied hybrid system of schistosomes. These hybrids have first been identified in Niger by Brémont [31] and more recently in Senegal [32] but seem widely distributed in West Africa [31–36]. Moreover, this hybrid cross has recently been responsible for a large-scale outbreak in Europe (Corsica, France), where transmission of the disease is persistent [34,37]. *Schistosoma haematobium* and *S. bovis* are co-endemic in Africa, but their tropism is different (veins surrounding the bladder for *S. haematobium vs.* mesenteric veins *S. bovis*) and their definitive hosts are also different. *S. haematobium* is mainly a parasite of human. However, sporadic studies have shown that non-Human primates, *Cetartiodactyla* or rodents could be naturally infected by this parasite species [38–40]. Conversely, *S. bovis* is mainly a parasite of ruminant with sporadic cases of rodent infection [38]. Interestingly recent studies showed that *S. haematobium* x *S. bovis* hybrids naturally infect rodent or cattle [36,41]. Hybridization between these two species is particularly worrying because it raises the eventuality for a human parasite to become zoonotic but also because it may lead to changes in the parasites life history traits including their response to chemotherapeutic treatment [42]. Also these hybrids often display heterosis, in which their fitness outperforms parental species [9,32,33]. Importantly the existence of a mate recognition system between the two species would prevent natural occurrences of hybridization in

sympatric areas. At the contrary a lack of reproductive isolation could indicate that occurrences of hybridization may be more frequent.

This study hence proposes an integrated approach, from mating behaviour to male and female gene expression, in order to quantify the relative importance of pre-zygotic barriers in *S. haematobium x S. bovis* crosses. First, using a mate choice experiment we tested whether specific mate recognition exists by quantifying the frequency of hetero-specific and homo-specific pairs compared to random mate choice expectations. Second, given the strong co-dependence between male and female schistosomes, we also analysed the influence of pairing (homo- vs hetero-specific) on the transcriptomic profile of male and female parasites using RNA sequencing. The molecular determinants of the very first step towards hybridization may give further insight into the permeability of the two species and reveal some important genes linked to male and female interaction, species isolation and hybridization. Relying on such an integrative approach from mate choice to gene expression hence allowed us to provide a better understanding of the mechanisms preventing and allowing hybridization between *S. haematobium* and *S. bovis.*

Materiel and methods

Ethics Statement

Experiments were carried out according to national ethical standards established in the writ of February 1st, 2013 (NOR: AGRG1238753A), setting the conditions for approval, planning and operation of establishments, breeders and suppliers of animals used for scientific purposes and controls. The French Ministry of Agriculture and Fishery (Ministère de l’Agriculture et de la Pêche), and the French Ministry for Higher Education, Research and Technology (Ministère de l’Education Nationale de la Recherche et de la Technologie) approved the experiments carried

out for this study and provided permit A66040 for animal experimentation. The investigator possesses the official certificate for animal experimentation delivered by both ministries (Décret n° 87–848 du 19 octobre 1987; number of the authorization 007083).

Parasite species and experimental infections

The experimental design of this work was set for two objectives, i) assess the species mating choices by quantifying the frequency of hybridization, ii) force reciprocal hybridization and assess the transcriptomic changes occurring between homo-specific and hetero-specific paired males and females.

Schistosoma haematobium and *S. bovis* were maintained in the laboratory on *Bulinus truncatus* snails as intermediate hosts and *Mesocricetus auratus* as definitive hosts. The species strains used here originate from Cameroon and Spain for *S. haematobium* and *S. bovis*, respectively [63]. Experimental infections of hamsters with mixed combinations of both species in controlled proportions were performed after sexing procedure of the cercariae emitted by individually infected molluscs [63]. Briefly, *Bulinus truncatus* molluscs were individually exposed to one *miracidium* of *S. haematobium* or *S. bovis.* After a minimum of 55 days after exposing the molluscs to the parasites, successfully infected molluscs released either male or female clonal populations of c*ercariae* of each species. After collecting *cercariae* from each individual mollusc, molecular sexing of the parasites where performed as described previously in Kincaid-Smith et al. (2016). These molluscs were then classified as releasing male or female *cercariae* of a specific species (*S. haematobium* or *S. bovis*) and were used for the subsequent experiments. A schematic view of the experimental infection procedure is presented in Fig 1. Depending on each objective, hamsters were subsequently infected with specific mixed combinations of *cercariae* by surface application method for one hour. Detailed methods employed for molluscs and rodents infections were described previously [64–66] and the

number and combinations of the *cercariae* used for each infestation is presented in Table 1. Specifically, in order to test species mate choices five hamsters were infected with mixed combinations of *cercariae* species and relied on the experiment 1 to 5 (*i.e.*, Exp. 1 to Exp. 5, see Table 1). Experiments 1 to 4 represent a limited choice of mate (Exp. 1 and Exp. 3 male choice - Exp. 2 and Exp. 4 female choice). In these experiments the excess of one sex ensures that all individuals of the other sex will be mated (Table 1). Experiment 5 represents species full choice of mate and is performed using the same number of *cercariae* of both sexes and both species (Table 1). According to our second objective, homo- and hetero-specific control experiments presented in Table 1 (*i.e.*, Homo control 1, Homo control 2, Hetero control 1 and Hetero control 2) are simultaneous infections without mate choice and are used to infer the transcriptomic profiles of homo- and hetero-specific paired worms (Table 1). Finally, for all infestations, three months after exposing hamsters to *cercariae*, the number of single and homo-specific versus hetero-specific paired worms was assessed after recovering worms by portal perfusion of the hamsters [66], separating couples and individualizing each partner in 96 well plates. Each worm and their status (paired, or single) as well as their sex (male or female) was thus carefully assigned.

Fig 1. **Schematic representation of experimental infection procedure.**

Table 1: **Experimental procedure to evaluate mate choice of *S. haematobium and S. bovis* parasites.** For each objective are listed the experiments conducted as well as the number of males and females *S. haematobium* and *S. bovis* used and the number of biological replicates (number of hamsters).

**Experiments**	***S. haematobium***		***S. bovis***		**Biological replicates**
	**Males**	**Females**	**Males**	**Females**	
**Objective 1:** Assess species mate choice and hybridisation frequency					
**Limited Choice**					
Exp. 1 (*S. haematobium* males' choice)	150	225	-	225	5
Exp. 2 (*S. haematobium* females' choice)	225	150	225	-	5
Exp. 3 (*S. bovis* males' choice)	-	225	150	225	5
Exp. 4 (*S. bovis* females' choice)	225	-	225	150	5
**Full Choice**					
Exp. 5	150	150	150	150	5
**Objective 2:** Assess the transcriptomic profiles of homo- and hetero-specific paired worms					
Homo-specific pairing control 1	300	300	-	-	6
Homo-specific pairing control 2	-	-	300	300	6
Hetero-specific pairing control 1	300	-	-	300	6
Hetero-specific pairing control 2	-	300	300	-	6

DNA extraction and species diagnostic multiplex PCR

The worms collected were subjected to DNA extraction was performed as described previously [67]. To identify the species of each worm we used a rapid diagnostic procedure based on multiplex PCR reaction enabling the distinction between *S. haematobium* and *S. bovis* [68,69]. Briefly, the reaction is composed of a universal forward primer and two specific reverse primers targeting either *S. haematobium* or *S. bovis* COI gene. In presence of *S. haematobium* DNA the amplicon size is of 543 bp whereas in the presence of *S. bovis* DNA the amplicon size is of 306 bp allowing discriminating *S. haematobium* from *S. bovis* parasites (Table 2).

Table 2: PCR primer sequences and amplicon size [68,69].

Target species primer	Sequence (3'-5')	Amplicon size
Universal forward primer	TTTTTTGGTCATCCTGAGGTGTAT	
S. haematobium reverse primer	TGATAATCAATGACCCTGCAATAA	543bp
S. bovis reverse primer	CACAGGATCAGACAAACGAGTACC	306 bp

The PCR reactions were carried out in a total volume of 12.5 µl containing GoTaq Flexi Reaction Buffer (Promega), 1.5 mM of $MgCl_2$, 0.4 µM of each of the 3 primers (at 10 µM each), 0.2 µM of dNTP solution (at 10 µM each), 0.5 U of GoTaq G2 Hot Start Polymerase (Promega, USA) and 1 µl of diluted DNA (1/10). The PCR program was: an initial denaturation phase at 95°C for 3 min, followed by 35 cycles at 94°C for 30s, 58°C for 1.30s, 72°C for 1.30s, and a final extension at 72°C for 7 min. PCR products were examined on 1.5% agarose gels at 120V for 45 min and using a 100 bp DNA-Ladder (Promega) for size estimation.

Statistical analysis

After counting the number of homo-specific pairs, hetero-specific pairs and single worms of each species, we calculated the expected number of single and mated pairs according to the null hypothesis of random pairing (*e.g.*, in the Exp. 1, the expected number of homo-specifically paired *S. haematobium* males equals the total number of *S. haematobium* males times the total number of *S. haematobium* females over the total number of females). Expected and observed numbers of homo- and hetero-specific pairs were then compared using Chi-square tests.

RNA extraction and transcriptome sequencing of homo- and hetero-specific S. haematobium and S. bovis male and female pairs

After portal perfusion of the hamsters containing the homo-specific and hetero-specific paired worms (homo-specific pairing control 1 and 2, and hetero-specific pairing control 1 and 2), paired adult worms were isolated to constitute separate triplicates of males and females homo-specifically paired, and triplicates of males and females hetero-specifically paired. Pools of 10-12 worms were gathered in 2ml microtubes and immediately frozen in liquid nitrogen before they were stored at -80°C. TRIzol^{tm} RNA extraction and subsequent paired-end Illumina HiSeq 4000 PE100 sequencing technology was performed on the samples. Briefly, pools of adult worms were subsequently grounded with two steel balls using Retsch MM400 cryobrush (2 pulses at 300Hz for 15s). Total RNA was extracted using the TRIzol^{tm} Thermo Fisher Scientific protocol (ref: 15596018) slightly modified as each reagent volumes were halved. Total RNA was eluted in 44 µl of ultrapure water before undergoing a DNase treatment using Thermofisher Scientific Turbo DNA-free kit. RNA was then purified on a column using the Qiagen RNeasy mini kit and eluted in 42µl of ultrapure water. Quality and concentration were assessed by spectrophotometry with the Agilent 2100 Bioanalyzer system and using the Agilent RNA 6000 nano kit. Further details are available at Environmental and Evolutionary Epigenetics Webpage (http://methdb.univ-perp.fr/epievo/).

Illumina libraries construction and high-throughput sequencing

cDNA library construction and sequencing were performed at the Génome Québec platform. The TruSeq stranded mRNA library construction kit (Illumina Inc., USA) was used following the manufacturer's protocol on 300 ng of total RNA per sample. Sequencing of the 24 samples was performed in 2x100 bp paired-end on a Illumina HiSeq 4000. Sequencing data are available at the NCBI-SRA under the BioProject PRJNA491632.

Transcriptomic analysis of hetero-specific pairing vs. homo-specific pairing effect

Raw sequencing reads were analysed on the Galaxy instance of the IHPE laboratory [70,71] implemented on the local server of our laboratory. First, raw reads were subjected to quality assessment and sequencing adaptor trimming. For this we used the set of tools based on FASTX-toolkit [72], as well as Cutadapt program (Galaxy Version 1.16.1) to remove adapter sequences from Fastq files [73]. At last each sample reads were associated into pairs using the FASTQ interlacer/de-interlace programs (Galaxy Version 1.1). Processed reads were mapped using RNA-star Galaxy Version 2.6.0b-1 [74] to the *S. haematobium* reference genome [43] downloaded from the Schistosoma Genomic Resources website SchistoDB (http://schistodb.net/common/downloads/Current_Release/ShaematobiumEgypt/fasta/data/). Exon-intron structure was thereafter reconstructed for each mapping BAM file using Cufflinks transcript assembly Galaxy Version 2.2.1.2, by setting the max intron length at 50000, but without any correction parameters [75]. Finally, in order to create a reference transcriptome representative of *S. haematobium* and *S. bovis* male and female reads, we merged all cufflinks data with Cuffmerge Galaxy Version 2.2.1.2 [75] without using any guide or reference. This enabled us to create a representative reference transcriptome of both species and both sexes using the same reference genome. The Genomic DNA intervals of each newly assembled genes of this reference transcriptome was extracted from *S. haematobium* reference genome and converted into a Fasta file.

The number of reads per transcripts for each sample (*i.e.*, the read abundance representative of each gene) was quantified using HTseq-count Galaxy Version 0.9.1 on the reference transcriptome, setting the overlap resolution mode on “union” [76]. Finally, we evaluated the differential gene expression levels between homo-specific and hetero-specific worms for each species and each sex separately using DESeq2 Version 1.28.1 [77] run on R version 4.0.0 [78].

We carried out four types of comparisons which respectively focused on *S. haematobium* males, *S. haematobium* females, *S. bovis* males and *S. bovis* females and contrasted genes expression profiles between hetero-specifically paired individuals and homo-specifically paired individuals. Differential gene expression results were filtered on the adjusted *P*-value (Benjamini-Hochberg multiple testing based False Discovery Rate (FDR)) and considered significant when ≤ 5%.

Functional annotation

Using our BLAST local server, we annotated entirely the *de novo* assembled transcriptome by Blastx search against the non-redundant database of the NCBI. We conserved only the longest unique transcript (TCONS) of each representative gene (XLOC) for Blastx search and subsequent analysis. Output XML files were used for gene ontologies (GO) mapping and annotation using Blast2Go version 4.1.9 [44]. Finally, enrichment Fisher's exact tests were performed on up and down regulated set of genes focusing on biological process (BP) ontology terms. The *P-value* for significance was set to 5% False Discovery Rate (FDR).

Results

Mating choice experiments

Limited choice: Experiments 1 to 4:

Details on the number of worms recovered from each hamster and whether they were paired or single in limited choice experiments are summarized in Table 3. Regardless of the sex and the species of the worms choosing to pair with either an homo-specific or an hetero-specific partner (*i.e.*, *S. haematobium* males, *S. haematobium* females, *S. bovis* males and *S. bovis* females, in Exp. 1, 2, 3 and 4 respectively, Table 3) both homo-specific and hetero-specific pairs were observed (Table 3, Exp. 1–4). Also, in each limited choice experiments (Exp. 1–4) we

consistently obtained an excess of both homo-specific and hetero-specific potential partners that remained single whereas all choosing partners (the one being the focus of each experiment) were paired (Table 3). This indicates that the choosing partner in each experiment was not limited in its choice by the number of homo- nor hetero-specific partner.

Specifically, regarding *S. haematobium* males' choice (Table 3, Exp. 1) the number of homo- and hetero-specific pairs was significantly different from those expected under the random mating hypothesis ($\chi2$=11.10; d.f.=4; p-value= 0.049, Table 3). This was due in particular, to a bias toward hetero-specific pairs in only one hamster (hamster number 3, see Table 3). Regarding *S. haematobium* females' choice (Table 3, Exp. 2) at the contrary, the numbers of homo-specific pairs and hetero-specific pairs were not significantly different from expectations under the random mating hypothesis ($\chi2$=3.118; d.f.=4; p-value=0.682, Table 3). In the experiment 3 that focused on *S. bovis* males' choice, the number of paired worms recovered was extremely low, due to premature death of two hamsters and only two hamsters had enough worms to be analysed, although in this case statistics should be interpreted with caution (Table 3, Exp. 3). Nevertheless, the numbers of homo-specific pairs and hetero-specific pairs were once again not significantly different from expectations under the random mating hypothesis ($\chi2$=4.522; d.f.=1; p-value=0.104, Table 3). Finally, regarding *S. bovis* females' choice (Table 3, Exp. 4) similarly we did not found a significant difference between the numbers of observed and expected homo-specific pairs and hetero-specific pairs under random mating hypothesis ($\chi2$=3.246; d.f.=4; p-value=0.662, Table 3). Overall, when analysing all limited choice experiments together (*i.e.*, Exp. 1 to 4) no significant difference was recorded between the number of observed homo- and hetero-specific pairs and the number of homo- and hetero-specific pairs expected in a random mating scenario ($\chi2$=21.71, d.f.=16, p-value=0.152, Table 3).

Table 3. Summarized information of experiments one to four (limited choice). For each experiment are displayed the sex and the species of the choosing partner, the number of observed homo- and hetero-specific pairs and the number of (non-choosing) partners remained single. Sh stands for *S. haematobium* and Sb stands for *S. bovis*. Expected number of pairs regarding a random mating and initial partners frequencies are given into brackets. Chi-square statistic, degree of freedom and p-value are given for each hamster, for each experiment and for all experiment combined. * indicates significant results.

**Exp.**	**Host**	**Choosing partner**	**Homo-sp. pairs**	**Hetero-sp. pairs**	**Homo-sp. singles**	**Hetero-sp. single**	**χ2-statistic**	**d.f.**	**p-value**
1	1	♂ Sh	11 (14)	14 (11)	20	9	1.838	1	0.175
1	2	♂ Sh	11 (15)	22 (18)	15	11	1.543	1	0.214
1	3	♂ Sh	14 (20)	16 (10)	25	4	5.057	1	0.025*
1	4	♂ Sh	9 (9)	6 (6)	36	26	0.015	1	0.903
1	5	♂ Sh	10 (13)	10 (7)	41	15	2.651	1	0.103
**Total Exp. 1**							**11.104**	**4**	**0.049***
2	1	♀ Sh	10 (9)	2 (4)	7	5	0.908	1	0.341
2	2	♀ Sh	6 (5)	1 (2)	0	1	0.429	1	0.513
2	3	♀ Sh	12 (10)	3 (5)	11	10	1.688	1	0.194
2	4	♀ Sh	16 (15)	3 (4)	6	2	0.094	1	0.759
2	5	♀ Sh	12 (12)	13 (13)	11	12	<0.001	1	0.993
**Total Exp. 2**							**3.118**	**4**	**0.682**
3	2	♂ Sb	4 (2)	0 (2)	46	55	4.400	1	0.036
3	3	♂ Sb	2 (1)	1 (2)	22	32	0.742	1	0.389
**Total Exp. 3**							**4.522**	**1**	**0.104**
4	1	♀ Sb	15 (12)	17 (20)	4	14	1.070	1	0.301
4	2	♀ Sb	10 (8)	25 (28)	2	19	1.061	1	0.303
4	3	♀ Sb	3 (3)	8 (8)	4	13	0.030	1	0.862
4	4	♀ Sb	9 (8)	15 (16)	8	19	0.188	1	0.665
4	5	♀ Sb	49 (49)	15 (15)	18	5	0.007	1	0.932
**Total Exp. 4**							**3.246**	**4**	**0.662**
**Total all Exp.**							**21.719**	**16**	**0.152**

Full choice: Experiment 5

Details on the number of worms recovered from each hamster and whether they were paired or single in limited full choice experiments are summarized in Table 4. When all partner choices were allowed between males and females *S. haematobium* and *S. bovis*, four combinations of pairing were obtained: two being homo-specific (♂ Sh x ♀ Sh and ♂ Sb x ♀ Sb, Table 4) and two being hetero-specific (♂ Sh x ♀ Sb and ♂ Sb x ♀ Sh, Table 4). There was also an excess of males and females of both species remaining single, suggesting that all possible choices were not limited by partner availability (Table 4). Regarding the number of homo-specific and hetero-specific pairs observed between *S. haematobium* and *S. bovis*, Chi-square tests did not reveal significant departure from random mating hypothesis, when the number of each combination of pairing was analysed in each hamster separately and also when analysing all replicate together (Table 4).

Table 4. Summarized information of experiment 5 (full choice). For each combination (*i.e.*, sex and species) are given the number of observed pairs and the number of single partners remained single. Sh stands for *S. haematobium* and Sb stands for *S. bovis*. Expected number of pairs from a random mating and given the initial partners frequencies are indicated into brackets. Chi squared statistics, degree of freedom and p-value are given per hamster and for the whole experiment.

Host no.	***♂Sh* x ♀ Sh**	**♂Sb x ♀ Sb**	**♂Sh x ♀ Sb**	**♂Sb x ♀ Sh**	**♂ Sh**	**♂ Sb**	**♀ Sh**	**♀ Sb**	**χ2-statistic**	**d.f.**	**p-value**
1	1 (2)	29 (24)	5 (9)	4 (5)	8	5	4	9	3.358	3	0.340
2	8 (8)	2 (1)	5 (4)	1 (2)	8	3	23	8	1.786	3	0.618
3	7 (5)	2 (2)	1 (2)	2 (3)	6	5	19	11	2.307	3	0.511
4	4 (4)	6 (3)	3 (4)	1 (3)	10	4	13	11	4.806	3	0.187
5	5 (6)	1 (1)	6 (6)	2 (1)	20	3	17	16	0.796	3	0.850
**Total**									**13.053**	**12**	**0.365**

Transcriptomic response in homo- vs. hetero-specific pairs

RNA sequencing, transcriptome assembly and gene annotation of the homo- and hetero-specific pairs

Each condition (*i.e.*, homo-specifically paired *S. haematobium*, hetero-specifically paired *S. haematobium*, homo-specifically paired *S. bovis*, and hetero-specifically paired *S. bovis*) composed of three biological replicates were analysed separately for males and females (24 samples). Between ~24.7 and ~42.3 million high quality Illumina HiSeq 4000 PE100 RNA-seq reads were obtained after sequencing of the 24 samples. After quality control and adaptor trimming, between ~19.2 and ~33,1 million reads were uniquely mapped on *S. haematobium* reference genome and counted for gene expression analysis [43]. Counted reads represented ~78% of raw reads, with 51% providing from *S. haematobium* and 48% providing from *S. bovis* (Supplementary Table S1).

The reference transcriptome assembly on which tests were carried out, was composed of 73,171 putative isoform sequences identified as TCONS, and 18,648 unique genes identified as XLOCS. We conserved the longest isoform (TCONS) for each gene (XLOC) for subsequent annotation. The GTF and Fasta file of this transcriptome are available in a Figshare repository (https://figshare.com/s/2f299e6f53c94f4a6168). Blast annotations and Gene Ontology terms of the complete reference transcriptome are available in Supplementary File S1, Sheet 1. On the 18,648 genes, 14,414 found at least one hit following Blastx analysis, and 12,332 of them were mapped to at least one GO term using Blast2GO [44].

Differential gene expression

Quantification of read abundance as well as differential gene expression analysis were performed on the 18,648 genes for each homo- and hetero-specific condition (Supplementary File S1, Sheet 2 and 3-6, respectively). The heatmap of the sample-to-sample distances as well

as the principal component analysis plot are presented in Fig 2. A total of 1,277 genes (~7%) were differentially expressed in at least one of the four homo- *vs*. hetero-specific comparisons with a FDR <5% (Supplementary File S1, Sheet7). Of these, 1,234 (97%) had a match using Blastx against the non-redundant database of the NCBI and 1,088 (85%) were mapped and successfully annotated with at least one GO term using Blast2GO [44] (Supplementary File S1 Sheet 7).

Fig 2. Differentiation between the samples in term of gene expression profiles. Principal component plot of the samples a) and Heatmap of the sample-to-sample distances b).

Most of the differentially expressed genes were identified in *S. haematobium* males, with 1,166 genes differentially expressed between the hetero-specific and homo-specific pairing conditions (734 over-expressed and 432 under-expressed in hetero-specific paired males compared to homo-specific ones). Log2-Fold changes were quite low with only one of these 1,166 DEG having a Log2-Fold Change higher than 1.5 and none had Log2-Fold change lower than -1.5 (Fig 3, Supplementary File S1 Sheet 7). In *S. haematobium* females 47 genes were differentially expressed between hetero- vs. homo-specific conditions (22 over-expressed and 25 under-expressed in hetero-specific females). Among these 47 DEG, 6 had Log2-Fold changes higher than 1.5 and one had a Log2-Fold change lower than -1.5 (Fig 3, Supplementary File S1 Sheet 7). In *S. bovis* females, 88 genes were differentially expressed between hetero- vs. homo-specific conditions (58 over-expressed and 30 under-expressed in hetero-specific females). Among these 88 DEG, 48 had Log2-Fold changes higher than 1.5 and 11 had Log2-Fold changes lower than -1.5 (Fig 3, Supplementary File S1 Sheet 7). Finally, no genes differentially expressed were identified in *S. bovis* males. Significantly (*p*<5%) over- and under-expressed genes (XLOC) for each comparison as well as their annotation are synthetized in Supplementary File S1, Sheet 7.

Fig 3. Genes expression profile in hetero-specifically compared to homo-specifically paired worms. Volcano plots showing the log transformed adjusted p-values (*i.e.*, FDR) and the log fold changes for the 18,648 unique genes of the reference transcriptome assembly for *S. haematobium* males *a*), *S. haematobium* females b), *S. bovis* females c) and *S. bovis* males d). Black dots refer to non-significant genes regarding their expression profile (over an FDR of 5%). Red dots refer to differentially expressed genes at a FDR of 5%, green dots refer to differentially expressed genes at a FDR between 5% and 1% and blue dots refer to differentially expressed genes at a FDR between 1% and 1 ‰.

Gene Ontology and enrichment analysis of the differentially expressed genes

Gene ontology categories significantly enriched in either over- or under-expressed genes were found in *S. haematobium* males (Fig 4**,** Supplementary File S1, Sheet 8) whereas in *S. haematobium* females, *S. bovis* males and females (in which fewer differentially expressed genes were detected), no GO term were significantly enriched.

Fig 4. Biological processes impacted by hetero-specific pairing in males *S. haematobium.* Barplot showing the biological processes significantly enriched in differentially expressed genes, either over-expressed or under-expressed in hetero-specific condition compared to homo-specific condition, in *S. haematobium* males.

In *S. haematobium* males, Biological processes enriched in under-expressed genes (in hetero-specific paired males compared to homo-specific ones) were related to signal transduction, notably through neuronal processes (synaptic transmission, cholinergic, chemical synaptic transmission, postsynaptic, G protein−coupled receptor signaling pathway), development (anatomical structure development), metabolism (glycogen biosynthetic process, negative regulation of endopeptidase activity), transmembrane transport (potassium ion transmembrane transport), response to stimuli (response to drug, peptidyl−proline hydroxylation, cell redox homeostasis) and cell adhesion (homophilic cell adhesion via plasma membrane adhesion molecules) (Fig 4**,** Supplementary File S1, Sheet 8).

On the other hand, Biological processes enriched in over-expressed genes (in hetero-specific males) were related to signal transduction including again some neuronal processes (*e.g.*, transmembrane receptor protein tyrosine kinase signaling pathway, regulation of Ras protein signal transduction, regulation of axon extension), metabolism (*e.g.*, proteolysis involved in cellular protein catabolic process, phosphatidylcholine metabolic process, long–chain fatty acid metabolic process, lipid droplet organization), response to stimuli (*e.g.*, response to other organism, phagocytosis, cellular response to chemical stimulus), transmembrane transport (*e.g.*, anion transmembrane transport, vesicle fusion, regulation of vesicle–mediated transport, inorganic cation import across plasma membrane, exocytosis, positive regulation of Notch signaling pathway), localization (e.g., establishment of localization in cell), locomotion (*e.g.*, regulation of locomotion, microtubule–based process, actin filament organization) and also cell adhesion (*e.g.*, cell junction assembly) (Fig 4**,** Supplementary File S1, Sheet 8).

No GO terms were found enriched neither in over- nor under-expressed genes in *S. bovis and haematobium* hetero- vs. homo-specifically paired females (Supplementary file S1, Sheet 7). However, based on annotations, in *S. haematobium* females, we found differentially expressed genes that corresponded to genetic mobile elements (*e.g.*, XLOC_014282: integrase core domain, XLOC_014741: TPA: endonuclease-reverse transcriptase, XLOC_009783: endonuclease-reverse transcriptase), genes involved in transmembrane transport (*e.g.*, XLOC_009318: phosphatase methylesterase 1 (S33 family) and XLOC_010891: Calcium-binding mitochondrial carrier S -1), stress response including oxidation-reduction processes (*e.g.*, XLOC_017856: heat shock, XLOC_012518: epidermal retil dehydrogese 2 and XLOC_018492: iron-dependent peroxidase) and other functions such as reproduction, or development (*e.g.*, XLOC_015776: egg CP391S, XLOC_007823: Craniofacial development 2). Similarly, in *S. bovis* females, we found differentially expressed genes that corresponded to genetic mobile elements as well (*e.g.*, XLOC_017328: R-directed D polymerase from

transposon X-element, XLOC_018050: R-directed D polymerase from mobile element jockey-like or XLOC_018156: gag-pol poly), genes involved ion transport (*e.g.,* XLOC_008268: Bile salt export pump, XLOC_003851: sodium-coupled neutral amino acid transporter 9 isoform X2 and XLOC_005754: Y+L amino acid transporter), response to stress (*e.g.,* XLOC_017856: heat shock and XLOC_009339: Universal stress) and as well other function such as reproduction, growth or metabolism (*e.g.,* XLOC_014939: early growth response, XLOC_015393: Syptotagmin-1, XLOC_016728: egg CP391S-like and XLOC_012591: Cathepsin B-like cysteine proteinase precursor). Hence, for *S. haematobium* and *S. bovis* females, differentially expressed genes were quite similar in term of function, regardless of their expression profile (under- or over-expression in hetero-specific pairs) and regardless of the schistosome species.

Discussion

In this study we aimed to investigate potential reproduction isolation mechanisms in two schistosome species causing major debilitating parasitic diseases worldwide and that show evidences for extensive hybridization in nature [34,45]. Specifically, we tested whether hybridization between *S. haematobium* and *S. bovis* could be constrained or promoted by mate choices and whether these mate choices are associated with specific transcriptomic profiles in hetero- and homo-specifically paired individuals. We overall showed that mate choice of *S. haematobium* and *S. bovis* occurred in a random fashion and seems to depend only on partner relative abundances. Also, we did not detect any major transcriptomic changes in hetero-specifically paired males and females *S. haematobium* and *S. bovis* compared to homo-specifically paired individuals. These results suggest that these two species lack any other pre-zygotic barrier than habitat isolation by the host which would make them more prone to hybridize.

In this work, first, we showed that the two frequently co-endemic species *S. haematobium* and *S. bovis* readily pair with no preferences for neither homo-specific nor hetero-specific associations in simultaneous infections. The only exception was found in *S. haematobium* male's choice that appeared to be biased toward hetero-specific pairing. This observation may reveal *S. haematobium* male's preference for the *S. bovis* females or a higher competence of *S. bovis* females, which may be better at pairing compared to *S. haematobium* females. Such results were for instance previously found for *S. haematobium* males when competing with *S. intercalatum* [27], S. *mattheii* [46] or *S. mansoni* [47]. However, since the bias toward hetero-specific pairing in the experiment Exp. 1 was mainly due to abundant hetero-specific pairs in only one hamster over the five used, this result should be considered with caution. Further caution is also required since the full choice experiment did not confirm such a bias toward hetero-specific pairing with *S. bovis* females. Consequently, our different mate choice experiments (limited and full choice) rather indicate no differences in competitiveness and that *S. haematobium* and *S. bovis* males and females tend to mate randomly, which suggest that there is no behavioural barriers preventing hetero-specific pairing once both species co-infect the same definitive host.

The second part of this study aimed to assess the transcriptomic profiles associated with hetero-specific and homo-specific pairings between *S haematobum* and *S. bovis*. Since different species might constitute a different stimulus for the other partner, we expected at first to find a strong impact of the hetero-specific pairing, and especially on female transcriptomes compared to males since they respond to males' stimuli for their sexual maturation [21]. However, only few differentially expressed genes (*i.e.,* from zero for *S. bovis* mate choice to 1 166 for *S. haematobium* mate choice*)* were observed in both male and females. In particular, no differentially expressed genes were identified in *S. bovis* males depending on homo- *vs.* hetero-specific pairing, and biological processes enriched in differentially expressed genes were

identified only for *S. haematobium* male pairing. Also, most of differentially expressed genes identified presented low Log2-Fold changes (notably in *S. haematobium* males where only one DEG exceeded a Log2-Fold change of 1.5). Thus, the influence of hetero-specific pairing on male and female adult worms of both species in terms of number of differentially expressed genes, related biological processes and gene expression level was not striking. Such a result suggests that both species may be highly receptive to each other since no major transcriptomic adjustments are induced by hetero-specific pairings. This observation is hence consistent with our precedent mating experiments that suggest random pairing between both species and further show that there is no major physiological nor molecular barriers making hetero-specific pairings less prone to occur.

Although pairings between male and female *S. haematobium* and *S. bovis* did not result in many differentially expressed genes, males *S. haematobium* appeared to be the most affected by the pairing status. More differentially expressed genes identified in males, and in particular more over-expressed genes in males involved in hetero-specific pairs (*i.e.*, 734 over-expressed whereas only 432 were under-expressed) was somehow puzzling. Generally, during mating more transcriptomic changes have been identified in females compared to males [14,48,49]. Our result might thus be due to a lower variability in the transcriptomic profiles of the different biological replicates of *S. haematobium* males in comparison to others simply technically easing the identification of differentially expressed genes. Also, it is worth noting that although *S. haematobium* males had more genes differentially expressed, females had overall higher log2 Fold Change for their differentially expressed genes. Alternatively, although non-exclusive, males could indeed display a more transcriptomic adjustments than females, for instance in order to properly initiate female maturation depending on their species. Nevertheless, since we did not identify any differentially expressed genes in *S. bovis* males, and also because the log2-Fold change of the DEG identified in *S. haematobium* males were low, it seems difficult to

conclude that one or the other sex is preferentially impacted during hetero-specific pairing, or that one species is more prone to initiate females species sexual maturation. Our results rather suggest that overall, only few transcriptomic adjustments are associated with hetero-specific pairing between *S. bovis* and *S. haematobium.*

Among these transcriptomic adjustments associated with the pairing status of the worms, it is worth noting that some genes and processes had functions that could be linked to male-female interactions notably linked to reproductive functions as suggested by other studies. Notably, among female schistosomes, we found three genes encoding egg proteins that were differentially expressed in *S. haematobium* and/or *S. bovis* females, and that are well-known female-associated gene products [50]. Similarly, a transcript matching the Syptotagmin-1 gene was under-expressed in hetero-specifically paired females *S. bovis.* Syptotagmin-1 is notably known to have a female-specific expression as well as to be regulated during pairing [48]. Finally, we found two differentially expressed genes in females *S. bovis* matching with cathepsin B and L, two genes that encode digestive enzymes specifically expressed by females when pairing [50]. Similarly, in *S. haematobium* males, we found several genes and biological processes related to male-female interaction. Indeed the first insights into the molecular basis of male and female interaction with a particular interest in the pairing process, proliferation, differentiation and maturation of female gonads have underlined the major role of signal transduction cascades and particularly signalling pathways such as the TGF-beta and Ras (*e.g.*, receptor tyrosine kinase coupled pathway) signalling pathways [51–57]. Also, those pathways, notably the TGF-beta signaling pathway are known to induce the production of the gynecophoric canal protein by males during pairing which is a trigger for females maturation [52]. Interestingly, in this work, among genes whose expression was affected by homo- *vs.* hetero-specific pairing in *S. haematobium* males, we notably found the TGF-beta signal transducer gene but also two gynecophoral canal protein genes, and both transmembrane

receptor protein tyrosine kinase signaling pathway and regulation of Ras protein signal transduction processes were enriched in over-expressed genes in hetero-specific pairs. Also echoing more recent studies on the gonad specific and pairing-dependent transcriptomes of male schistosomes, we found several biological processes enriched either in over- or under-expressed genes in *S. haematobium* males that were involved in neuronal processes which have been shown to be associated with such male-female interaction patterns [14,49]. We consequently found that genes and processes impacted by homo- vs hetero-specific pairing in *S. haematobium and S. bovis* at least partly overlapped those generally affected during male-female regular homo-specific interactions. These results suggest that both species may have maintained similar patterns of interactions between males and females allowing them to reproduce. A moderate regulation of these genes during pairing with another species may thus allow quite easily the two parasite species to overcome their relatedness and divergence resulting in successful hetero-specific mating.

However, it is worth noting that among the differentially expressed genes identified, the majority of them was also related to processes that were not particularly documented to be impacted during male-female interactions (*e.g.*, genetic mobile elements, response to drug and stimuli, oxidation-reduction, cell-adhesion, metabolism). This hence suggests that genes impacted during homo- vs hetero-specific pairings in schistosomes also rely on processes that exceed male-female interaction and that notably seem related to stress response. These functions related to stimuli responses (that include oxidation-reduction processes as well as the genetic mobile elements we found, [58,59]), could indicate that at least at the molecular level schistosome species may respond to an hetero-specific signal as a stress although this does not seem to impede the success of such an hetero-specific mating. Alternatively, the pairing status (*i.e.*, homo-specific or hetero-specific) could impact worms' response to external stimuli including host stimuli. In particular hetero-specific *S. haematobium* males when compared to

homo-specific males, under-expressed a fair amount of genes involved in response to drugs (*e.g.*, multidrug and toxin extrusion, Multidrug and toxin extrusion 2, Multidrug resistance or Multidrug resistance-associated), which may raise important questions regarding schistosome drug response in the context of co-infection and hybridization. More generally, many other genes potentially involved in the encounter, interaction, and communication of the two species genomes have arisen from this work. Further attention is thus needed to decipher the role of each of them in a hybridization and speciation context.

More generally, our work opens new avenue regarding the understanding of the mechanisms allowing or preventing hybridization between schistosome species. Several recent studies have presented evidences of introgression between *S. haematobium* and *S. bovis* [36,37,60]. Our main finding of a lack of proper pre-zygotic barrier preserving the genetic integrity of *S. haematobium* and *S. bovis* is strongly in line with these recent observations. Actually, the two sister species *S. haematobium* and *S. bovis* have diverged relatively recently compared to other schistosomes, thus the genetic distance between both species is probably not sufficient to avoid successful interbreeding and both species genomes are presumably highly permeable to each other's alleles [61]. The karyotype of species is also important regarding the success of hybridization. Importantly *S. haematobium* and *S. bovis* have retained the same karyotype with n=8 chromosome pairs including sex chromosomes that are morphologically similar [62]. To produce viable offspring, both species could have thus retained similar processes allowing them to find their partner in the host, pair and reproduce. An important requirement for schistosomes to reproduce is their ability to locate each other in the hosts. In the case of hetero-specific pairing, the ability of the two partners to encounter and locate each other in the same host was originally thought to constitute the main mechanism allowing *S. haematobium* and *S. bovis* to keep their genetic integrity. Also, these two species live in different host compartments (*i.e.*, *S. haematobium* lives in the urogenital vessels whereas *S. bovis* lives in the mesenteric vessels)

this tropism differences could as well contribute to make their encounter less likely within the same host. Although this barrier by the host might exist it nevertheless seems to be weak since hybrids can form easily in hamsters. Hence, the lack of any further pre-zygotic barrier implies that, in area where *S. haematobium* and *S. bovis* are co-occurring as well as their respective hosts, hybrids and introgressed individuals should be more likely to be found. So far introgression between *S. haematobium* and *S. bovis* is thought to be the result of an ancient event rather than an ongoing process [60]. However, since such hybrids may present heterosis and be more virulent compared to their parental species individuals, the understanding of the consequences of schistosome hybridization is a necessary next step to better anticipate the consequences in terms of disease dynamics and spread.

In conclusion, in this integrative study of *S. haematobium* and *S. bovis* behavioural and physiological isolation mechanisms we showed that natural hybridization between *S. haematobium* and *S. bovis* lack any strong pre-zygotic barrier but the one conditioned by their host specificity. Our data suggest that no mate recognition system mitigate hybridization between these two species and that no major transcriptomic adjustments are associated with hetero-specific pairings. This suggests that the two species remain sufficiently coadapted to each other to allow an efficient reproduction once in contact. This weak pre-zygotic barrier we exemplified may thus partly explain the high prevalence of such hybrids and echoes recent evidence of introgression between the two species. Because such hybrids may display an higher fitness compared to pure species, one could expect to find increased prevalence and intensities of schistosomiasis in areas where hybridization occurs which could run the risk of the spread of the disease in new environments. Ultimately, this could lead to new issues in the disease control, and alteration in the efficacy of current chemotherapeutic treatments.

Acknowledgments

This work was supported by the program HySWARM (ANR-18-CE35-0001) from the French Research National Agency and the Occitania region (MOLRISK). We would like to acknowledge Génome Québec and University McGill innovation center as well as Roscoff Bioinformatics platform ABiMS (http://abims.sb-roscoff.fr) and NGS facility at the bio-environment platform (University of Perpignan). This study is set within the framework of the "Laboratoires d'Excellence (LABEX)" TULIP (ANR-10-LABX-41).

References

1. Barton N, Bengtsson BO. The barrier to genetic exchange between hybridising populations. Heredity. 1986;57: 357–376. doi:10.1038/hdy.1986.135

2. Nosil P. Ecological speciation. Oxford University Press; 2012.

3. Barnard-Kubow KB, Galloway LF. Variation in reproductive isolation across a species range. Ecol Evol. 2017;7: 9347–9357. doi:10.1002/ece3.3400

4. Coyne JA, Orr HA. “Patterns of Speciation in Drosophila” Revisited. Evolution. 1997;51: 295. doi:10.2307/2410984

5. Kirkpatrick M, Ravigné V. Speciation by Natural and Sexual Selection : Models and Experiments. Am Nat. 2002;159: S22-35. doi:10.1086/338370

6. Windsor DA. Controversies in parasitology, Most of the species on Earth are parasites. Int J Parasitol. 1998;28: 1939–1941. doi:https://doi.org/10.1016/S0020-7519(98)00153-2

7. Henrich T, Kalbe M. The role of prezygotic isolation mechanisms in the divergence of two parasite species. BMC Evol Biol. 2016;16: 1–10. doi:10.1186/s12862-016-0799-5

8. Ramiro RS, Khan SM, Franke-Fayard B, Janse CJ, Obbard DJ, Reece SE. Hybridization and pre-zygotic reproductive barriers in *Plasmodium*. Proc R Soc B Biol Sci. 2015;282. doi:10.1098/rspb.2014.3027

9. Léger E, Webster JP. Hybridizations within the Genus *Schistosoma*: implications for evolution, epidemiology and control. Parasitology. 2016; 1–16. doi:10.1017/S0031182016001190

10. Colley DG, Bustinduy AL, Secor WE, King CH. Human schistosomiasis. Lancet Lond Engl. 2014;383: 2253–2264. doi:10.1016/S0140-6736(13)61949-2

11. Loker ES, Brant S V. Diversification, dioecy and dimorphism in schistosomes. Trends Parasitol. 2006;22: 521–528. doi:10.1016/j.pt.2006.09.001

12. Basch PF. Why do schistosomes have separate sexes? Parasitol Today. 1990;6: 160–163. doi:10.1016/0169-4758(90)90339-6

13. Moné H, Boissier J. Sexual biology of schistosomes. Adv Parasitol. 2004;57: 89–189. doi:10.1016/S0065-308X(04)57002-1

14. Lu Z, Sessler F, Holroyd N, Hahnel S, Quack T, Berriman M, et al. Schistosome sex matters: A deep view into gonad-specific and pairing-dependent transcriptomes reveals a complex gender interplay. Sci Rep. 2016;6: 1–14. doi:10.1038/srep31150

15. BOISSIER J, MONÉ H. Experimental observations on the sex ratio of adult *Schistosoma mansoni*, with comments on the natural male bias. Parasitology. 2000;121: 379–383.

16. Beltran S, Boissier J. Male-biased sex ratio: why and what consequences for the genus *Schistosoma*? Trends Parasitol. 2010;26: 63–69. doi:10.1016/j.pt.2009.11.003

17. Beltran S, Boissier J. Schistosome monogamy: who, how, and why? Trends Parasitol. 2008;24: 386–391. doi:10.1016/j.pt.2008.05.009

18. Beltran S, Boissier J. Are schistosomes socially and genetically monogamous? Parasitol Res. 2009;104: 481–483. doi:10.1007/s00436-008-1225-8

19. Beltran S, Cézilly F, Boissier J. Genetic dissimilarity between mates, but not male heterozygosity, influences divorce in schistosomes. PLoS ONE. 2008;3. doi:10.1371/journal.pone.0003328

20. Popiel I. Male-stimulated female maturation in *Schistosoma*: A review. J Chem Ecol. 1986;12: 1745–1754. doi:10.1007/BF01022380

21. Popiel I, Cioli D, Erasmus DA. The morphology and reproductive status of female *Schistosoma mansoni* following separation from male worms. Int J Parasitol. 1984;14: 183–190. doi:https://doi.org/10.1016/0020-7519(84)90047-X

22. Clough ER. Morphology and reproductive organs and oogenesis in bisexual and unisexual transplants of mature *Schistosoma mansoni* females. J Parasitol. 1981;67: 535–539.

23. Cornford EM, Fitzpatrick AM. The mechanism and rate of glucose transfer from male to female schistosomes. Mol Biochem Parasitol. 1985;17: 131–141. doi:https://doi.org/10.1016/0166-6851(85)90012-X

24. Loverde PT, Chen L. Schistosome female reproductive development. Parasitol Today Pers Ed. 1991;7: 303–8. doi:10.1016/0169-4758(91)90263-N

25. LoVerde PT, Niles EG, Osman A, Wu W. *Schistosoma mansoni* male–female interactions. Can J Zool. 2004;82: 357–374. doi:10.1139/z03-217

26. Kunz W. Schistosome male-female interaction: induction of germ-cell differentiation. Trends Parasitol. 2001;17: 227–231. doi:10.1016/S1471-4922(01)01893-1

27. Southgate VR, Tchuenté LAT, Vercruysse J, Jourdane J. Mating behaviour in mixed infections of *Schistosoma haematobium* and *S. mattheei*. Parasitol Res. 1995;81: 651–656.

28. Tchuente LAT, Imbert-Establet D, Delay B, Jourdane J. Choice of mate, a reproductive isolating mechanism between *Schistosoma intercalatum* and *S. mansoni* in mixed infections. Int J Parasitol. 1993;23: 179–185. doi:10.1016/0020-7519(93)90139-P

29. Cosgrove CL, Southgate VR. Mating interactions between *Schistosoma mansoni* and *S. margrebowiei*. Parasitology. 2002;125: 233–243.

30. Webster BL, Southgate VR, Tchuem Tchuenté L-A. Mating interactions between *Schistosoma haematobium* and *S. mansoni*. J Helminthol. 2007/02/01. 1999;73: 351–356. doi:DOI: 10.1017/S0022149X99000591

31. Brémond P, Sellin B, Sellin E, Naméoua B, Labbo R, Théron A, et al. Arguments for the modification of the genome (introgression) of the human parasite *Schistosoma haematobium* by genes from *S. bovis*, in Niger. C R Acad Sci III. 1993;316: 667–670.

32. Huyse T, Webster BL, Geldof S, Stothard JR, Diaw OT, Polman K, et al. Bidirectional introgressive hybridization between a cattle and human schistosome species. PLoS Pathog. 2009;5. doi:10.1371/journal.ppat.1000571

33. Webster BL, Diaw OT, Seye MM, Webster JP, Rollinson D. Introgressive Hybridization of *Schistosoma haematobium* Group Species in Senegal: Species Barrier Break Down between Ruminant and Human Schistosomes. PLoS Negl Trop Dis. 2013;7. doi:10.1371/journal.pntd.0002110

34. Moné H, Holtfreter MC, Allienne JF, Mintsa-Nguéma R, Ibikounlé M, Boissier J, et al. Introgressive hybridizations of *Schistosoma haematobium* by *Schistosoma bovis* at the origin of the first case report of schistosomiasis in Corsica (France, Europe). Parasitol Res. 2015;114: 4127–4133. doi:10.1007/s00436-015-4643-4

35. Angora EK, Allienne J-F, Rey O, Menan H, Touré AO, Coulibaly JT, et al. High prevalence of *Schistosoma haematobium* × *Schistosoma bovis* hybrids in schoolchildren in Côte d'Ivoire. Parasitology. 2020;147: 287–294. doi:10.1017/S0031182019001549

36. Savassi BAES, Mouahid G, Lasica C, Mahaman S-DK, Garcia A, Courtin D, et al. Cattle as natural host for *Schistosoma haematobium* (Bilharz, 1852) Weinland, 1858 x *Schistosoma bovis* (Sonsino, 1876) interactions, with new cercarial emergence and genetic patterns. Parasitol Res. 2020; 2189–2205. doi:10.1007/s00436-020-06709-0

37. Oey H, Zakrzewski M, Gravermann K, Young ND, Korhonen PK, Gobert GN, et al. Whole-genome sequence of the bovine blood fluke *Schistosoma bovis* supports interspecific hybridization with *S. haematobium.* Platt RN, editor. PLOS Pathog. 2019;15: e1007513. doi:10.1371/journal.ppat.1007513

38. Pitchford RJ. A check list of definitive hosts exhibiting evidence of the genus *schistosoma* (Weinland, 1858) acquired naturally in Africa and the Middle East. J Helminthol. 1977;51: 229–251. doi:10.1017/S0022149X00007574

39. Rollinson D. The genus Schistosoma: a taxonomic appraisal. Biol Schistosomes Genes Latrines. 1987.

40. Pitchford RJ, Visser PS. The role of naturally infected wild rodents in the epidemiology of schistosomiasis in the Eastern Transvaal. Trans R Soc Trop Med Hyg. 1962;56: 126–135. doi:10.1016/0035-9203(62)90139-6

41. Catalano S, Sène M, Diouf ND, Fall CB, Borlase A, Léger E, et al. Rodents as Natural Hosts of Zoonotic *Schistosoma* Species and Hybrids: An Epidemiological and Evolutionary Perspective From West Africa. J Infect Dis. 2018; jiy029. doi:10.1093/infdis/jiy029

42. Webster JP, Gower CM, Knowles SCL, Molyneux DH, Fenton A. One health - an ecological and evolutionary framework for tackling Neglected Zoonotic Diseases. Evol Appl. 2016;9: 313–333. doi:10.1111/eva.12341

43. Young ND, Jex AR, Li B, Liu S, Yang L, Xiong Z, et al. Whole-genome sequence of *Schistosoma haematobium*. Nat Genet. 2012;44: 221–225. doi:10.1038/ng.1065

44. Conesa A, Götz S, García-Gómez JM, Terol J, Talón M, Robles M. Blast2GO: a universal tool for annotation, visualization and analysis in functional genomics research. Bioinformatics. 2005;21: 3674–3676.

45. Leger E, Webster JP. Hybridizations within the Genus *Schistosoma* : implications for evolution, epidemiology and control. Parasitology. 2017;144: 65–80. doi:10.1017/S0031182016001190

46. Webster BL, Southgate VR. Mating interactions of *Schistosoma haematobium* and *S. intercalatum* with their hybrid offspring. Parasitology. 2003;126: 327–338.

47. Webster BL, Southgate VR, Tchuenté LAT. Mating interactions between *Schistosoma haematobium* and *S. mansoni*. J Helminthol. 1999;73: 351–356.

48. Grevelding CG, Sommer G, Kunz W. Female-specific gene expression in *Schistosoma mansoni* is regulated by pairing. Parasitology. 1997;115: 635–640. doi:10.1017/S0031182097001728

49. Leutner S, Oliveira KC, Rotter B, Beckmann S, Buro C, Hahnel S, et al. Combinatory Microarray and SuperSAGE Analyses Identify Pairing-Dependently Transcribed Genes in *Schistosoma mansoni* Males, Including Follistatin. Jones MK, editor. PLoS Negl Trop Dis. 2013;7: e2532. doi:10.1371/journal.pntd.0002532

50. Hoffmann KF. An historical and genomic view of schistosome conjugal biology with emphasis on sex-specific gene expression. Parasitology. 2004;128: S11–S22. doi:10.1017/S0031182004006213

51. LoVerde PT, Andrade LF, Oliveira G. Signal transduction regulates schistosome reproductive biology. Curr Opin Microbiol. 2009;12: 422–428. doi:10.1016/j.mib.2009.06.005

52. Osman A, Niles EG, Verjovski-Almeida S, LoVerde PT. *Schistosoma mansoni* TGF-β receptor II: Role in host ligand-induced regulation of a schistosome target gene. PLoS Pathog. 2006;2: 0536–0550. doi:10.1371/journal.ppat.0020054

53. LoVerde PT, Osman A, Hinck A. Schistosoma mansoni: TGF-β signaling pathways. Exp Parasitol. 2007;117: 304–317. doi:10.1016/j.exppara.2007.06.002

54. Knobloch J, Beckmann S, Burmeister C, Quack T, Grevelding CG. Tyrosine kinase and cooperative TGFβ signaling in the reproductive organs of *Schistosoma mansoni*. Exp Parasitol. 2007;117: 318–336. doi:10.1016/j.exppara.2007.04.006

55. Beckmann S, Quack T, Burmeister C, Buro C, Long T, Dissous C, et al. *Schistosoma mansoni*: Signal transduction processes during the development of the reproductive organs. Parasitology. 2010;137: 497–520. doi:10.1017/S0031182010000053

56. Freitas TC, Jung E, Pearce EJ. TGF-β signaling controls embryo development in the parasitic flatworm *Schistosoma mansoni*. PLoS Pathog. 2007;3: 489–497. doi:10.1371/journal.ppat.0030052

57. Andrade LF de, Mourão M de M, Geraldo JA, Coelho FS, Silva LL, Neves RH, et al. Regulation of Schistosoma mansoni Development and Reproduction by the Mitogen-Activated Protein Kinase Signaling Pathway. PLoS Negl Trop Dis. 2014;8: e2949. doi:10.1371/journal.pntd.0002949

58. Storz G, Imlayt JA. Oxidative stress. Curr Opin Microbiol. 1999;2: 188–194.

59. Capy P, Gasperi G, Biémont C, Bazin C. Stress and transposable elements: co-evolution or useful parasites? Heredity. 2000;85: 101–106. doi:10.1046/j.1365-2540.2000.00751.x

60. Platt RN, McDew-White M, Le Clec'h W, Chevalier FD, Allan F, Emery AM, et al. Ancient Hybridization and Adaptive Introgression of an Invadolysin Gene in Schistosome Parasites. Shapiro B, editor. Mol Biol Evol. 2019;36: 2127–2142. doi:10.1093/molbev/msz154

61. Kincaid-Smith J, Tracey A, de Carvalo Augusto R, Bulla I, Holroyd N, Rognon A, et al. Whole genome sequencing and morphological analysis of the human-infecting schistosome emerging in Europe reveals a complex admixture between *Schistosoma haematobium* and *Schistosoma bovis* parasites. bioRxiv. 2018. doi:10.1101/387969

62. Grossman a I, Short RB, Cain GD. Karyotype evolution and sex chromosome differentiation in Schistosomes (Trematoda, Schistosomatidae). Chromosoma. 1981;84: 413–30. doi:10.1007/BF00286030

63. Kincaid-Smith J, Boissier J, Allienne JF, Oleaga A, Djuikwo-Teukeng F, Toulza E. A Genome Wide Comparison to Identify Markers to Differentiate the Sex of Larval Stages of *Schistosoma haematobium*, *Schistosoma bovis* and their Respective Hybrids. PLoS Negl Trop Dis. 2016;10: e0005138. doi:10.1371/journal.pntd.0005138

64. Boissier J, Rivera ER, Moné H. Altered behavior of the snail *Biomphalaria glabrata* as a result of infection with *Schistosoma mansoni*. J Parasitol. 2003;89: 429–433. doi:10.1645/0022-3395(2003)089[0429:ABOTSB]2.0.CO;2

65. Boissier J, Chlichlia K, Digon Y, Ruppel A, Moné H. Preliminary study on sex-related inflammatory reactions in mice infected with *Schistosoma mansoni*. Parasitol Res. 2003;91: 144–150. doi:10.1007/s00436-003-0943-1

66. Théron a, Pages JR, Rognon a. *Schistosoma mansoni*: distribution patterns of miracidia among *Biomphalaria glabrata* snail as related to host susceptibility and sporocyst regulatory processes. Exp Parasitol. 1997;85: 1–9. doi:10.1006/expr.1996.4106

67. Beltran S, Galinier R, Allienne JF, Boissier J. Cheap, rapid and efficient DNA extraction method to perform multilocus microsatellite genotyping on all *Schistosoma mansoni* stages. Mem Inst Oswaldo Cruz. 2008;103: 501–503. doi:S0074-02762008000500017 [pii]

68. Van den Broeck F, Geldof S, Polman K, Volckaert FAM, Huyse T. Optimal sample storage and extraction procotols for reliable multilocus genotyping of the human parasite *Schistosoma mansoni*. Infect Genet Evol. 2011;11: 1413–1418. doi:10.1016/j.meegid.2011.05.006

69. Webster BL, Rollinson D, Stothard JR, Huyse T. Rapid diagnostic multiplex PCR (RD-PCR) to discriminate *Schistosoma haematobium* and *S. bovis*. J Helminthol. 2010;84: 107–114. doi:10.1017/S0022149X09990447

70. Giardine B, Riemer C, Hardison RC, Burhans R, Elnitski L, Shah P, et al. Galaxy: A platform for interactive large-scale genome analysis. Genome Res. 2005;15: 1451–1455. doi:10.1101/gr.4086505

71. Goecks J, Nekrutenko A, Taylor J, Afgan E, Ananda G, Baker D, et al. Galaxy: a comprehensive approach for supporting accessible, reproducible, and transparent computational research in the life sciences. Genome Biol. 2010;11. doi:10.1186/gb-2010-11-8-r86

72. Blankenberg D, Gordon A, Von Kuster G, Coraor N, Taylor J, Nekrutenko A, et al. Manipulation of FASTQ data with galaxy. Bioinformatics. 2010;26: 1783–1785. doi:10.1093/bioinformatics/btq281

73. Martin M. Cutadapt removes adapter sequences from high-throughput sequencing reads. EMBnet.journal. 2011;17: 10. doi:10.14806/ej.17.1.200

74. Dobin A, Davis CA, Schlesinger F, Drenkow J, Zaleski C, Jha S, et al. STAR: Ultrafast universal RNA-seq aligner. Bioinformatics. 2013;29: 15–21. doi:10.1093/bioinformatics/bts635

75. Trapnell C, Williams BA, Pertea G, Mortazavi A, Kwan G, van Baren MJ, et al. Transcript assembly and quantification by RNA-Seq reveals unannotated transcripts and isoform switching during cell differentiation. Nat Biotechnol. 2010;28: 511.

76. Anders S, Pyl PT, Huber W. HTSeq-A Python framework to work with high-throughput sequencing data. Bioinformatics. 2015;31: 166–169. doi:10.1093/bioinformatics/btu638

77. Love MI, Anders S, Huber W. Differential analysis of count data - the DESeq2 package. Genome Biology. 2014. doi:110.1186/s13059-014-0550-8

78. R Core Team. R: A language and environment for statistical computing. R Foundation for Statistical Computing, Vienna, Austria; 2020. Available: URL https://www.R-project.org/

Figure 1

Click here to access/download;Figure;Fig 1.pdf

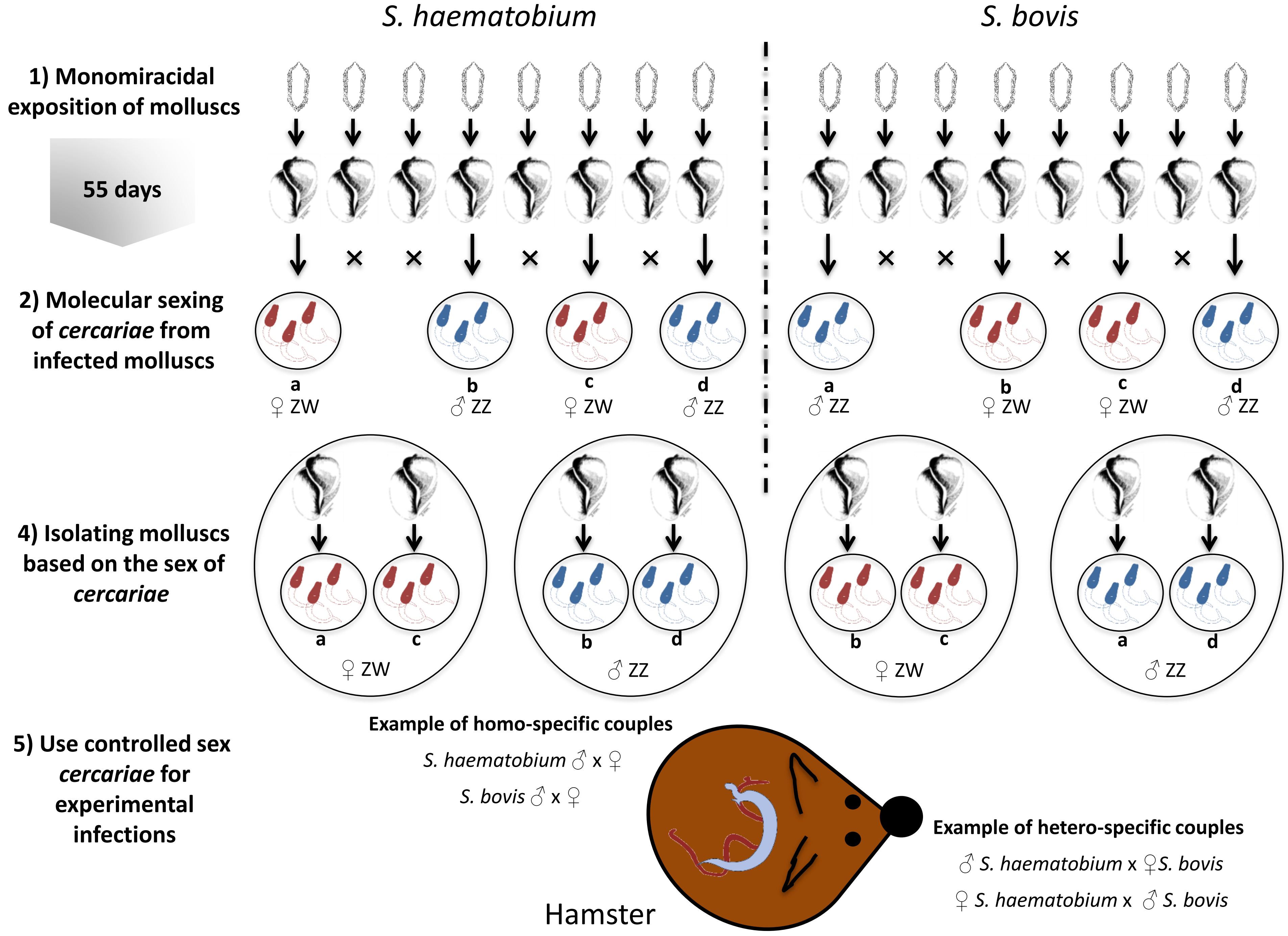

Figure 2

Click here to access/download;Figure;Fig 2.pdf

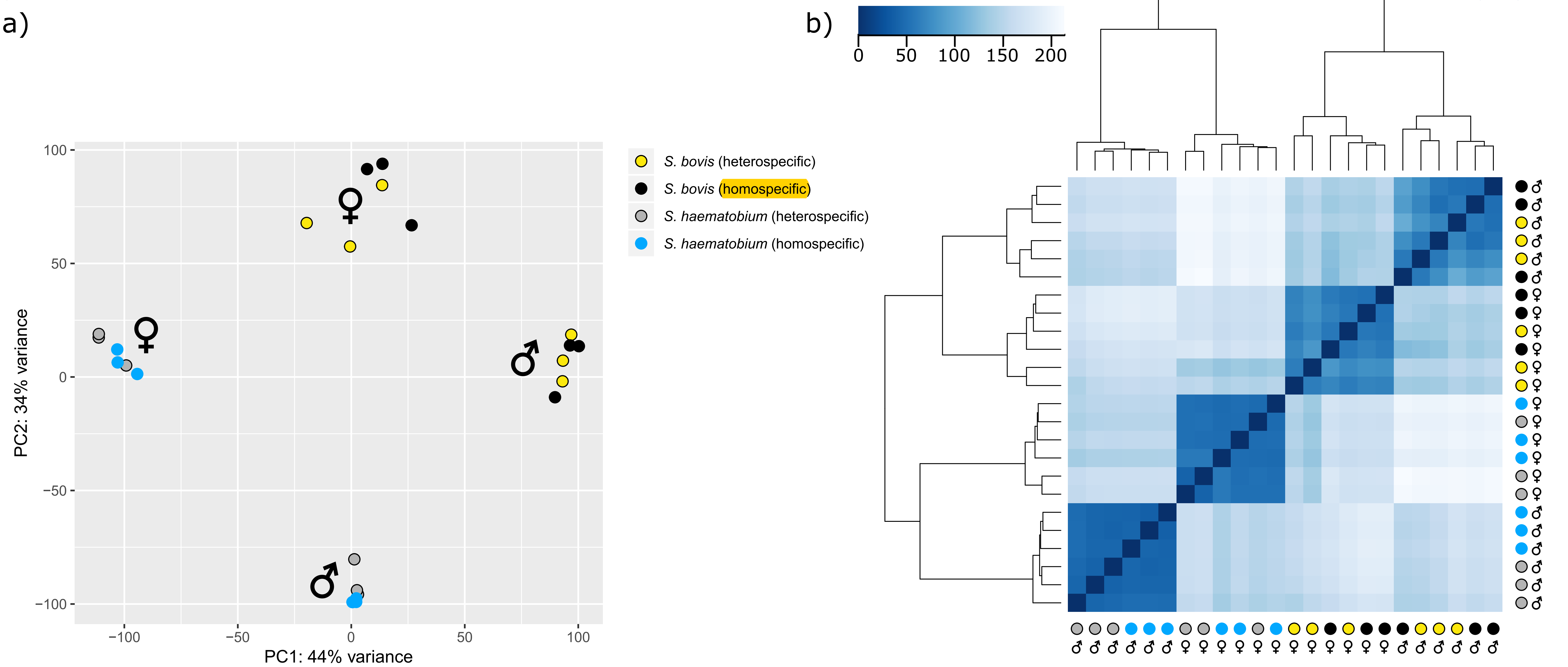

Click here to access/download;Figure;Fig 3.pdf

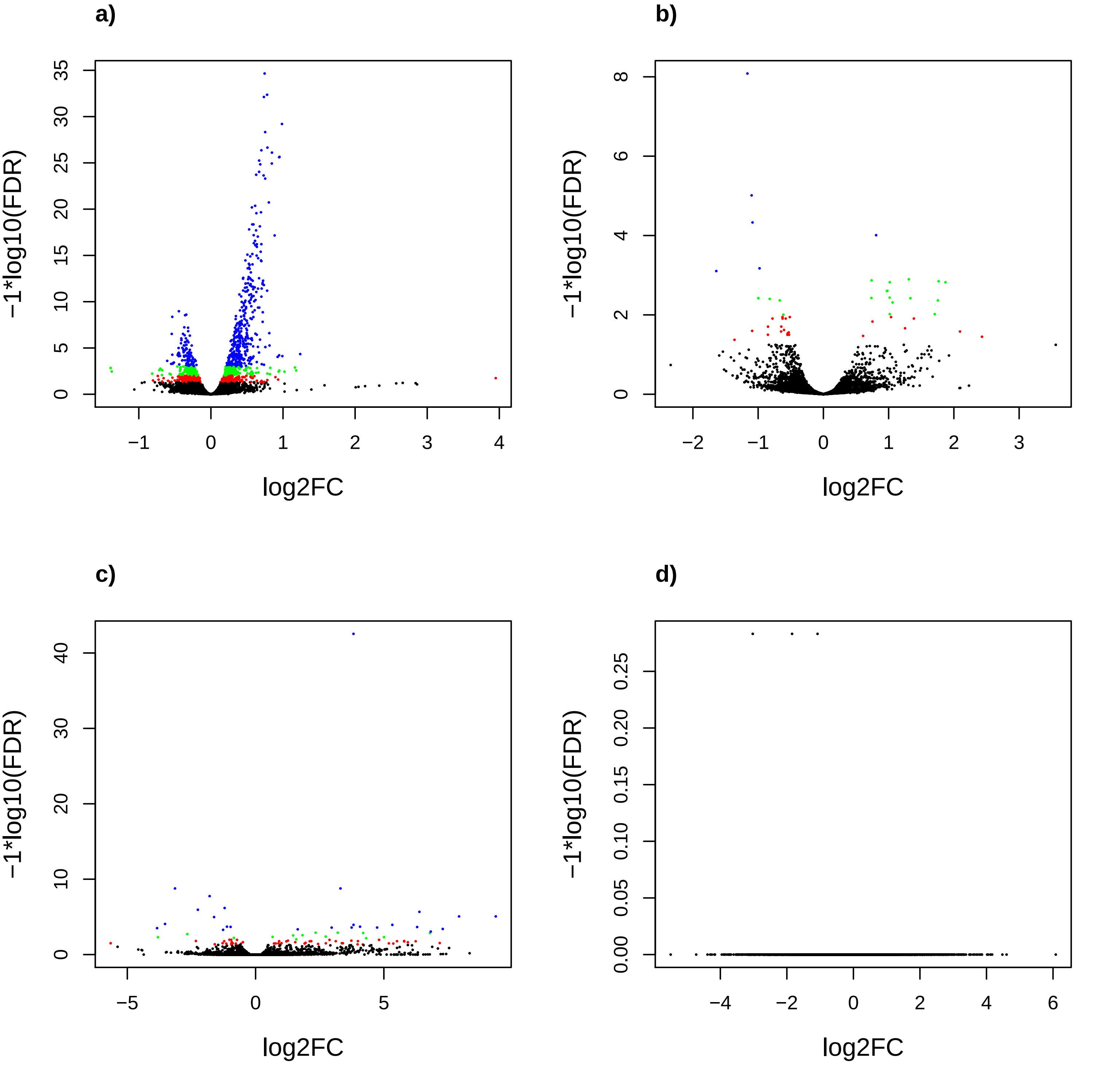

Figure 4

Click here to access/download;Figure;Fig 4.pdf

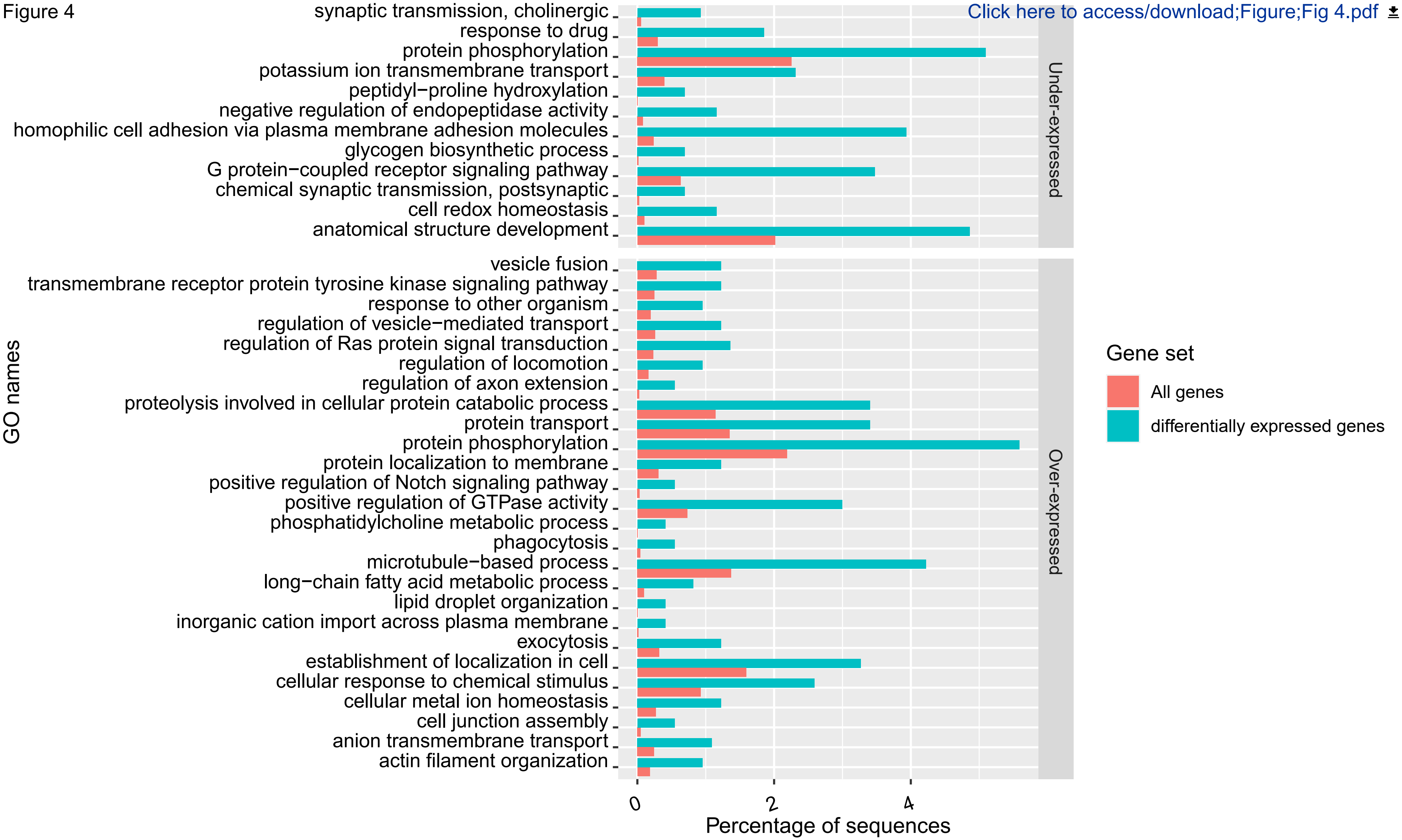

Supporting Information Table S1

Click here to access/download

Supporting Information

Supplementary Table S1.docx

Supplementary File S1

Click here to access/download
Supporting Information
Supplementary File S1.xlsx
