# Supplementary material for: No pre-zygotic isolation mechanisms between Schistosoma haematobium and Schistosoma bovis parasites: From mating interactions to differential gene expression
Source: PLoS Negl Trop Dis. 2021 May 4;15(5):e0009363. doi: 10.1371/journal.pntd.0009363 (PMC8127863; doi:10.1371/journal.pntd.0009363)
Supplement: Attachment — Submitted filename: PNTD-D-20-01594_R1.pdf [file pntd.0009363.s005.pdf]

PLOS Neglected Tropical Diseases

No pre-zygotic isolation mechanisms between Schistosoma haematobium and Schistosoma bovis parasites: from mating interactions to differential gene expression

--Manuscript Draft--

**Manuscript Number:**	PNTD-D-20-01594R1
**Full Title:**	No pre-zygotic isolation mechanisms between Schistosoma haematobium and Schistosoma bovis parasites: from mating interactions to differential gene expression
**Short Title:**	No pre-zygotic barriers between schistosome species
**Article Type:**	Research Article
**Keywords:**	Schistosoma; Hybridization; Reproductive isolation mechanisms; RNA-sequencing
**Abstract:**	Species usually develop reproductive isolation mechanisms allowing them to avoid interbreeding. These preventive barriers can act before reproduction, “pre-zygotic barriers”, or during the life cycle, “post-zygotic barriers”. Pre-zygotic barriers prevent unfavourable mating, while post-zygotic barriers determine the viability and selective success of the hybrid offspring. Hybridization in parasites and the underlying reproductive isolation mechanisms maintaining their genetic integrity have been overlooked. Using an integrated approach this work aims to quantify the relative importance of pre-zygotic barriers in Schistosoma haematobium x S. bovis crosses. These two co-endemic species cause schistosomiasis, one of the major debilitating parasitic diseases worldwide, and can hybridize naturally. Using mate choice experiments we first tested if a specific mate recognition system exists between both species. Second, using RNA-sequencing we analysed differential gene expression between homo- and hetero-specific pairing in male and female adult parasites. We show that homo- and hetero-specific pairing occurs randomly between these two species, and few genes in both sexes are affected by hetero-specific pairing. This suggests that i) mate choice is not a reproductive isolating factor, and that ii) no pre-zygotic barrier except spatial isolation “by the final vertebrate host” seems to limit interbreeding between these two species. Interestingly, among the few genes affected by the pairing status of the worms, some can be related to pathways affected during male and female interactions and may also present interesting candidates for species isolation mechanisms and hybridization in schistosome parasites.
**Additional Information:**	
**Question**	**Response**
**Financial Disclosure**<br><br>Enter a financial disclosure statement that describes the sources of funding for the work included in this submission. Review the submission guidelines for detailed requirements. View published research articles from *PLOS NTDs* for specific examples.<br><br>This statement is required for submission and **will appear in the published article** if the submission is accepted. Please make sure it is accurate.	Funding<br>This work has been funded by the French Research National Agency (project HySWARM, grant no. ANR-18-CE35-0001). SM was supported by the Occitania region (project MOLRISK, award no. NREST 2019/1/059), the European “Fonds Européen de Développement Régional” (FEDER) and MRG by the Fellowship of "Estancias breves" (linked to the Programa de Ayudas de Formacion de Profesorado Universitario 2015, Ministerio de Ciencia, Innovación y Universidades, Spain).

**Unfunded studies**<br><br>Enter: *The author(s) received no specific funding for this work.*<br><br>**Funded studies**<br><br>Enter a statement with the following details:<br>• Initials of the authors who received each award<br>• Grant numbers awarded to each author<br>• The full name of each funder<br>• URL of each funder website<br>• Did the sponsors or funders play any role in the study design, data collection and analysis, decision to publish, or preparation of the manuscript?<br>• **NO** - Include this sentence at the end of your statement: *The funders had no role in study design, data collection and analysis, decision to publish, or preparation of the manuscript.*<br>• **YES** - Specify the role(s) played.<br><br>* typeset	
**Competing Interests**<br><br>Use the instructions below to enter a competing interest statement for this submission. On behalf of all authors, disclose any competing interests that could be perceived to bias this work—acknowledging all financial support and any other relevant financial or non-financial competing interests.<br><br>This statement **will appear in the published article** if the submission is accepted. Please make sure it is accurate. View published research articles from *PLOS NTDs* for specific examples.	The authors have declared that no competing interests exist.

Powered by Editorial Manager® and ProduXion Manager® from Aries Systems Corporation

**NO authors have competing interests**<br><br>Enter: *The authors have declared that no competing interests exist.*<br><br>**Authors with competing interests**<br><br>Enter competing interest details beginning with this statement:<br><br>*I have read the journal's policy and the authors of this manuscript have the following competing interests: [insert competing interests here]*<br><br>* typeset	
**Data Availability**<br><br>Authors are required to make all data underlying the findings described fully available, without restriction, and from the time of publication. PLOS allows rare exceptions to address legal and ethical concerns. See the PLOS Data Policy and FAQ for detailed information.<br><br>A Data Availability Statement describing where the data can be found is required at submission. Your answers to this question constitute the Data Availability Statement and **will be published in the article**, if accepted.	Yes - all data are fully available without restriction

**Important:** Stating 'data available on request from the author' is not sufficient. If your data are only available upon request, select 'No' for the first question and explain your exceptional situation in the text box.<br><br>Do the authors confirm that all data underlying the findings described in their manuscript are fully available without restriction?	
**Describe where the data may be found in full sentences. If you are copying our sample text, replace any instances of XXX with the appropriate details.**<br><br>• If the data are **held or will be held in a public repository**, include URLs, accession numbers or DOIs. If this information will only be available after acceptance, indicate this by ticking the box below. For example: *All XXX files are available from the XXX database (accession number(s) XXX, XXX.*).<br>• If the data are all contained **within the manuscript and/or Supporting Information files**, enter the following: *All relevant data are within the manuscript and its Supporting Information files.*<br>• If neither of these applies but you are able to provide **details of access elsewhere**, with or without limitations, please do so. For example:<br><br>*Data cannot be shared publicly because of [XXX]. Data are available from the XXX Institutional Data Access / Ethics Committee (contact via XXX) for researchers who meet the criteria for access to confidential data.*<br><br>*The data underlying the results presented in the study are available*	Raw sequences used in this study have been submitted to the Sequence Read Archive under the BioProject PRJNA491632, the transcriptome assembly have been submitted on the Figshare repository (https://figshare.com/s/2f299e6f53c94f4a6168) and they will be released upon publication.

Powered by Editorial Manager® and ProduXion Manager® from Aries Systems Corporation

from (include the name of the third party and contact information or URL).<br>• This text is appropriate if the data are owned by a third party and authors do not have permission to share the data.<br><br>* typeset	
Additional data availability information:	Tick here if the URLs/accession numbers/DOIs will be available only after acceptance of the manuscript for publication so that we can ensure their inclusion before publication.

Powered by Editorial Manager® and ProduXion Manager® from Aries Systems Corporation

Manuscript
Click here to access/download;Manuscript;MS_resubmission_without_highli

No pre-zygotic isolation mechanisms between *Schistosoma haematobium* and *Schistosoma bovis* parasites: from mating interactions to differential gene expression

---

Julien Kincaid-Smith[1,2,§], Eglantine Mathieu-Bégné[1§], Cristian Chaparro[1], Marta Reguera-Gomez[3], Stephen Mulero[1], Jean-Francois Allienne[1], Eve Toulza[1], Jérôme Boissier[1]*

[1] IHPE, Univ. Montpellier, CNRS, Ifremer, Univ. Perpignan Via Domitia, Perpignan France.

[2] Centre for Emerging, Endemic and Exotic Diseases (CEEED), Department of Pathobiology and Population Sciences (PPS), Royal Veterinary College, University of London, Hawkshead Campus, Herts, AL9 7TA, UK.

[3] Departamento de Parasitología, Facultad de Farmacia, Universidad de Valencia, Av. Vicent Andrés Estellés s/n, 46100, Burjassot, Valencia, Spain.

* Corresponding author
§: Denotes co-first authors

Short title: No pre-zygotic barriers between schistosome species

Author contributions:

JB and ET conceptualized, supervised the study, and acquired the funding. JKS and SM carried out the experiments and conducted molecular analyses. CC, JFA curated the data. JKS, EMB, MRG performed bioinformatics and statistical analysis. JB, ET, JKS and EMB interpreted the data. JKS and EMB drafted and prepared the manuscript. JB, ET, JKS, EMB and CC revised the manuscript. All authors read and approved the final version of the manuscript.

Availability of data and materials

Raw sequences used in this study have been submitted to the Sequence Read Archive under the BioProject PRJNA491632, the transcriptome assembly have been submitted on the Figshare repository (https://figshare.com/s/2f299e6f53c94f4a6168) and they will be released upon publication.

Funding

This work has been funded by the French Research National Agency (project HySWARM, grant no. ANR-18-CE35-0001). SM was supported by the Occitania region (project MOLRISK, award no. NREST 2019/1/059), the European “Fonds Européen de Développement Régional” (FEDER) and MRG by the Fellowship of "Estancias breves" (linked to the Programa de Ayudas de Formacion de Profesorado Universitario 2015, Ministerio de Ciencia, Innovación y Universidades, Spain).

Competing interest statement

We have no competing interest.

Abstract

Species usually develop reproductive isolation mechanisms allowing them to avoid interbreeding. These preventive barriers can act before reproduction, “pre-zygotic barriers”, or during the life cycle, “post-zygotic barriers”. Pre-zygotic barriers prevent unfavourable mating, while post-zygotic barriers determine the viability and selective success of the hybrid offspring. Hybridization in parasites and the underlying reproductive isolation mechanisms maintaining their genetic integrity have been overlooked. Using an integrated approach this work aims to quantify the relative importance of pre-zygotic barriers in *Schistosoma haematobium x S. bovis* crosses. These two co-endemic species cause schistosomiasis, one of the major debilitating parasitic diseases worldwide, and can hybridize naturally. Using mate choice experiments we first tested if a specific mate recognition system exists between both species. Second, using RNA-sequencing we analysed differential gene expression between homo- and hetero-specific pairing in male and female adult parasites. We show that homo- and hetero-specific pairing occurs randomly between these two species, and few genes in both sexes are affected by hetero-specific pairing. This suggests that i) mate choice is not a reproductive isolating factor, and that ii) no pre-zygotic barrier except spatial isolation “by the final vertebrate host” seems to limit interbreeding between these two species. Interestingly, among the few genes affected by the pairing status of the worms, some can be related to pathways affected during male and female interactions and may also present interesting candidates for species isolation mechanisms and hybridization in schistosome parasites.

Keywords: Schistosoma, Hybridization, Reproductive isolation mechanisms, RNA-sequencing

Author summary

Understanding how species maintain their genetic integrity is a central question in evolutionary biology. While isolation mechanisms are well documented in free-living organisms, it is currently not the case for parasite species. Yet, occurrence of parasite hybrids is a critical global health concern since these hybrids are expected to be more harmful than parental species. We addressed the question of reproductive isolation mechanisms in parasitic species by conducting an integrative experimental study (from mate choice to gene expression) on two schistosome species (*Schistosoma haematobium* and *S. bovis*) that parasitize human and cattle, respectively. Importantly, their hybrid progeny has been involved in recent outbreaks, including outbreaks outside of endemic areas. We showed that rather than having a homo-specific mate choice, *S. haematobium* and *S. bovis* mate randomly. Also, male and female worms only express a few genes differentially when involved in a hetero-specific pair compared to a homo-specific pair. We consequently suggest that these two schistosome species lack strong reproduction isolation mechanisms, except those imposed by specificity to the final host species. Our results raise the concern that in the absence of post-zygotic barriers in sympatric zones hybridization might be more common than previously thought if these two species are able to encounter each other.

Introduction

A subset of obstacles has evolved in the course of speciation in order to limit gene flow via hybridization and maintain species boundaries. These obstacles are traditionally classified as pre- and post-zygotic barriers (also known as pre- or post-mating barriers) and can be defined as any mechanism preventing or reducing gene flow between groups of potentially interbreeding individuals [1]. Pre-zygotic barriers include spatial isolation (*e.g.*, two species live in different habitats), behavioural isolation (*e.g.*, individuals can choose to mate with individuals of their own species), temporal isolation (reproduction does not occur at the same

time *e.g.*, different seasons), mechanical isolation (sex organs are not compatible) and gametic isolation (sperm and eggs mix but fertilization does not occur). When the first barrier is crossed, post-zygotic isolation mechanisms can arise to prevent gene flow. Post-zygotic barriers include hybrid unviability (hybrids die prematurely), reduced fitness with low fertility (hybrids are less fertile, infertile or non-viable) or hybrid breakdown (a longer process where the hybrid lines are counter-selected compared to their parental forms). The strength and/or the order of each barrier vary among species which makes difficult to predict the outcome of inter-species mating, and the evolution of reproductive isolation mechanisms [2]. Moreover, reproductive isolation is often the result of an accumulation and interaction of multiple pre- and post-zygotic mechanisms restricting most gene flow [3]. However, it is generally recognized that pre-zygotic isolation barriers are enhanced in sympatric species [4], and are the most effective because they act early to prevent the production of hybrid progeny.

Despite their importance in terms of biodiversity [5], but also animal and human health, parasite species have received less attention than other free-living organisms regarding both hybridization and the role of reproductive isolation mechanisms [6]. Pre-zygotic barriers in parasites usually include additional and stronger obstacles to overcome compared to those of free-living organisms. For instance, the "habitat barrier" includes the geographic area, the host species and the tropism within the host. For parasites, hosts are dynamic habitats imposing strong selective pressures (co-evolutionary arms race) requiring constant adaptation of parasites for the completion of their life cycle. The specialisation of parasite species to a particular host is thus expected to be a strong pre-zygotic isolation mechanism preventing hybridization and favouring speciation. However, some closely related species do manage to retain their genetic identity whilst parasitizing the same host, meaning that they have acquired selective mechanisms for reproductive isolation. Hybridization and pre-zygotic reproductive barriers have been studied on very few parasite models such as plasmodium species, cestodes and

schistosomes [6–8]. Partial pre-zygotic barriers have been evidenced between *Plasmodium berghei* and *P. yoeli* [7]. It was not the case between *Schistocephalus solidus* and *S. pungitii* [6], suggesting in the latter that post-zygotic selection against hybridisation is presumably the most important driving force limiting gene flow between these two parasitic sister species [6].

Schistosomes are parasitic agents that cause schistosomiasis, a debilitating disease affecting over 240 million people worldwide, mainly in tropical and subtropical areas [9]. There are currently know species in the genus *Schistosoma*, including six species that infect humans and 19 species that infect animals [8]. These parasites have a two-host life cycle, which includes a mammalian definitive host, in which sexual reproduction occurs and a mollusc intermediate host in which asexual multiplication takes place. Schistosomes have the particularity of having separate sexes, a feature not observed in other trematodes that are hermaphrodite [10,11]. Schistosomes have therefore been intensively studied for their sexual features including male-female interactions [12,13], sex-ratios [14,15], mating systems [16,17] and mating behaviour [18]. One direct consequence of dioecism in these species is the necessity of individuals of both sexes to infect the same definitive host. This constraint can lead to interactions between species infecting the same host, and in the case of porous reproductive pre-zygotic barriers this can lead to hybridization.

To conserve their genetic identity, schistosomes that inhabit the same definitive host are expected to present pre-zygotic isolation mechanisms. Among these barriers, habitat and behavioural isolation have a great influence in schistosome’s sexual interactions. First, habitat isolation is a three-level constraint that initially has to be overcome (*i.e.*, same geographic area, same host individual, and same localisation in the host). Indeed, schistosomes species are distributed worldwide (the majority in Africa), the vertebrate host specificity depends on the parasite species, and while the majority of species live in the mesenteric vein system, one species (*S. haematobium*) lives in the veins surrounding the bladder of humans. Second,

behavioural isolation is more complex in schistosomes than in other species because mating is followed by a pairing-dependent differentiation of the female's sexual organs [12,13]. Studies have clearly established that the presence of the male (independently of the species paired) is necessary not only for the female's sexual development, but also for the maintenance of a sexually mature and active state [19–21]. It was also demonstrated that female schistosomes stimulate males through changes in levels of glutathione and lipids, and stimulate tyrosine uptake in the male worms [12]. Hence, while males transfer glucose and lipid secretions to females, females also release factors affecting the physiology of male worms [22–25]. Thus, male and female schistosomes are strongly co-dependent, in terms of behaviour (*i.e.,* they have complementary roles in the hosts), but also physiologically [10] with an intimate and permanent association between sexes necessary for reproduction to occur.

Nevertheless, several hybrid schistosomes have been evidenced [8,22,26,27]. Similarly to other groups, isolation mechanisms increase with divergence time between taxa [4,8]. The success of inter-species interactions on the viability of hybrid offspring also depends on the direction of the cross and thus which parental species provides the maternal and paternal genome [27–29]). Studies on schistosome mate choices have revealed that depending on the parasite species interacting, some combinations may readily pair with no preference (*S. haematobium x S. intercalatum, S. bovis x S. curassoni* and *S. mansoni x S haematobium*), whereas when involved in other combinations, species may present a mate recognition system favoring or not interspecies pairing (*S. mansoni x S. intercalatum, S. haematobium* x *S. mattheeii,* and *S. mansoni x S. margrebowiei* crosses) [28,30–32]. However, competition between schistosome species can also explain the frequency of some interspecific crosses [28–30]. For instance it has been shown that *S. haematobium* males can take away females from other species when competing with male *S. intercalatum* [33], S. *mattheei* [29] or *S. mansoni* [28] hence promoting or favouring hetero-specific pairing. For schistosome species that randomly pair with no mate

preference and for many related parasitic species capable of hybridizing, final host specificity may be the sole barrier preventing interbreeding [33]. This isolation mechanism "by the host" may be so efficient that species may lack any post-zygotic or other pre-zygotic mechanisms ultimately allowing them to hybridize when the opportunity arises. Therefore, the lack of reproductive incompatibility (*i.e.*, isolation by behaviour and physiology) between schistosome species infecting humans and animals may facilitate gene flow if the host isolation barriers are broken down.

Schistosoma haematobium x *S. bovis* hybrids are today the most studied hybrid system of schistosomes. These hybrids were first identified in Niger by Brémont [34] and more recently in Senegal [26] but appeared widely distributed in West Africa [26,27,34–37]. Moreover, these hybrids have recently been involved in a large-scale outbreak in Europe (Corsica, France), where transmission of the disease is persistent [35,38]. *Schistosoma haematobium* and *S. bovis* are co-endemic in Africa, but their host specificity and tropism within their definitive hosts are different (urogenital and human vs. intestinal and cattle, respectively). *S. haematobium* is mainly a parasite of human, however, sporadic studies have shown that non-human primates, *Cetartiodactyla* members or rodents could be naturally infected by this parasite species (although these accounts were based on egg morphology and could thus involve other species) [39–41]. Conversely, *S. bovis* is mainly a parasite of ruminants with sporadic cases of rodent infection [39,42]. Interestingly, although data remain scarce, recent studies showed that *S. haematobium* x *S. bovis* hybrids may naturally infect rodents or cattle [37,42].

Hybridization between these two species is particularly worrying because it raises the eventuality for a human parasite to have animal reservoirs of infection and the animal parasite to be zoonotic [43]. Likewise, hybridization may lead to changes in the parasites life history traits, including host range expansion, increased virulence and host morbidity, but also response to chemotherapeutic treatment [44]. Indeed, these hybrids often display heterosis, in which their

fitness outperforms the fitness of parental species [8,26,27]. Importantly the existence of a mate recognition system between the two species would prevent natural occurrences of hybridization in sympatric areas. In contrary a lack of reproductive isolation could indicate that occurrences of hybridization may be more frequent.

Although experimental crosses in hamsters have demonstrated their capacity to pair and the viability of *S. haematobium* x *S. bovis* hybrids [27], their pairing frequency and underlying molecular mechanisms need to be assessed. This study hence uses an integrated approach, from mating behaviour to male and female gene expression, in order to quantify the importance of pre-zygotic barriers involved in the interactions between *S. haematobium x S. bovis*. First, using a mate choice experiment we tested whether specific mate recognition or competition exists by quantifying the frequency of hetero-specific and homo-specific pairs compared to random mating expectations. Second, given the strong co-dependence between male and female schistosomes, we also analysed the influence of pairing (homo- vs hetero-specific) on the transcriptomic profile of male and female parasites using RNA sequence analysis. We hypothesize that since these hybrids are frequently encountered in the field [35,35,37] and since parental species are able to pair in the laboratory [27] mate recognition should not constitute a strong barrier to reproduction. However, depending on species dominance in mating, the direction of pairing could be affected. Since females undergo strong developmental changes upon pairing [13,45,46] we would expect finding strong transcriptomic changes associated with inter-species interactions for females but not for males. The molecular determinants of the very first step towards hybridization may give further insight into the permeability of the two species and reveal some important genes linked to male and female interaction, species isolation and hybridization.

Materiel and methods

Ethics Statement

Experiments were carried out according to national ethical standards established in the writ of February 1st, 2013 (NOR: AGRG1238753A), setting the conditions for approval, planning and operation of establishments, breeders and suppliers of animals used for scientific purposes and controls. The French Ministry of Agriculture and Fishery (Ministère de l'Agriculture et de la Pêche), and the French Ministry for Higher Education, Research and Technology (Ministère de l'Education Nationale de la Recherche et de la Technologie) approved the experiments carried out for this study and provided permit A66040 for animal experimentation. The investigator possesses the official certificate for animal experimentation delivered by both ministries (Décret n° 87–848 du 19 octobre 1987; number of the authorization 007083).

Origin and maintenance of schistosome strains

Schistosoma haematobium and *S. bovis* were maintained in the laboratory using *Bulinus truncatus* snails as intermediate hosts and *Mesocricetus auratus* as definitive hosts. The parasite strains originate from Cameroon and Spain for *S. haematobium* and *S. bovis*, respectively [47]. The *S. haematobium* strain was initially recovered from the urine of infected patients during summer 2015 (Barombi Kotto lake; 4°28'04"N, 9°15'02"W). Eggs from positive samples were hatched, *miracidia* were harvested, and sympatric *B. truncatus* molluscs were individually exposed to five *miracidia* before being transferred to the IHPE laboratory for parasite maintenance. The *S. bovis*, strain isolated in the early 80s [47,48] was kindly provided by Ana Oleaga from the Spanish laboratory of parasitology of the Institute of Natural Resources and Agrobiology in Salamanca, and originates from Villar de la Yegua-Salamanca.

Experimental infections

Protocols of experimental infections were set for two objectives, i) quantifying the frequency of hetero-specific and homo-specific pairings and, ii) forcing hybridization and then assessing the transcriptomic changes between homo-specific and hetero-specific paired males and females. The successive steps of our experimental infection procedure is presented in Figure 1. Detailed procedures for mollusc and rodent infections have been previously described [49–51]. Step 1: 3-5 mm *B. truncatus* were individualized in 24-well plates containing 1ml of spring water per well. Each mollusc was exposed overnight to a single *miracidium* (*i.e.*, a single male or female genotype) of either *S. haematobium* or *S. bovis.* The following morning molluscs were placed in breeding tanks and fed *ad libitum* for the duration of the experiment. After a minimum period of 55 days, corresponding to the development time of the parasites in their intermediate host, molluscs were stimulated under light for *cercariae* shedding. Step 2: cercariae from each infected mollusc were recovered for molecular sexing using procedure previously described [47]. Step 3: molluscs were gathered into four distinct tanks according to the species and the sex of the infecting parasite. Step 4 : hamsters were individually exposed to *cercariae* by surface application method for one hour [49–51]. The sex and the species of the *cercariae* used for each experiment are presented in Table 1 and are described further (*Mating experiments analysis, Forced pairing).*

Fig 1. **Schematic representation of experimental infection procedure.**

Table 1: **Number of cercariae used for each experiment according to the species and the sex of the parasite.**

**Experiments**	***S. haematobium***		***S. bovis***		**Number of hamsters**
	**Males**	**Females**	**Males**	**Females**	
**Objective 1:** Quantification of homo- and hetero-specific pairs frequency					

**Limited Choice Experiment**					
Exp. 1 (*S. haematobium* males' choice)	150	225	-	225	5
Exp. 2 (*S. haematobium* females' choice)	225	150	225	-	5
Exp. 3 (*S. bovis* males' choice)	-	225	150	225	5
Exp. 4 (*S. bovis* females' choice)	225	-	225	150	5
**Full Choice Experiment**					
Exp. 5	150	150	150	150	5
**Objective 2:** Assess the transcriptomic profiles of homo- and hetero-specific paired worms					
Homo-specific forced pairing 1	300	300	-	-	6
Homo-specific forced pairing 2	-	-	300	300	6
Hetero-specific forced pairing 1	300	-	-	300	6
Hetero-specific forced pairing 2	-	300	300	-	6

Hamsters were euthanized at three months after cercarial exposition and adult worms were recovered by hepatic perfusion technique [51]. Hamsters were autopsied and specific organs such mesenteric and portal veins were carefully checked to identify potential remaining worms. We recorded each worm's sex inferred by their strong sexual dimorphism [11] and their paring status (paired or single). Paired worms were manually separated under a light microscope. All worms collected were individualized in 96-well plates and were subjected to DNA extraction using the method described previously in Beltran et al. (2008) [52]. The species of each worm was identified using the rapid diagnostic procedure based on multiplex PCR reaction described by Webster and colleagues [53,54].

Mate choice analysis

Experimental design

The experimental procedure to quantify the frequency of homo- and hetero-specific pairs between *S. bovis* and *S. haematobium* consisted in five experiments (*i.e.*, Exp. 1 to Exp. 5, see Table 1). The first four experiments aimed to test individually the choice of each species and

sex (Exp. 1 and Exp. 3 for male choice - Exp. 2 and Exp. 4 for female choice for *S. haematobium* and *S. bovis*, respectively). In each experiment, five hamsters (used as biological replicates) were infected with mixed combinations of cercariae (Table 1). These four experiments represented a limited choice of mate where excess of one sex (of both species competing for mating) ensuring that all individuals of the other sex (that had the choice for homo- or hetero-specific pairings) will be mated (Table 1). Finally, the last experiment (Exp. 5, Table 1) represented full choice of mate. Hamsters were infected with equal numbers of cercariae of both sexes and both species so that all combination of mating can be assessed at the same time.

Statistical analysis

After counting the total number of adult worms recovered for each species (*e.g.*, homo-specific pairs, hetero-specific pairs and single worms), we calculated the expected number of single and paired worms according to the null hypothesis of random pairing (*e.g.*, in the Exp. 1, the expected number of homo-specifically paired *S. haematobium* males equals the total number of *S. haematobium* males times the total number of *S. haematobium* females over the total number of females). Expected and observed numbers of homo- and hetero-specific pairs were then compared using Chi-square tests.

Forced pairing

Experimental design

Hamsters were infected with four combinations of parasites (Table 1). Homo-specific pairing consisted in hamster infections with single species of cercariae while hetero-specific pairing consisted in hamster infections with male and female cercariae of the opposite species. Hamsters were euthanized at three months after cercarial exposition and adult worms were recovered by hepatic perfusion technique. Paired worms were separated under binocular

magnifier and pooled according to their sex (male or female) and the species of their sexual partner (same or opposite species). Pools of 10-12 female or male worms were placed in 2ml microtubes and immediately frozen in liquid nitrogen and stored at -80°C. Three biological replicates were constituted for each combination representing a total of 24 samples (2 sexes x 4 combinations x 3 replicates) for subsequent RNA extraction and transcriptome sequencing (see Figure 2 for a schematic view of the procedure).

Fig 2. Schematic representation of the procedure used to obtain the reciprocal homo- and hetero-specific pairs of *S. haematobium* and *S. bovis.* *S. h* = *S. haematobium* and *S. b* = *S. bovis.*

RNA extraction and transcriptome sequencing of homo- and hetero-specific* S. haematobium and S. bovis *male and female pairs

Trizol RNA extraction and subsequent paired-end Illumina HiSeq 4000 PE100 sequencing technology was performed on the 24 samples. Briefly, pools of adult worms were ground with two steel balls using Retsch MM400 cryobrush (2 pulses at 300Hz for 15s). Total RNA was extracted using the Trizol Thermo Fisher Scientific protocol (ref: 15596018) slightly modified as the volume of each reagent was halved. Total RNA was eluted in 44 µl of ultrapure water before undergoing a DNase treatment using Thermofisher Scientific Turbo DNA-free kit. RNA was then purified using the Qiagen RNeasy mini kit and eluted in 42µl of ultrapure water. Quality and concentration of the RNA was assessed by spectrophotometry with the Agilent 2100 Bioanalyzer system and using the Agilent RNA 6000 nano kit. Further details are available at Environmental and Evolutionary Epigenetics Webpage (http://methdb.univ-perp.fr/epievo).

Illumina library construction and high-throughput sequencing

cDNA library construction and sequencing were performed at the Génome Québec platform. The TruSeq stranded mRNA library construction kit (Illumina Inc., USA) was used following the manufacturer's protocol on 300 ng of total RNA per sample. Sequencing of the 24 samples was performed in 2x100 bp paired-end on a Illumina HiSeq 4000 (Table S1). Sequencing data are available at the NCBI-SRA under the BioProject PRJNA491632.

Transcriptomic analysis of hetero-specific pairing* versus *homo-specific pairing effect

Raw sequencing reads were analysed on the Galaxy instance of the IHPE laboratory [55,56] First, raw reads were subjected to quality assessment and sequence adaptor trimming. We used the set of tools based on FASTX-toolkit [57], as well as Cutadapt program (Galaxy Version 1.16.1) to remove adapter sequences from Fastq files [58]. Finally, paired end reads were joined in a single fastq file using the FASTQ interlacer/de-interlace programs (Galaxy Version 1.1). Processed reads were mapped using RNA-star Galaxy Version 2.6.0b-1 [59] to the *S. haematobium* reference genome [60] downloaded from the Schistosoma Genomic Resources website SchistoDB (http://schistodb.net/common/downloads/Current_Release/ShaematobiumEgypt/fasta/data/). Exon-intron structure was thereafter reconstructed for each mapping BAM file using Cufflinks transcript assembly Galaxy Version 2.2.1.2, by setting the max intron length at 50000, but without any correction parameters [61]. Finally, in order to create a reference transcriptome representative of *S. haematobium* and *S. bovis* male and female reads, we merged all cufflinks data with Cuffmerge Galaxy Version 2.2.1.2 [61] without using any guide or reference. This enabled us to create a representative reference transcriptome of both species and both sexes using the same reference genome. The Genomic DNA intervals of all newly assembled genes

of this reference transcriptome were extracted from the *S. haematobium* reference genome and converted into a Fasta file.

The number of reads per transcript for each sample (*i.e.*, the read abundance representative of each gene) was quantified using HTseq-count Galaxy Version 0.9.1 on the reference transcriptome, setting the overlap resolution mode on "union" [62]. Finally, we evaluated the differential gene expression levels between homo-specifically and hetero-specifically paired worms for each species and each sex separately using DESeq2 Version 1.28.1 [63] run on R version 4.0.0 [64]. We carried out four types of comparisons which respectively focused on *S. haematobium* males, *S. haematobium* females, *S. bovis* males and *S. bovis* females and contrasted gene expression profiles between hetero-specifically paired individuals and homo-specifically paired individuals. Differential gene expression results were filtered on the adjusted *P-value* (Benjamini-Hochberg multiple testing based False Discovery Rate (FDR)) and considered significant when ≤ 5%.

Functional annotation

Using our BLAST local server, we annotated the entire *de novo* assembled transcriptome by Blastx search against the non-redundant database of the NCBI. We conserved only the longest unique transcript (TCONS) of each representative gene (XLOC) for Blastx search and subsequent analysis. Output XML files were used for gene ontologies (GO) mapping and annotation using Blast2Go version 4.1.9 [65]. Finally, enrichment Fisher's exact tests were performed on up and down regulated sets of genes focusing on biological process (BP) ontology terms. The *P-value* for significance was set to 5% False Discovery Rate (FDR).

Results

Mating choice experiments

Limited choice: Experiments 1 to 4

Details on the number of worms recovered from each hamster and whether they were paired or single are summarized in Table 2. For each mate choice experiment both homo-specific and hetero-specific pairs were observed (Table 2, Exp. 1–4). Also, in each limited choice of mate experiment (Exp. 1–4) we consistently obtained an excess of single worms of both species competing for pairing (*i.e.*, male or female depending on the experiment) whereas all choosing worms were paired (Table 2). This indicates that the choosing partners in each experiment were not limited in their choice by the number of potential homo- or hetero-specific partners. Specifically, in the experiment 1, the number of homo- and hetero-specific pairs of male *S. haematobium* was significantly different from those expected under the random mating hypothesis (χ2=11.10; d.f.=4; P-value= 0.049, Table 2). This was due to the deviation from the random mating hypothesis in one hamster (hamster number 3, see Table 2). Regarding *S. haematobium* females' choice (Table 2, Exp. 2) at the contrary, the numbers of homo-specific pairs and hetero-specific pairs were not significantly different from expectations under the random mating hypothesis (χ2=3.118; d.f.=4; P-value=0.682, Table 2). In the experiment 3 that focused on *S. bovis* males' choice, the total number of paired worms recovered was extremely low, due to premature death of two hamsters and only two hamsters had enough worms to be analysed (Table 2, Exp. 3). Although in this case, statistics should be interpreted with caution, the numbers of homo-specific and hetero-specific pairs were once again not significantly different from expectations under a random mating hypothesis (χ2=4.522; d.f.=1; P-value=0.104, Table 3). Finally, regarding *S. bovis* females' choice (Table 2, Exp. 4) similarly we did not find a significant difference between the numbers of observed and expected homo-

specific pairs and hetero-specific pairs under random mating hypothesis (χ2=3.246; d.f.=4; P-value=0.662, Table 2). Overall, when analysing all limited choice experiments together (*i.e.*, Exp. 1 to 4) no significant difference was recorded between the number of observed homo- and hetero-specific pairs and those expected under a random mating scenario (χ2=21.71, d.f.=16, P-value=0.152, Table 2).

Table 2. Summarized information of experiments one to four (limited choice). For each experiment are displayed the sex and the species of the choosing partner, the number of observed homo- and hetero-specific pairs and the number of worms that remained single. Sh = *S. haematobium* and Sb = *S. bovis*. Expected number of pairs under random mating hypothesis is shown in brackets (see the statistics section in methods for details). Chi-square statistic, degree of freedom and P-value are given for each hamster, for each experiment and for all experiments combined. * indicates significant results at 5% level. In Exp. 3, worms from only two hamsters could be analysed, other died prematurely (two hamsters) or presented too few numbers of paired worm (one hamster).

**Exp.**	**Host**	**Choosing partner**	**Homo-specific pairs**	**Hetero-specific pairs**	**Homo-specific single**	**Hetero-specific single**	**$\chi2$-statistic**	**d.f.**	**P-value**
			**♂ Sh x♀ Sh**	**♂ Sh x♀ Sb**	**♀ Sh**	**♀ Sb**			
1	1	♂ **Sh**	11 (14)	14 (11)	20	9	1.838	1	0.175
1	2	♂ **Sh**	11 (15)	22 (18)	15	11	1.543	1	0.214
1	3	♂ **Sh**	14 (20)	16 (10)	25	4	5.057	1	0.025*
1	4	♂ **Sh**	9 (9)	6 (6)	36	26	0.015	1	0.903
1	5	♂ **Sh**	10 (13)	10 (7)	41	15	2.651	1	0.103
**Exp. 1**							**11.104**	**4**	**0.049***
			**♀ Sh x ♂ Sh**	**♀ Sh x ♂ Sb**	**♂ Sh**	**♂ Sb**			
2	1	♀ **Sh**	10 (9)	2 (4)	7	5	0.908	1	0.341
2	2	♀ **Sh**	6 (5)	1 (2)	0	1	0.429	1	0.513
2	3	♀ **Sh**	12 (10)	3 (5)	11	10	1.688	1	0.194
2	4	♀ **Sh**	16 (15)	3 (4)	6	2	0.094	1	0.759
2	5	♀ **Sh**	12 (12)	13 (13)	11	12	0.001	1	0.993
**Exp. 2**			**♂ Sb x♀ Sb**	**♂ Sb x♀ Sh**	**♀ Sb**	**♀ Sh**	**3.118**	**4**	**0.682**
3	2	♂ **Sb**	4 (2)	0 (2)	46	55	4.400	1	0.036
3	3	♂ **Sb**	2 (1)	1 (2)	22	32	0.742	1	0.389
**Exp. 3**							**4.522**	**1**	**0.104**

			♀ **Sb x** ♂ **Sb**	♀ **Sb x** ♂ **Sh**	♂ **Sb**	♂ **Sh**			
4	1	♀ **Sb**	15 (12)	17 (20)	4	14	1.070	1	0.301
4	2	♀ **Sb**	10 (8)	25 (28)	2	19	1.061	1	0.303
4	3	♀ **Sb**	3 (3)	8 (8)	4	13	0.030	1	0.862
4	4	♀ **Sb**	9 (8)	15 (16)	8	19	0.188	1	0.665
4	5	♀ **Sb**	49 (49)	15 (15)	18	5	0.007	1	0.932
**Exp. 4**							**3.246**	**4**	**0.662**
**All Exp.**							**21.719**	**16**	**0.152**

Full choice: Experiment 5

Details on the number of worms recovered from each hamster and whether they were paired or single are summarized in Table 3. When all mating combinations were allowed between *S. haematobium* and *S. bovis*, four types of pairing combination were obtained: two being homo-specific (♂ Sh x ♀ Sh and ♂ Sb x ♀ Sb, Table 3) and two being hetero-specific (♂ Sh x ♀ Sb and ♂ Sb x ♀ Sh, Table 3). There was also an excess of males and females of both species remaining single, suggesting that all possible pairings were not limited by partner availability (Table 3). Regarding the number of homo-specific and hetero-specific pairs observed between *S. haematobium* and *S. bovis*, Chi-square tests did not reveal significant departure from random mating hypothesis, when the number of each pairing combination was analysed in each hamster separately and also when analysing all replicate together (Table 3).

Table 3. Summarized information of experiment 5 (full choice). For each combination (*i.e.*, sex and species) are given the number of observed pairs and the number of single partners that remained single. Sh = *S. haematobium* and Sb = *S. bovis*. Expected number of pairs under random mating is shown in brackets (see the statistics section in methods for details). Chi squared statistics, degree of freedom and P-value are given per hamster and for the whole experiment.

**Host no.**	**♂*Sh* x ♀ Sh**	**♂Sb x ♀ Sb**	**♂Sh x ♀ Sb**	**♂Sb x ♀ Sh**	**♂ Sh**	**♂ Sb**	**♀ Sh**	**♀ Sb**	**χ2-statistic**	**d.f.**	**P-value**
1	1 (2)	(24)	5 (9)	4 (5)	8	5	4	9	3.358	3	0.340
2	8 (8)	2 (1)	5 (4)	1 (2)	8	3	23	8	1.786	3	0.618
3	7 (5)	2 (2)	1 (2)	2 (3)	6	5	19	11	2.307	3	0.511
4	4 (4)	6 (3)	3 (4)	1 (3)	10	4	13	11	4.806	3	0.187
5	5 (6)	1 (1)	6 (6)	2 (1)	20	3	17	16	0.796	3	0.850
**Total**									**13.053**	**12**	**0.365**

Transcriptomic response in homo- vs. hetero-specific pairs

RNA sequencing, transcriptome assembly and gene annotation of the homo- and hetero-specific pairs

We analyzed separately 24 samples, corresponding to biological triplicates of males and females of the four forced pairing combinations described in Table 1 and Figure 2 (*i.e.*, homo- and hetero-specifically paired males and females). Between ~24.7 and ~42.3 million high quality Illumina HiSeq 4000 PE100 RNA-seq reads were obtained after sequencing of the 24 samples. After quality control and adaptor trimming, between ~19.2 and ~33,1 million reads were uniquely mapped to the *S. haematobium* reference genome and used for gene expression analysis [60]. On average ~78% of raw reads were mapped to the reference genome, with 51% of which corresponded to *S. haematobium* and 49% to *S. bovis* (Supplementary Table S1).

The reference transcriptome assembly on which tests were carried out, was composed of 73,171 putative isoform sequences identified as TCONS, and 18,648 unique genes identified as XLOCS. We conserved the longest isoform (TCONS) for each gene (XLOC) for subsequent annotation. The GTF and Fasta file of this transcriptome are available in a Figshare repository ("https://figshare.com/s/2f299e6f53c94f4a6168"). Blast annotations and Gene Ontology terms of the complete reference transcriptome are available in Supplementary File S1, Sheet 1. On the 18,648 genes, 14,414 found at least one hit following Blastx analysis, and 12,332 of them were mapped to at least one GO term using Blast2GO [65].

Differential gene expression

Quantification of read abundance as well as differential gene expression analysis were performed on the 18,648 genes for each homo- and hetero-specific conditions (Supplementary File S1, Sheet 2 and 3-6, respectively). The heatmap of the sample-to-sample distances as well as the principal component analysis plot are presented in Fig 3. A total of 1,277 genes (~7% of

the 18,648 genes present in the reference transcriptome) were differentially expressed in at least one of the four homo- versus hetero-specific comparisons with a FDR <5% (Supplementary File S1, Sheet7). Of these, 1,234 (97%) had a match using Blastx against the non-redundant database of the NCBI and 1,088 (85%) were mapped and successfully annotated with at least one GO term using Blast2GO [65] (Supplementary File S1 Sheet 7).

Fig 3. Differential gene expression profiles. a) Principal component plot of the samples and b) Heatmap of the sample-to-sample distances.

Most of the differentially expressed genes (DEGs) were identified in *S. haematobium* males, with 1,166 DEGs between the hetero-specific and homo-specific pairing combinations (734 over-expressed and 432 under-expressed in hetero-specific paired males compared to homo-specific ones). Log2-Fold changes were quite low with only one of these 1,166 DEGs having a Log2-Fold Change higher than 1.5 and none had Log2-Fold change lower than -1.5 (Fig 4, Supplementary File S1 Sheet 7). In *S. haematobium* females, 47 genes were differentially expressed between hetero- vs. homo-specific conditions (22 over-expressed and 25 under-expressed in hetero-specific females). Among these 47 DEGs, six had Log2-Fold changes higher than 1.5 and one had a Log2-Fold change lower than -1.5 (Fig 4, Supplementary File S1 Sheet 7). In *S. bovis* females, 88 genes were differentially expressed between hetero- vs. homo-specific conditions (58 over-expressed and 30 under-expressed in hetero-specific females). Among these 88 DEGs, 48 had Log2-Fold changes higher than 1.5 and 11 had Log2-Fold changes lower than -1.5 (Fig 4, Supplementary File S1 Sheet 7). Finally, no DEGs were identified in *S. bovis* males. Significantly (*p*<5%) over- and under-expressed genes (XLOC) for each comparison as well as their annotation are shown in Supplementary File S1, Sheet 7.

Fig 4. Genes expression profiles in hetero-specifically compared to homo-specifically paired worms. Volcano plots showing the log transformed adjusted P-values (*i.e.,* FDR) and the log fold changes for the 18,648 unique genes of the reference transcriptome assembly for *S. haematobium* males *a*), *S. haematobium* females b), *S. bovis* females c) and *S. bovis* males d). Black dots refer to non-significant genes regarding their expression profile (over an FDR of 5%). Red dots refer to differentially expressed genes at a FDR of 5%, green dots refer to differentially expressed genes at a FDR between 5% and 1% and blue dots refer to DEGs at a FDR between 1% and 1 ‰.

Gene Ontology and enrichment analysis of the differentially expressed genes

Gene ontology categories significantly enriched in either over- or under-expressed genes were found in *S. haematobium* males (Fig 5**,** Supplementary File S1, Sheet 8) whereas in *S. haematobium* females, *S. bovis* males and females (in which fewer DEG were detected), no GO terms were significantly enriched.

Fig 5. Biological processes impacted by hetero-specific pairing in male *S. haematobium.* Barplot showing the biological processes significantly enriched in DEGs (at a FDR threshold of 5%), either over-expressed or under-expressed in hetero-specific condition compared to homo-specific condition, in *S. haematobium* males.

In *S. haematobium* males, biological processes enriched in under-expressed genes (in hetero-specific paired males compared to homo-specific ones) were related to signal transduction, notably through neuronal processes (synaptic transmission, cholinergic, chemical synaptic transmission, postsynaptic, G protein−coupled receptor signalling pathway), development (anatomical structure development), metabolism (glycogen biosynthetic process, negative regulation of endopeptidase activity), transmembrane transport (potassium ion transmembrane transport), response to stimuli (response to drug, peptidyl−proline hydroxylation, cell redox

homeostasis) and cell adhesion (homophilic cell adhesion via plasma membrane adhesion molecules) (Fig 5**,** Supplementary File S1, Sheet 8).

On the other hand, biological processes enriched in over-expressed genes (in hetero-specific males) were related to signal transduction including again some neuronal processes (*e.g.*, transmembrane receptor protein tyrosine kinase signalling pathway, regulation of Ras protein signal transduction, regulation of axon extension), metabolism (*e.g.*, proteolysis involved in cellular protein catabolic process, phosphatidylcholine metabolic process, long‒chain fatty acid metabolic process, lipid droplet organization), response to stimuli (*e.g.*, response to other organism, phagocytosis, cellular response to chemical stimulus), transmembrane transport (*e.g.*, anion transmembrane transport, vesicle fusion, regulation of vesicle‒mediated transport, inorganic cation import across plasma membrane, exocytosis, positive regulation of Notch signaling pathway), localization (*e.g.*, establishment of localization in cell), locomotion (*e.g.*, regulation of locomotion, microtubule‒based process, actin filament organization) and also cell adhesion (*e.g.*, cell junction assembly) (Fig 5**,** Supplementary File S1, Sheet 8).

No GO terms were found enriched neither in over- nor under-expressed genes in *S. bovis* and *haematobium* hetero- vs. homo-specifically paired females (Supplementary file S1, Sheet 7). However, based on annotations, in *S. haematobium* females, we found differentially expressed genes that corresponded to genetic mobile elements (*e.g.*, XLOC_014282: integrase core domain, XLOC_014741: TPA: endonuclease-reverse transcriptase, XLOC_009783: endonuclease-reverse transcriptase), genes involved in transmembrane transport (*e.g.*, XLOC_009318: phosphatase methylesterase 1 (S33 family) and XLOC_010891: Calcium-binding mitochondrial carrier S -1), stress response including oxidation-reduction processes (*e.g.*, XLOC_017856: heat shock, XLOC_012518: epidermal retil dehydrogese 2 and XLOC_018492: iron-dependent peroxidase) and other functions such as reproduction, or development (*e.g.*, XLOC_015776: egg CP391S, XLOC_007823: Craniofacial development

2). Similarly, in *S. bovis* females, we found differentially expressed genes that correspond to genetic mobile elements as well (*e.g.*, XLOC_017328: R-directed D polymerase from transposon X-element, XLOC_018050: R-directed D polymerase from mobile element jockey-like or XLOC_018156: gag-pol poly), genes involved in ion transport (*e.g.*, XLOC_008268: Bile salt export pump, XLOC_003851: sodium-coupled neutral amino acid transporter 9 isoform X2 and XLOC_005754: Y+L amino acid transporter), response to stress (*e.g.*, XLOC_017856: heat shock and XLOC_009339: Universal stress) and as well other functions such as reproduction, growth or metabolism (*e.g.*, XLOC_014939: early growth response, XLOC_015393: Syptotagmin-1, XLOC_016728: egg CP391S-like and XLOC_012591: Cathepsin B-like cysteine proteinase precursor). Hence, for *S. haematobium* and *S. bovis* females, DEGs were quite similar in term of function, regardless of their expression profile (under- or over-expression in hetero-specific pairs) and regardless of the schistosome species.

Discussion

In this study we aimed to investigate potential reproductive isolation mechanisms between two major African schistosome species that cause major debilitating parasitic disease and show evidences for extensive hybridization in nature [35,66]. Specifically, we tested whether hybridization between *S. haematobium* and *S. bovis* could be constrained or promoted by mate choices and whether these mate choices were associated with specific transcriptomic profiles in hetero- and homo-specifically paired individuals. Overall, the data shows that *S. haematobium* and *S. bovis* mate in a random fashion and seems to depend only on the presence and the relative abundance of each species in the definitive host. Likewise, we did not detect any major transcriptomic changes associated with hetero-specific pairing in male and female *S. haematobium* and *S. bovis*.

First, in this work, we showed that the two frequently co-endemic sister species *S. haematobium* and *S. bovis* readily pair with no preferences for neither homo-specific nor hetero-specific associations in simultaneous infections. The only exception was found for male *S. haematobium* mate choice. Indeed, we found a significantly higher number of hetero-specific pairs compared to that expected under the assumption of random mating. However, as we cannot differentiate mate recognition initiated by males from female competition, our results suggest that either male *S. haematobium* prefer mating with female *S. bovis* or alternatively, that female *S. bovis* may be more competitive than female *S. haematobium*. Interestingly, although female competition is possible, it is assumed that male schistosomes are the competitive sex and in particular male *S. haematobium* are usually better at pairing when compared to males from other species including *S. intercalatum* [33], S. *mattheei* [29] or *S. mansoni* [28]. However, since the bias toward hetero-specific pairing was mainly due to one hamster, this result should be considered with caution. Indeed, this bias was not retrieved in our full mate choice experiments and future studies are warranted to confirm if this observation is repeatable as it may have important epidemiological consequences for the direction in pairing and hybrid representation in the field. Similarly, premature death of some hamsters in the experiment focusing on the mate choice of male *S. bovis* limited our ability to drawn specific conclusions. Consequently, our mate choice experiments overall rather indicate no differences in species mate choice or competitiveness and that *S. haematobium* and *S. bovis* males and females mate randomly. This highlights that there are no behavioural barriers preventing hetero-specific pairing once both species encounter each other in the same definitive host.

The second part of this study aimed to assess the transcriptomic profiles associated with hetero-specific pairings between *S haematobum* and *S. bovis*. Since different species might constitute a different stimulus for the other partner, we expected at first to find an impact of the hetero-specific pairing, and especially on female transcriptomes compared to males since they respond

to males' stimuli for their sexual maturation [20]. However, only few DEGs were observed in both males and females. Biological processes enriched in DEGs were identified only for male *S. haematobium* pairings. Likewise, most of the genes detected presented low Log2-Fold changes (notably in *S. haematobium* males where only one DEG exceeded a Log2-Fold change of 1.5). Thus, the influence of hetero-specific pairing on male and female adult worms of both species in terms of number of DEGs, related biological processes and gene expression level was not striking. Such results suggest that both species may be highly receptive to each other since no major transcriptomic adjustments are induced by hetero-specific pairings. This observation is hence consistent with our previous mating experiments that suggest random pairing between both species and further show that there are no major physiological nor molecular barriers making hetero-specific pairings and thus hybridization less prone to occur.

Although hetero-specific pairings did not result in many DEGs, it is worth noting that most of the DEGs were found in the comparison between homo- and hetero-specifically paired male *S. haematobium*. So far transcriptomic studies on *Schistosoma* pairing tended to show large molecular reprograming of female genes rather than male genes, in part due to the initiation of their sexual maturation [13,45,46]. The biological explanations for our results are thus not straightforward. First, we cannot rule out the possibility of an artefact induced by extrinsic factors or other technical issues such as a lower variability in the transcriptomic profiles of the different biological replicates of male *S. haematobium* in comparison to other samples. However, our results also show that male *S. haematobium* displayed more DEGs than females but DEG identified in females presented overall higher log2 Fold Changes. Hence, another hypothesis could be that females may differentially express fewer genes, but at higher levels. Finally, we could also hypothesize that the molecular plasticity in expression of genes is a mechanism by which male *S. haematobium* manage to be more competitive (compared to females from both species and *S. bovis* males) in hetero-specific pairing, for instance by

properly initiating female maturation depending on their species. This latter hypothesis is particularly appealing since male *S. haematobium* are thought to be dominant over several other *Schistosoma* species [28,29,33]. This is also congruent with the potential bias toward hetero-specific pairing of *S. haematobium* males we documented in our mating experiments and with field studies that recorded high frequencies of hybrids between male *S. haematobium* and female *S. bovis* [35,67]. Nevertheless, since we did not identify any DEG in *S. bovis* males, and also because the log2-Fold change of the DEG identified in *S. haematobium* males were low, it seems difficult to conclude that one or the other sex is preferentially impacted during hetero-specific pairing, or that one species is more prone to initiate the sexual maturation of females. However, we are confident that the small number of DEGs identified when comparing homo- and hetero-specific parings together with their low log2 Fold Change reflect the relatedness between *S. bovis* and *S. haematobium* that undergo only few transcriptomic adjustments following hetero-specific pairing. Moreover, the molecular changes that we identified here at the very first step in the hybridization process may reveal some important genes linked to male and female interactions, species isolation and hybridization.

Indeed, some of the DEGs identified in our work show functions that can be linked to sex interaction, notably to reproductive functions suggested by other studies. Notably, among female schistosomes we found three genes encoding egg proteins that were differentially expressed in *S. haematobium* and/or *S. bovis* females, and that are well-known female-associated gene products [68]. Similarly, a transcript matching the Syptotagmin-1 gene was under-expressed in hetero-specifically paired female *S. bovis*. This gene was previously shown to have a female-specific expression and to be regulated during pairing [45]. Moreover, two DEGs that encode digestive enzymes, specifically expressed by paired females (*i.e.*, cathepsin B and L) were found in female *S. bovis* [68]. Similarly, in *S. haematobium* DEGs were related to biological processes known to be involved in male-female interactions. Previous studies

looking at the molecular basis of schistosomes gender interplay with a particular interest in the pairing process, proliferation, differentiation and maturation of female gonads have underlined the major role of signal transduction cascades and particularly signalling pathways such as the TGF-beta and Ras (*e.g.*, receptor tyrosine kinase coupled pathway) signalling pathways [69–75]. These pathways, notably the TGF-beta signaling pathway are known to induce the production of the gynecophoric canal protein by males during pairing which is a trigger for maturation of females [70]. Interestingly, in this work, among genes whose expression was affected by homo- and hetero-specific pairing in male *S. haematobium*, we notably found the TGF-beta signal transducer gene, and two gynecophoral canal protein genes. Moreover, both transmembrane receptor protein tyrosine kinase signaling pathway and regulation of Ras protein signal transduction processes were enriched in over-expressed genes in hetero-specific pairs. Also echoing more recent studies on the gonad-specific and pairing-dependent transcriptomes of male schistosomes, we found several biological processes enriched either in over- or under-expressed genes in *S. haematobium* males that were involved in neuronal processes which are associated with male-female interaction patterns [13,46]. We consequently found that genes and processes impacted between homo- and hetero-specific pairing in *S. haematobium* and *S. bovis* at least partly overlapped those generally affected in other male-female interaction studies. These results suggest that both species may have maintained similar patterns of interactions between males and females allowing them to reproduce. A moderate regulation of these genes during pairing with another species may thus allow the two parasite species to overcome their relatedness and divergence resulting in successful hetero-specific mating. Finally, it is worth noting that among the DEGs identified, the majority of them were also related to processes that were not particularly documented to be impacted during male-female interactions (*e.g.*, genetic mobile elements, response to drug and stimuli, oxidation-reduction). Several DEGs were related to stress response and stimuli responses (*e.g.*, oxidation-

reduction processes as well as the genetic mobile elements [76,77]), indicating that at least at the molecular level schistosome species may perceive hetero-specific pairing as a stress, although this does not seem to impede hetero-specific pairing. Alternatively, the pairing status (*i.e.*, homo-specific or hetero-specific) could impact the worms' responses to external stimuli including host and/or environmental stimuli. In particular hetero-specific male *S. haematobium* under-expressed a fair amount of genes involved in response to drugs compared to homo-specific ones (*e.g.*, Multidrug and toxin extrusion, Multidrug and toxin extrusion 2, Multidrug resistance or Multidrug resistance-associated). These observations may raise important questions regarding schistosomes' drug response in the context of co-infection and hybridization especially since a lower sensitivity to PZQ of *S. bovis* x *S. haematobium* hybrids compared to pure *S. haematobium* parasites has been proposed to be at the origin of the spread of the hybrid form in Senegal [26,27]. However, we found a differential expression of genes involved in response to drugs in male *S. haematobium* only, which call for future clarification to assess if this is a peculiarity of our study and/or of male *S. haematobium*. More generally, several other genes identified in this work may be of potential significance for the encounter, interaction, and communication between these two species. Further attention is thus required to decipher the role of each of them in the context of hybridization or at the contrary in the context of speciation.

Altogether, the integrative assessment of lack of pre-zygotic reproductive mechanisms we present here may have profound implications regarding what we could expect in term of hybridization dynamics in the field. In particular it suggests that both species have retained similar processes allowing them to find their partner in the host, pair and produce viable offspring. This result is in line with several recent studies that have presented evidences of introgression between *S. haematobium* and *S. bovis* and that suggest that their relatively recent

divergence compared to other schistosomes and thus the genetic distance between both species is not sufficient to limit hybridization [37,38,78]. This relies in part to the fact that they have retained the same karyotype with n=8 chromosome pairs, including sex chromosomes that are morphologically similar [79], hence allowing the species' genomes to be highly permeable to each other's alleles [80]. In that case, the most significant reproductive isolation mechanisms preserving the genetic integrity between these species would be habitat isolation, including geographical location and definitive host specificity. Also, it is worth noting however that the two species used in this study have been isolated from distinct geographical zones and this could contribute to explain the absence of pre-zygotic isolation. Indeed, sympatric African schistosome species are likely to respond differentially as sympatric species tend to have enhanced pre-zygotic isolation barriers [4]. Nevertheless, the lack of pre-zygotic barriers does imply that in areas where *S. haematobium* and *S. bovis* are sympatric and infect the same definitive hosts, hybrids and introgressed individuals should be more likely to be found. This may be particularly relevant for parasite species that are brought together by global changes (enhanced human migration for *S. haematobium,* and animal transhumance for *S. bovis)* and may have porous reproductive isolation mechanisms.

While our study opens new avenues regarding the understanding of the mechanisms allowing or preventing hybridization between schistosome species, it also calls for future experimental and field work to fully understand hybridization patterns observed *in natura*. First, our observation of random mating between the two species suggests that first-generation hybrids may be frequent in endemic areas. A recent study in Senegal found hybrids with mixed genetic profiles between parental species suggesting that they may be of early generation [43]. However, current genomic analyses of parasites recovered in the field indicate that introgression between *S. haematobium* and *S. bovis* is the result of an ancient event rather than an ongoing process [38,78,81]. This is also supported by the genetic differentiation between

hybrids and parental species populations in Senegal and Niger [82,83]. Second, although a broader view of the hybridization dynamics is warranted by increasing the number of samples collected across the African continent, the current data suggests that at least in the field *S. haematobium* could be dominant over *S. bovis* and that hybridization patterns may differ between foci. Indeed, several studies report unidirectional introgression of *S. bovis* genes into *S. haematobium* [34,38] and a predominance for an initial cross between a male *S. haematobium* and a female *S. bovis,* (leading to the introgression of mitochondrial DNA of the latter in the genomic background of the former [26,35]). Such biases in the direction of the crosses and introgression patterns are frequent in the hybridization landscape including for schistosome species. For instance while some species hybridize in both directions and over multiple generations (*S. bovis* and *S. curassoni*; [27,34]; *S. mansoni* and *S. rodhaini*; [66]) others may produce offspring with strong asymmetries in their fitness (*S. haematobium* and *S. mattheei;*[84], *S. haematobium* and *S. intercalatum* [29]) and sometimes in the directionality of introgression (*S. rodhaini* and *S. mansoni* [28]. However, since our analysis of the pre-zygotic isolation mechanisms does not support any type of asymmetry in the direction of the crosses it is most likely that if any, post-zygotic barriers may be at the origin of such biased patterns in the field and the relatively rare encounter in early generation hybrids. Consequently, the genomic landscape of introgression and the transmission patterns of hybrids may not be uniform, are highly complex and potentially dynamic. In this context it would be necessary as a next step to assess the importance of post-zygotic isolation mechanisms in terms of snail compatibility, hybrid life history traits and potential heterosis, which are important biological features that may shape hybridization outcomes by potentially reducing or promoting inter-species interaction and admixture. This may have strong implications as hybridization in schistosomes is a major concern and since heterosis in offspring may increase the parasite virulence compared to their parental species [85,86]. Such changes in the parasites life history

traits may have important outcomes in terms of epidemiological dynamics (hybrids may take over parental species range [87], but also threaten the transmission, control and ultimate elimination of schistosomiaisis). In this context, a better understanding of the consequences of hybridization in parasites is a necessary next step to anticipate its effect in terms of disease dynamics and spread.

In conclusion, in this integrative study of *S. haematobium* and *S. bovis* behavioural and physiological isolation mechanisms we showed that natural hybridization between *S. haematobium* and *S. bovis* lack strong pre-zygotic barriers apart from the one presented by their host specificity. Our data suggest that no mate recognition system mitigates hybridization between these two species and that no major transcriptomic adjustments are associated with hetero-specific pairings. This highlights that the two species remain sufficiently coadapted to each other to allow an efficient reproduction once they are in contact. Besides the current evidence of ancient introgression and biases in hybrid profiles, this weak pre-zygotic barrier we exemplified raises the risk that in the absence of other reproduction isolation mechanisms, hybridization between these two species may be common. This also implies that contact zones may need further consideration to assess if hybridization is ongoing. Finally, our results may also partly explain the high prevalence of these hybrids in the field. Because such inter-species interaction may increase the offspring's virulence compared to parental species, one could expect to find increased prevalence and intensities of the disease in areas where hybridization occurs. Understanding the modifications in the parasite life history traits, including their zoonotic potential and epidemiological outcomes are warranted if we are willing to control human and animal morbidity, reduce transmission and ultimately eliminate schistosomiasis.

Acknowledgments

This work was supported by the program HySWARM (ANR-18-CE35-0001) from the French Research National Agency and the Occitania region (MOLRISK). We would like to acknowledge Génome Québec and University McGill innovation center as well as Roscoff Bioinformatics platform ABiMS (http://abims.sb-roscoff.fr) and NGS facility at the bio-environment platform (University of Perpignan). This study is set within the framework of the "Laboratoires d'Excellence (LABEX)" TULIP (ANR-10-LABX-41).

References

1. Barton N, Bengtsson BO. The barrier to genetic exchange between hybridising populations. Heredity. 1986;57: 357–376. doi:10.1038/hdy.1986.135
2. Nosil P. Ecological speciation. Oxford University Press; 2012.
3. Barnard-Kubow KB, Galloway LF. Variation in reproductive isolation across a species range. Ecol Evol. 2017;7: 9347–9357. doi:10.1002/ece3.3400
4. Coyne JA, Orr HA. "Patterns of Speciation in Drosophila" Revisited. Evolution. 1997;51: 295. doi:10.2307/2410984
5. Windsor DA. Controversies in parasitology, Most of the species on Earth are parasites. Int J Parasitol. 1998;28: 1939–1941. doi:https://doi.org/10.1016/S0020-7519(98)00153-2
6. Henrich T, Kalbe M. The role of prezygotic isolation mechanisms in the divergence of two parasite species. BMC Evol Biol. 2016;16: 1–10. doi:10.1186/s12862-016-0799-5
7. Ramiro RS, Khan SM, Franke-Fayard B, Janse CJ, Obbard DJ, Reece SE. Hybridization and pre-zygotic reproductive barriers in Plasmodium. Proc R Soc B Biol Sci. 2015;282. doi:10.1098/rspb.2014.3027
8. Léger E, Webster JP. Hybridizations within the Genus Schistosoma: implications for evolution, epidemiology and control. Parasitology. 2016; 1–16. doi:10.1017/S0031182016001190
9. Colley DG, Bustinduy AL, Secor WE, King CH. Human schistosomiasis. Lancet Lond Engl. 2014;383: 2253–2264. doi:10.1016/S0140-6736(13)61949-2
10. Loker ES, Brant S V. Diversification, dioecy and dimorphism in schistosomes. Trends Parasitol. 2006;22: 521–528. doi:10.1016/j.pt.2006.09.001
11. Basch PF. Why do schistosomes have separate sexes? Parasitol Today. 1990;6: 160–163. doi:10.1016/0169-4758(90)90339-6

12. Moné H, Boissier J. Sexual biology of schistosomes. Adv Parasitol. 2004;57: 89–189. doi:10.1016/S0065-308X(04)57002-1

13. Lu Z, Sessler F, Holroyd N, Hahnel S, Quack T, Berriman M, et al. Schistosome sex matters: A deep view into gonad-specific and pairing-dependent transcriptomes reveals a complex gender interplay. Sci Rep. 2016;6: 1–14. doi:10.1038/srep31150

14. Boissier J, Moné H. Experimental observations on the sex ratio of adult *Schistosoma mansoni*, with comments on the natural male bias. Parasitology. 2000;121: 379–383.

15. Beltran S, Boissier J. Male-biased sex ratio: why and what consequences for the genus *Schistosoma*? Trends Parasitol. 2010;26: 63–69. doi:10.1016/j.pt.2009.11.003

16. Beltran S, Boissier J. Schistosome monogamy: who, how, and why? Trends Parasitol. 2008;24: 386–391. doi:10.1016/j.pt.2008.05.009

17. Beltran S, Boissier J. Are schistosomes socially and genetically monogamous? Parasitol Res. 2009;104: 481–483. doi:10.1007/s00436-008-1225-8

18. Beltran S, Cézilly F, Boissier J. Genetic dissimilarity between mates, but not male heterozygosity, influences divorce in schistosomes. PLoS ONE. 2008;3. doi:10.1371/journal.pone.0003328

19. Popiel I. Male-stimulated female maturation inSchistosoma: A review. J Chem Ecol. 1986;12: 1745–1754. doi:10.1007/BF01022380

20. Popiel I, Cioli D, Erasmus DA. The morphology and reproductive status of female Schistosoma mansoni following separation from male worms. Int J Parasitol. 1984;14: 183–190. doi:https://doi.org/10.1016/0020-7519(84)90047-X

21. Clough ER. Morphology and reproductive organs and oogenesis in bisexual and unisexual transplants of mature *Schistosoma mansoni* females. J Parasitol. 1981;67: 535–539.

22. Cornford EM, Fitzpatrick AM. The mechanism and rate of glucose transfer from male to female schistosomes. Mol Biochem Parasitol. 1985;17: 131–141. doi:https://doi.org/10.1016/0166-6851(85)90012-X

23. Loverde PT, Chen L. Schistosome female reproductive development. Parasitol Today Pers Ed. 1991;7: 303–8. doi:10.1016/0169-4758(91)90263-N

24. LoVerde PT, Niles EG, Osman A, Wu W. *Schistosoma mansoni* male–female interactions. Can J Zool. 2004;82: 357–374. doi:10.1139/z03-217

25. Kunz W. Schistosome male-female interaction: induction of germ-cell differentiation. Trends Parasitol. 2001;17: 227–231. doi:10.1016/S1471-4922(01)01893-1

26. Huyse T, Webster BL, Geldof S, Stothard JR, Diaw OT, Polman K, et al. Bidirectional introgressive hybridization between a cattle and human schistosome species. PLoS Pathog. 2009;5. doi:10.1371/journal.ppat.1000571

27. Webster BL, Diaw OT, Seye MM, Webster JP, Rollinson D. Introgressive Hybridization of *Schistosoma haematobium* Group Species in Senegal: Species Barrier Break Down between Ruminant and Human Schistosomes. PLoS Negl Trop Dis. 2013;7. doi:10.1371/journal.pntd.0002110

28. Webster BL, Southgate VR, Tchuem Tchuenté L-A. Mating interactions between *Schistosoma haematobium* and *S. mansoni*. J Helminthol. 2007/02/01. 1999;73: 351–356. doi:DOI: 10.1017/S0022149X99000591

29. Webster BL, Southgate VR. Mating interactions of *Schistosoma haematobium* and *S. intercalatum* with their hybrid offspring. Parasitology. 2003;126: 327–338.

30. Southgate VR, Tchuenté LAT, Vercruysse J, Jourdane J. Mating behaviour in mixed infections ofSchistosoma haematobium and S. mattheei. Parasitol Res. 1995;81: 651–656.

31. Tchuente LAT, Imbert-Establet D, Delay B, Jourdane J. Choice of mate, a reproductive isolating mechanism between *Schistosoma intercalatum* and *S. mansoni* in mixed infections. Int J Parasitol. 1993;23: 179–185. doi:10.1016/0020-7519(93)90139-P

32. Cosgrove CL, Southgate VR. Mating interactions between *Schistosoma mansoni* and *S. margrebowiei*. Parasitology. 2002;125: 233–243.

33. Southgate VR, Rollinson D, Ross GC, Knowles RJ. Mating behaviour in mixed infections of *Schistosoma haematobium* and *S. intercalatum*. J Nat Hist. 1982;16: 491–496. doi:10.1080/00222938200770391

34. Brémond P, Sellin B, Sellin E, Naméoua B, Labbo R, Théron A, et al. Arguments for the modification of the genome (introgression) of the human parasite *Schistosoma haematobium* by genes from S. bovis, in Niger. C R Acad Sci III. 1993;316: 667–670.

35. Moné H, Holtfreter MC, Allienne JF, Mintsa-Nguéma R, Ibikounlé M, Boissier J, et al. Introgressive hybridizations of *Schistosoma haematobium* by *Schistosoma bovis* at the origin of the first case report of schistosomiasis in Corsica (France, Europe). Parasitol Res. 2015;114: 4127–4133. doi:10.1007/s00436-015-4643-4

36. Angora EK, Allienne J-F, Rey O, Menan H, Touré AO, Coulibaly JT, et al. High prevalence of *Schistosoma haematobium* × *Schistosoma bovis* hybrids in schoolchildren in Côte d'Ivoire. Parasitology. 2020;147: 287–294. doi:10.1017/S0031182019001549

37. Savassi BAES, Mouahid G, Lasica C, Mahaman S-DK, Garcia A, Courtin D, et al. Cattle as natural host for *Schistosoma haematobium* (Bilharz, 1852) Weinland, 1858 x Schistosoma bovis Sonsino, 1876 interactions, with new cercarial emergence and genetic patterns. Parasitol Res. 2020; 2189–2205. doi:10.1007/s00436-020-06709-0

38. Oey H, Zakrzewski M, Gravermann K, Young ND, Korhonen PK, Gobert GN, et al. Whole-genome sequence of the bovine blood fluke *Schistosoma bovis* supports interspecific hybridization with *S. haematobium*. Platt RN, editor. PLOS Pathog. 2019;15: e1007513. doi:10.1371/journal.ppat.1007513

39. Pitchford RJ. A check list of definitive hosts exhibiting evidence of the genus *schistosoma* weinland, 1858 acquired naturally in africa and the middle east. J Helminthol. 1977;51: 229–251. doi:10.1017/S0022149X00007574

40. Rollinson D. The genus *Schistosoma*: a taxonomic appraisal. Biol Schistosomes Genes Latrines. 1987.

41. Pitchford RJ, Visser PS. The role of naturally infected wild rodents in the epidemiology of schistosomiasis in the Eastern Transvaal. Trans R Soc Trop Med Hyg. 1962;56: 126–135. doi:10.1016/0035-9203(62)90139-6

42. Catalano S, Sène M, Diouf ND, Fall CB, Borlase A, Léger E, et al. Rodents as Natural Hosts of Zoonotic *Schistosoma* Species and Hybrids: An Epidemiological and Evolutionary Perspective From West Africa. J Infect Dis. 2018; jiy029. doi:10.1093/infdis/jiy029

43. Léger E, Borlase A, Fall CB, Diouf ND, Diop SD, Yasenev L, et al. Prevalence and distribution of schistosomiasis in human, livestock, and snail populations in northern Senegal: a One Health epidemiological study of a multi-host system. Lancet Planet Health. 2020;4: e330–e342. doi:10.1016/S2542-5196(20)30129-7

44. Webster JP, Gower CM, Knowles SCL, Molyneux DH, Fenton A. One health - an ecological and evolutionary framework for tackling Neglected Zoonotic Diseases. Evol Appl. 2016;9: 313–333. doi:10.1111/eva.12341

45. Grevelding CG, Sommer G, Kunz W. Female-specific gene expression in *Schistosoma mansoni* is regulated by pairing. Parasitology. 1997;115: 635–640. doi:10.1017/S0031182097001728

46. Leutner S, Oliveira KC, Rotter B, Beckmann S, Buro C, Hahnel S, et al. Combinatory Microarray and SuperSAGE Analyses Identify Pairing-Dependently Transcribed Genes in Schistosoma mansoni Males, Including Follistatin. Jones MK, editor. PLoS Negl Trop Dis. 2013;7: e2532. doi:10.1371/journal.pntd.0002532

47. Kincaid-Smith J, Boissier J, Allienne JF, Oleaga A, Djuikwo-Teukeng F, Toulza E. A Genome Wide Comparison to Identify Markers to Differentiate the Sex of Larval Stages of *Schistosoma haematobium*, *Schistosoma bovis* and their Respective Hybrids. PLoS Negl Trop Dis. 2016;10: e0005138. doi:10.1371/journal.pntd.0005138

48. Silva ML, Vicente FS, Avelino IC, Martin VR. Susceptibility of *Planorbarius metidjensis* from Portugal and Spain to *Schistosoma bovis* from Salamanca, Spain. Malacologia. 1977;16: 251–254.

49. Boissier J, Rivera ER, Moné H. Altered behavior of the snail Biomphalaria glabrata as a result of infection with *Schistosoma mansoni*. J Parasitol. 2003;89: 429–433. doi:10.1645/0022-3395(2003)089[0429:ABOTSB]2.0.CO;2

50. Boissier J, Chlichlia K, Digon Y, Ruppel A, Moné H. Preliminary study on sex-related inflammatory reactions in mice infected with *Schistosoma mansoni*. Parasitol Res. 2003;91: 144–150. doi:10.1007/s00436-003-0943-1

51. Théron a, Pages JR, Rognon a. *Schistosoma mansoni*: distribution patterns of miracidia among *Biomphalaria glabrata* snail as related to host susceptibility and sporocyst regulatory processes. Exp Parasitol. 1997;85: 1–9. doi:10.1006/expr.1996.4106

52. Beltran S, Galinier R, Allienne JF, Boissier J. Cheap, rapid and efficient DNA extraction method to perform multilocus microsatellite genotyping on all *Schistosoma mansoni* stages. Mem Inst Oswaldo Cruz. 2008;103: 501–503. doi:S0074-02762008000500017 [pii]

53. Van den Broeck F, Geldof S, Polman K, Volckaert FAM, Huyse T. Optimal sample storage and extraction procotols for reliable multilocus genotyping of the human parasite *Schistosoma mansoni*. Infect Genet Evol. 2011;11: 1413–1418. doi:10.1016/j.meegid.2011.05.006

54. Webster BL, Rollinson D, Stothard JR, Huyse T. Rapid diagnostic multiplex PCR (RD-PCR) to discriminate *Schistosoma haematobium* and *S. bovis*. J Helminthol. 2010;84: 107–114. doi:10.1017/S0022149X09990447

55. Giardine B, Riemer C, Hardison RC, Burhans R, Elnitski L, Shah P, et al. Galaxy: A platform for interactive large-scale genome analysis. Genome Res. 2005;15: 1451–1455. doi:10.1101/gr.4086505

56. Goecks J, Nekrutenko A, Taylor J, Afgan E, Ananda G, Baker D, et al. Galaxy: a comprehensive approach for supporting accessible, reproducible, and transparent computational research in the life sciences. Genome Biol. 2010;11. doi:10.1186/gb-2010-11-8-r86

57. Blankenberg D, Gordon A, Von Kuster G, Coraor N, Taylor J, Nekrutenko A, et al. Manipulation of FASTQ data with galaxy. Bioinformatics. 2010;26: 1783–1785. doi:10.1093/bioinformatics/btq281

58. Martin M. Cutadapt removes adapter sequences from high-throughput sequencing reads. EMBnet.journal. 2011;17: 10. doi:10.14806/ej.17.1.200

59. Dobin A, Davis CA, Schlesinger F, Drenkow J, Zaleski C, Jha S, et al. STAR: Ultrafast universal RNA-seq aligner. Bioinformatics. 2013;29: 15–21. doi:10.1093/bioinformatics/bts635

60. Young ND, Jex AR, Li B, Liu S, Yang L, Xiong Z, et al. Whole-genome sequence of *Schistosoma haematobium*. Nat Genet. 2012;44: 221–225. doi:10.1038/ng.1065

61. Trapnell C, Williams BA, Pertea G, Mortazavi A, Kwan G, van Baren MJ, et al. Transcript assembly and quantification by RNA-Seq reveals unannotated transcripts and isoform switching during cell differentiation. Nat Biotechnol. 2010;28: 511.

62. Anders S, Pyl PT, Huber W. HTSeq-A Python framework to work with high-throughput sequencing data. Bioinformatics. 2015;31: 166–169. doi:10.1093/bioinformatics/btu638

63. Love MI, Anders S, Huber W. Differential analysis of count data - the DESeq2 package. Genome Biology. 2014. doi:110.1186/s13059-014-0550-8

64. R Core Team. R: A language and environment for statistical computing. R Foundation for Statistical Computing, Vienna, Austria; 2020. Available: URL https://www.R-project.org/

65. Conesa A, Götz S, García-Gómez JM, Terol J, Talón M, Robles M. Blast2GO: a universal tool for annotation, visualization and analysis in functional genomics research. Bioinformatics. 2005;21: 3674–3676.

66. Leger E, Webster JP. Hybridizations within the Genus *Schistosoma*: implications for evolution, epidemiology and control. Parasitology. 2017;144: 65–80. doi:10.1017/S0031182016001190

67. Huyse T, Van den Broeck F, Hellemans B, Volckaert FAM, Polman K. Hybridisation between the two major African schistosome species of humans. Int J Parasitol. 2013;43: 687–689. doi:10.1016/j.ijpara.2013.04.001

68. Hoffmann KF. An historical and genomic view of schistosome conjugal biology with emphasis on sex-specific gene expression. Parasitology. 2004;128: S11–S22. doi:10.1017/S0031182004006213

69. LoVerde PT, Andrade LF, Oliveira G. Signal transduction regulates schistosome reproductive biology. Curr Opin Microbiol. 2009;12: 422–428. doi:10.1016/j.mib.2009.06.005

70. Osman A, Niles EG, Verjovski-Almeida S, LoVerde PT. *Schistosoma mansoni* TGF-β receptor II: Role in host ligand-induced regulation of a schistosome target gene. PLoS Pathog. 2006;2: 0536–0550. doi:10.1371/journal.ppat.0020054

71. LoVerde PT, Osman A, Hinck A. Schistosoma mansoni: TGF-β signaling pathways. Exp Parasitol. 2007;117: 304–317. doi:10.1016/j.exppara.2007.06.002

72. Knobloch J, Beckmann S, Burmeister C, Quack T, Grevelding CG. Tyrosine kinase and cooperative TGFβ signaling in the reproductive organs of *Schistosoma mansoni*. Exp Parasitol. 2007;117: 318–336. doi:10.1016/j.exppara.2007.04.006

73. Beckmann S, Quack T, Burmeister C, Buro C, Long T, Dissous C, et al. *Schistosoma mansoni*: Signal transduction processes during the development of the reproductive organs. Parasitology. 2010;137: 497–520. doi:10.1017/S0031182010000053

74. Freitas TC, Jung E, Pearce EJ. TGF-β signaling controls embryo development in the parasitic flatworm Schistosoma mansoni. PLoS Pathog. 2007;3: 489–497. doi:10.1371/journal.ppat.0030052

75. Andrade LF de, Mourão M de M, Geraldo JA, Coelho FS, Silva LL, Neves RH, et al. Regulation of *Schistosoma mansoni* Development and Reproduction by the Mitogen-Activated Protein Kinase Signaling Pathway. PLoS Negl Trop Dis. 2014;8: e2949. doi:10.1371/journal.pntd.0002949

76. Storz G, Imlayt JA. Oxidative stress. Curr Opin Microbiol. 1999;2: 188–194.

77. Capy P, Gasperi G, Biémont C, Bazin C. Stress and transposable elements: co-evolution or useful parasites? Heredity. 2000;85: 101–106. doi:10.1046/j.1365-2540.2000.00751.x

78. Platt RN, McDew-White M, Le Clec'h W, Chevalier FD, Allan F, Emery AM, et al. Ancient Hybridization and Adaptive Introgression of an Invadolysin Gene in Schistosome Parasites. Shapiro B, editor. Mol Biol Evol. 2019;36: 2127–2142. doi:10.1093/molbev/msz154

79. Grossman a I, Short RB, Cain GD. Karyotype evolution and sex chromosome differentiation in Schistosomes (Trematoda, Schistosomatidae). Chromosoma. 1981;84: 413–30. doi:10.1007/BF00286030

80. Kincaid-Smith J, Tracey A, de Carvalo Augusto R, Bulla I, Holroyd N, Rognon A, et al. Whole genome sequencing and morphological analysis of the human-infecting schistosome emerging in Europe reveals a complex admixture between *Schistosoma haematobium* and *Schistosoma bovis* parasites. bioRxiv. 2018. doi:10.1101/387969

81. Kincaid-Smith J, Tracey A, Augusto R, Bulla I, Holroyd N, Rognon A, et al. Morphological and genomic characterisation of the hybrid schistosome infecting humans in Europe reveals a complex admixture between *Schistosoma haematobium and Schistosoma bovis parasites*. Genomics; 2018 Aug. doi:10.1101/387969

82. Boon Nam, Mbow M, Paredis L, Moris P, Sy I, Maes T, et al. No barrier breakdown between human and cattle schistosome species in the Senegal River Basin in the face of hybridisation. Int J Parasitol. 2019;49: 1039–1048. doi:10.1016/j.ijpara.2019.08.004

83. Pennance T, Allan F, Emery A, Rabone M, Cable J, Garba AD, et al. Interactions between *Schistosoma haematobium* group species and their *Bulinus* spp. intermediate hosts along the Niger River Valley. Parasit Vectors. 2020;13: 268. doi:10.1186/s13071-020-04136-9

84. Tchuem Tchuenté LA, Southgate VR, Jourdane J, Kaukas A, Vercruysse J. Hybridisation between the digeneans *Schistosoma haematobium* and *S. mattheei* : viability of hybrids and their development in sheep. Syst Parasitol. 1997;36: 123–131. doi:10.1023/A:1005705030619

85. Detwiler JT, Criscione CD. An Infectious Topic in Reticulate Evolution: Introgression and Hybridization in Animal Parasites. Genes. 2010;1: 102–123. doi:10.3390/genes1010102

86. King KC, Stelkens RB, Webster JP, Smith DF, Brockhurst MA. Hybridization in Parasites: Consequences for Adaptive Evolution, Pathogenesis, and Public Health in a Changing World. Rall GF, editor. PLOS Pathog. 2015;11: e1005098. doi:10.1371/journal.ppat.1005098

87. Tchuem Tchuenté L-A, Southgate VR, Jourdane J, Webster BL, Vercruysse J. *Schistosoma intercalatum*: an endangered species in Cameroon? Trends Parasitol. 2003;19: 389–393. doi:10.1016/S1471-4922(03)00193-4

Supplementary file captions:

Supplementary Table S1: Metrics of the RNA-sequencing reads processing

Supplementary File S1: Transcriptome analysis related information, including, the full annotation of the reference transcriptome representative of *S. haematobium* and *S. bovis* assembled for this study (sheet1); transcript (*i.e.*, HTseq) counts (sheet2); DESeq2 results for male *S. haematobium* (sheet3), female *S. haematobium* (sheet4), male *S. bovis* (sheet5) and female *S. bovis* (sheet6); annotations associated to each differentially expressed genes identified (sheet7) and Gene Ontologies enriched in differentially expressed genes in male *S. haematobium* (sheet8).

Figure1

Click here to access/download;Figure;Fig1.tif

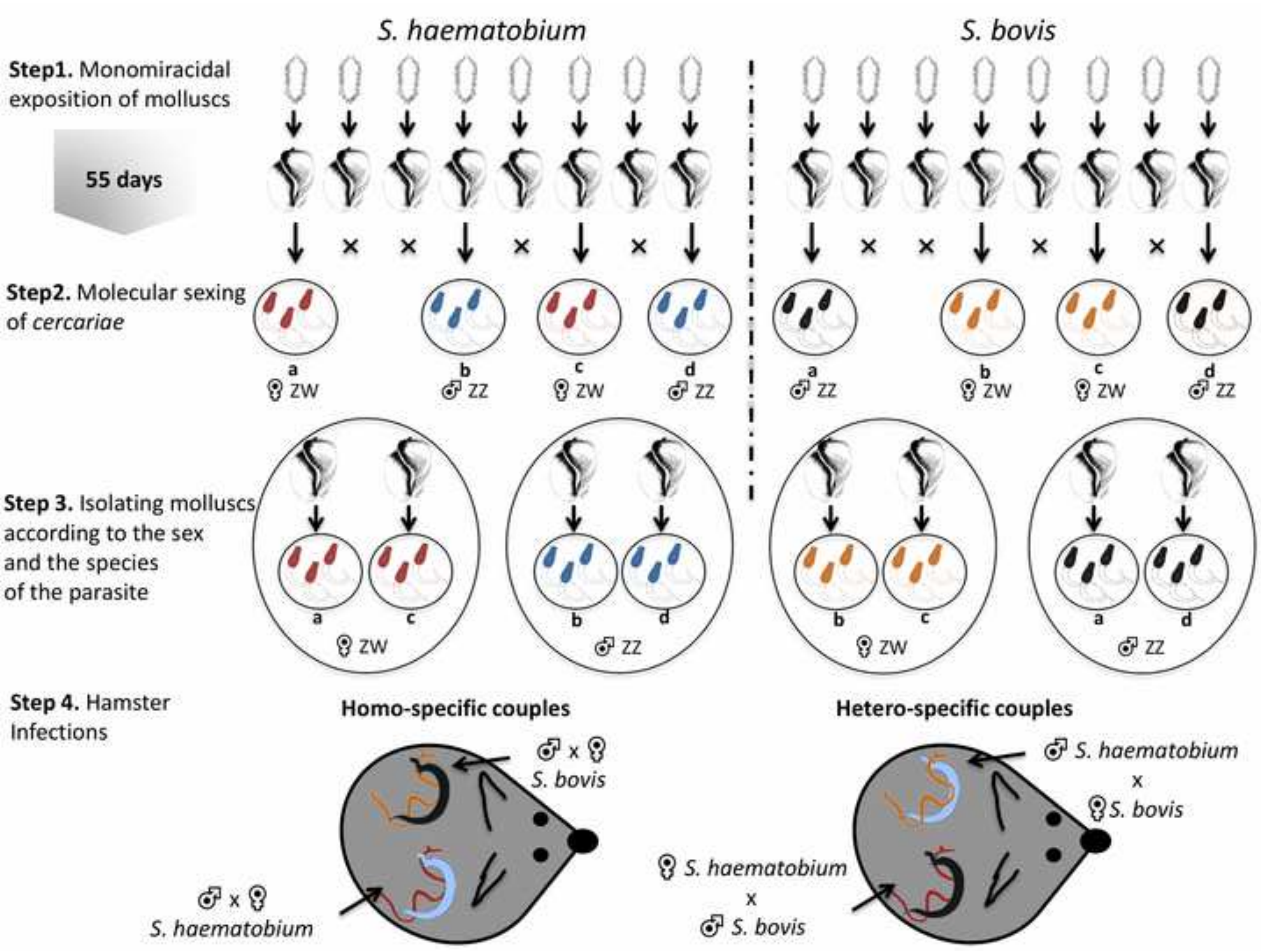

Figure2

Click here to access/download;Figure;Fig2.tif

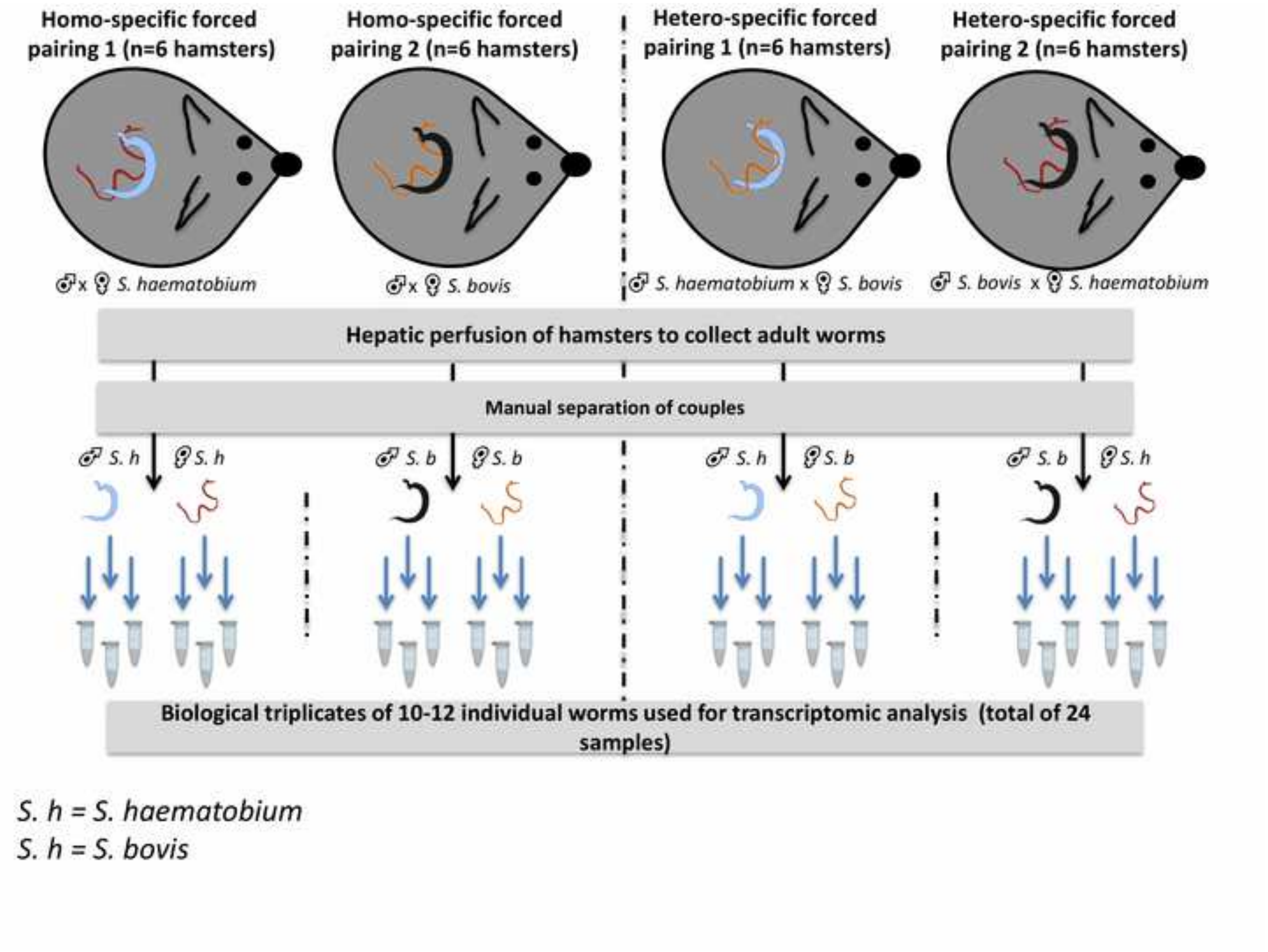

Page 0

Figure3

Click here to access/download;Figure;Fig3.tif

Page 0

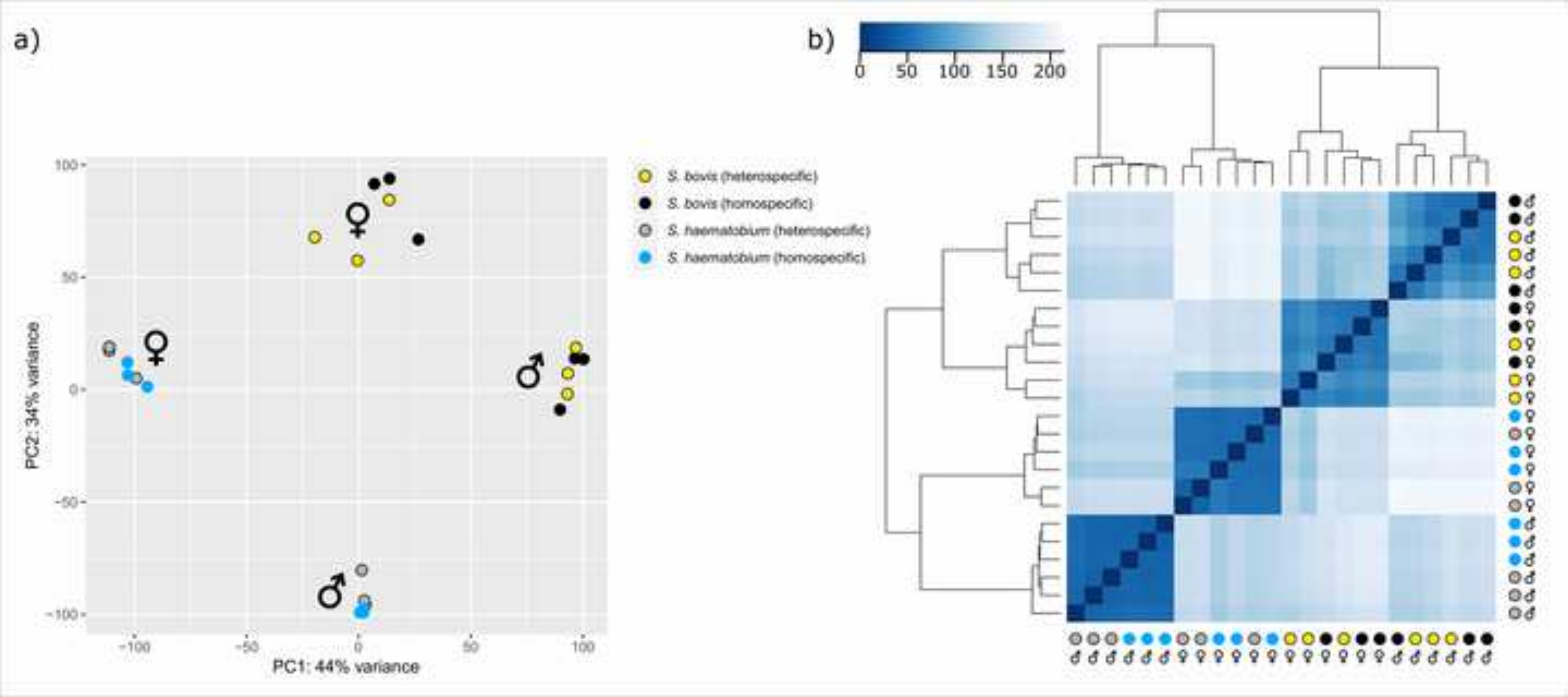

Click here to access/download;Figure;Fig4.tif

Page 0

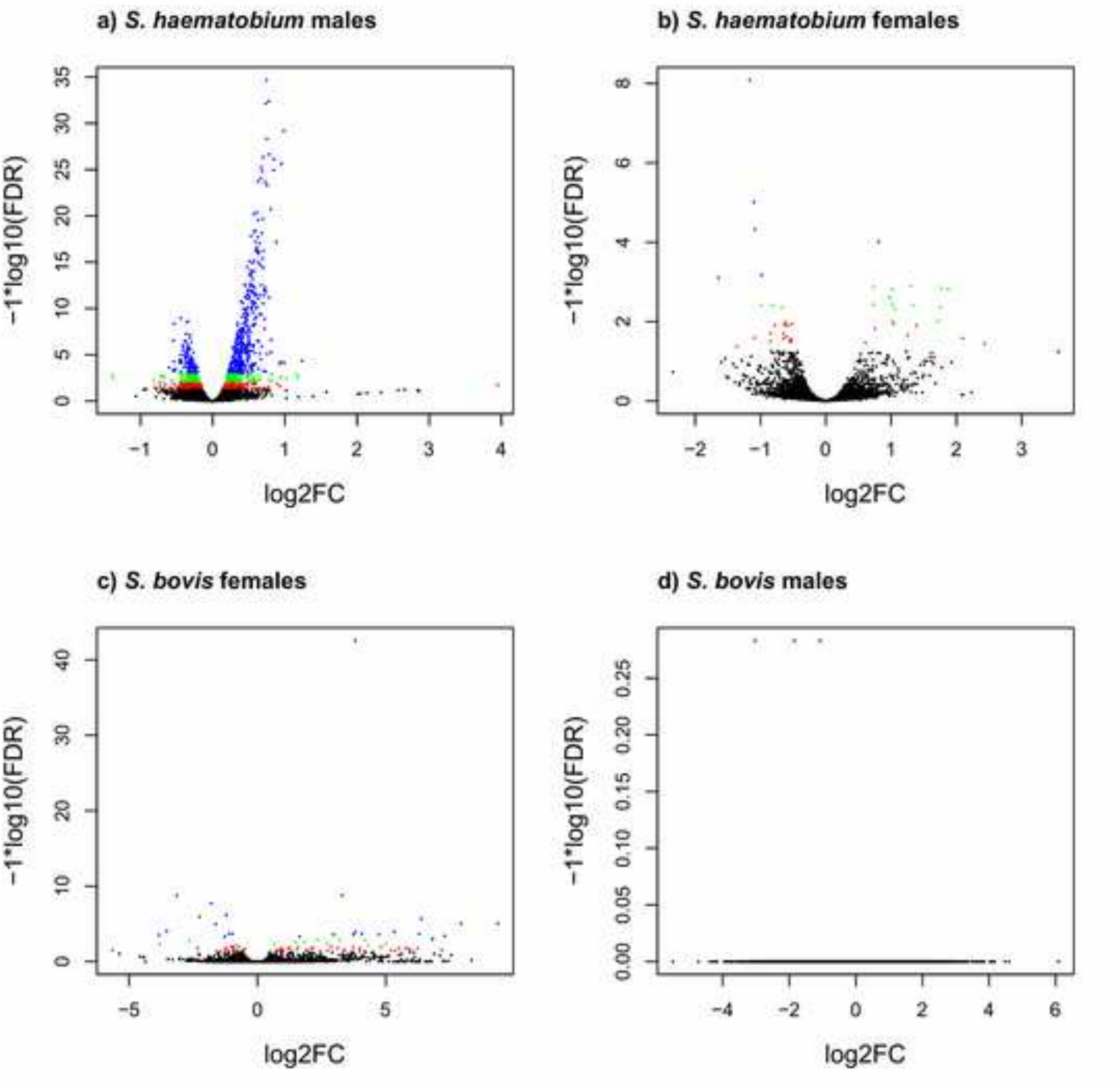

Click here to access/download;Figure;Fig5.tif

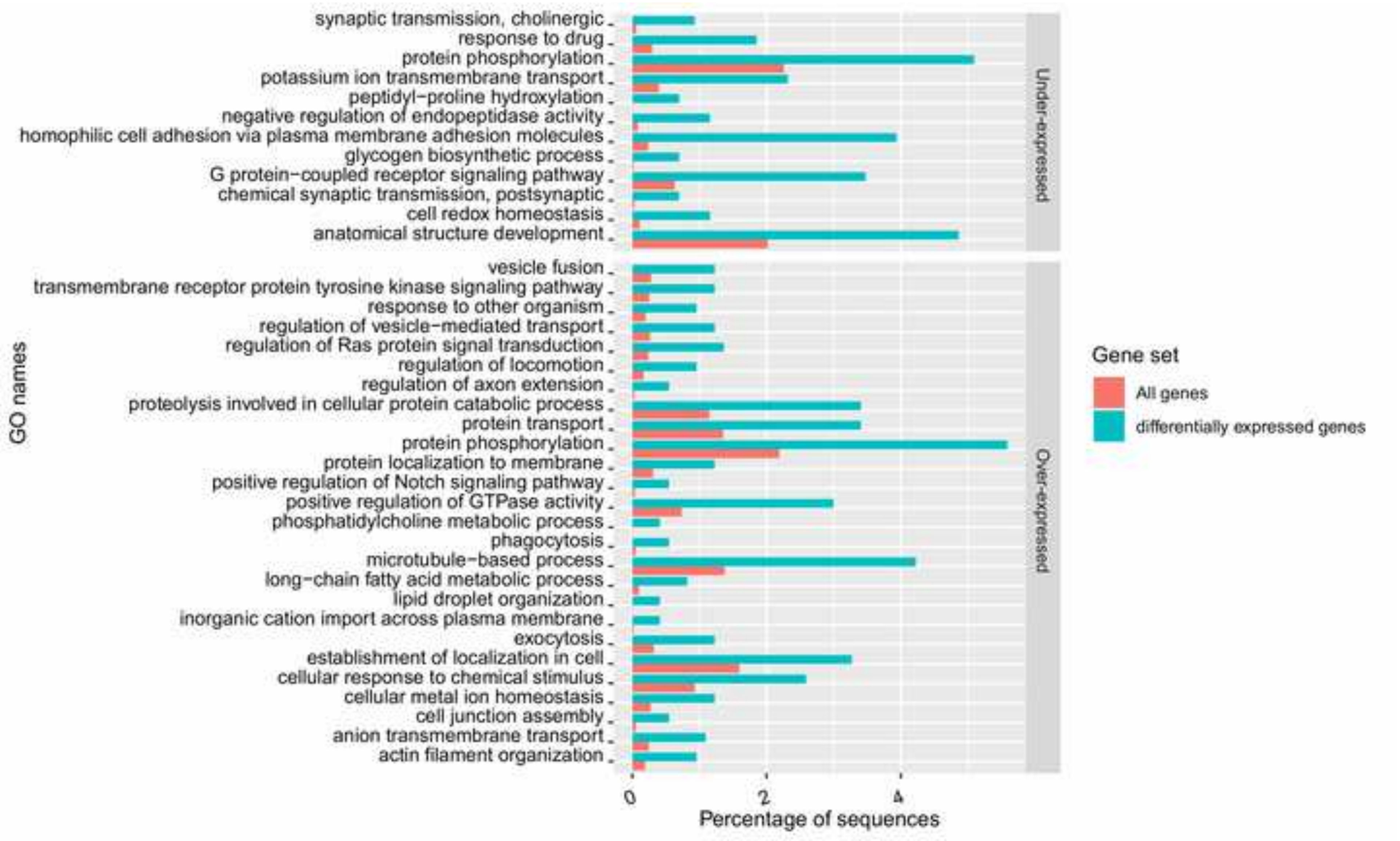

Supporting Information Table S1

Click here to access/download
Supporting Information
Supplementary Table S1.docx

Supporting Information File S1

Click here to access/download
Supporting Information
Supplementary File S1.xlsx

Dear Dr Lamberton,

Please find attached a significantly revised version of our manuscript PNTD-D-20-01594 taking into account the reviewers concerns.

We would like to thank you and anonymous reviewers for the detailed and thoughtful criticism on the manuscript. We have modified the manuscript according to reviewers' suggestions. All changes are indicated in the manuscript and you will find below our responses after each point that were raised by the two reviewers. To ease the reading, the major reviewer's comments have been numbered and reported as comments in the revised manuscript to locate the corrections using the "search" command.

We have substantially revised the title, authors summary, materiel and methods section as well as some result sections, discussion and conclusion to clarify and focus shortcomings. We believe this new version of the manuscript addresses the concerns of the reviewers and represents a substantially improved article.

The level of English and grammar within this paper has also been revised and we hope that you will find it suitable for publication in *Plos Neglected Tropical Disease.*

We appreciate the time that you and anonymous reviewers have taken and would like to thank you for the constructive editorial process.

Sincerely,

Dr Mathieu-Bégné on behalf of all co-authors.

Dear Mrs Mathieu-Bégné,

Thank you very much for submitting your manuscript "Pre-zygotic isolation mechanisms between *Schistosoma haematobium* and *Schistosoma bovis* parasites: from mating interactions to differential gene expression" for consideration at PLOS Neglected Tropical Diseases. As with all papers reviewed by the journal, your manuscript was reviewed by members of the editorial board and by several independent reviewers. In light of the reviews (below this email), we would like to invite the resubmission of a significantly-revised version that takes into account the reviewers' comments.

All three reviewers have commented on the level of English and grammar within this paper, that makes it hard to properly review this paper. I would recommend that in addition to all that scientific comments and specific language comments included that all authors really help rewrite this manuscript and if needed that you ask a native English speaker to help with the next version. I will send this out to reviewers again upon resubmission if the level of English has improved, but that really does need to be addressed.

We cannot make any decision about publication until we have seen the revised manuscript and your response to the reviewers' comments. Your revised manuscript is also likely to be sent to reviewers for further evaluation.

When you are ready to resubmit, please upload the following:

[1] A letter containing a detailed list of your responses to the review comments and a description of the changes you have made in the manuscript. Please note while forming your response, if your article is accepted, you may have the opportunity to make the peer review history publicly available. The record will include editor decision letters (with reviews) and your responses to reviewer comments. If eligible, we will contact you to opt in or out.

[2] Two versions of the revised manuscript: one with either highlights or tracked changes denoting where the text has been changed; the other a clean version (uploaded as the manuscript file).

Important additional instructions are given below your reviewer comments.

Please prepare and submit your revised manuscript within 60 days. If you anticipate any delay, please let us know the expected resubmission date by replying to this email. Please note that revised manuscripts received after the 60-day due date may require evaluation and peer review similar to newly submitted manuscripts.

Thank you again for your submission. We hope that our editorial process has been constructive so far, and we welcome your feedback at any time. Please don't hesitate to contact us if you have any questions or comments.

Sincerely,

Poppy Lamberton

Deputy Editor

PLOS Neglected Tropical Diseases

****

Key Review Criteria Required for Acceptance?

As you describe the new analyses required for acceptance, please consider the following:

Methods

-Are the objectives of the study clearly articulated with a clear testable hypothesis stated?

-Is the study design appropriate to address the stated objectives?

-Is the population clearly described and appropriate for the hypothesis being tested?

-Is the sample size sufficient to ensure adequate power to address the hypothesis being tested?

-Were correct statistical analysis used to support conclusions?

-Are there concerns about ethical or regulatory requirements being met?

Reviewer #1:

-Are the objectives of the study clearly articulated with a clear testable hypothesis stated?

Yes

<u>Comment 1</u>

-Is the study design appropriate to address the stated objectives?

Yes but more discussion is needed on the limitations of the study design

<u>Author response:</u> We agree, and the discussion has been amended to include limitations of the study design. In particular we now discuss the impact of the low number of replicates (due to premature death of hamsters) on the conclusions drawn in the experiment 1 (Comment 1). Although we cannot conclude with certitude for this particular mate choice, this does not affect our general observation of random mate choice.

<u>Comment 2</u>

-Is the population clearly described and appropriate for the hypothesis being tested?

More information is needed on the strains used and the details of the experiments

<u>Author response:</u> Thank you for highlighting this unclear section; we have amended the text to help readers in understanding the experimental procedure and strains used. We added more information regarding the strains in the material and method section of our revised version of the manuscript (Comment 2.1). Concerning details of the experiments, we have now divided the protocol in three sections ("Experimental infections", "Mate choice analysis" and "Forced reciprocal pairings") that bring in more details and clarify (Comment 2.2, Comment 2.3 and Comment 2.4 respectively) common experimental procedure and how each objective was addressed (Comment 2.4).

We have amended Figure 1 to include an example of both homo and hetero-specific pairs where each species and sexes are represented with a different colour. In addition, we propose a new figure (Fig 2) to allow a better understanding of the procedure for homo and hetero- forced pairings.

-Is the sample size sufficient to ensure adequate power to address the hypothesis being tested?

Yes

-Were correct statistical analysis used to support conclusions?

I would like this checked by a statistician

-Are there concerns about ethical or regulatory requirements being met?

No

Reviewer #2:

<u>Comment 3:</u>

- The overall objective is clear but the hypotheses could be more clearly formulated in the introduction. For example, somewhere in the paper it was stated that the authors expected the opposite outcome (more transcriptomic changes in females than in males), so this hypothesis could have been included at the end of the introduction, instead of the vague kind of conclusion in line 194-197. Additional testable hypotheses can be formulated.

<u>Author response:</u> Thank you very much for taking time to review our paper and for your thorough comments and feedback; they were very helpful. We now have more clearly formalized hypothesis and predictions at the end of the introduction (Comment 3).

We suggest that in line to the current knowledge in schistosomes – mating does not seem to be a major reproductive isolation mechanism since hybrids are frequent in the field and parental species readily pair in the lab. However mating choice are necessary not only to assess the frequency of the phenomenon but also if any dominance could explain biased patters observed in the field. Moreover, as females undergo strong developmental changes upon pairing, we mentioned that we first expected finding a stronger molecular response in comparison to males.

<u>Comment 4</u>:

- The study design is appropriate although some issues arise regarding statistical power. The results discussed in line 362 (premature death of two hamsters) does raise the question whether 5 replica's (5 hamsters) is enough to make robust conclusions as in this particular case no reliable statistics can be done for S. *bovis* males' choice.

__**Author response:**__ **We do agree that we cannot make any robust conclusions for the result of the Experiment 3 due to the premature death of two hamsters and thus the poor statistical power (see also response to Comment 1).**

This was explicitly stated in the results section (and now including in the Table 3, Comment 4.1) but also now stated in discussion section. We therefore suggest that this combination has to be tested in the future, but, although this hampers our ability to infer *S. bovis* males' choice, our overall results strongly indicate that mating occur in a random fashion between the two species whatever the direction of the cross (Comment 4.2).

- No concerns about ethical issues.

For more comments on the Methods section see General Comments.

__**Author response:**__ **We have taken particular attention in the comments made in the General Comments and have amended the manuscript accordingly. Thank you for the helpful thoughts and the time spent at editing our manuscript.**

Reviewer #3:
Methods seem appropriate

Results

-Does the analysis presented match the analysis plan?

-Are the results clearly and completely presented?

-Are the figures (Tables, Images) of sufficient quality for clarity?

Reviewer #1:

-Does the analysis presented match the analysis plan?

Yes

Comment 5:

-Are the results clearly and completely presented?

Improvement is needed for clarity of the results to allow interpretation

Author response: **We added some substantial changes to the manuscript to address this issue, notably by amending the Table 2 and the main text of the result section (e.g., Comment 5.1 for clarification of Table 2 regarding crosses made).**

-Are the figures (Tables, Images) of sufficient quality for clarity? More information and clarity is needed as detailed in the attached document

Author response: **This has been addressed as proposed in the attached document, which we think, helped us a lot to improve the manuscript. To improve the clarity of the tables, we added information on each paired and single worm sex, hence allowing the readers to better identify tested combinations (Comment 5.1). Information was also included in the captions. For instance regarding the expected number of worms under random mating hypothesis, an example was given in material and method section and is now referred to**

in Table 2 caption (Comment 5.2). A new figure 2 has also been added to clarify the experimental mating procedure (see also response to Comment 2).

Reviewer #2:

The analysis is sound and the results are well presented.

<u>Comment 6:</u>

Table 2 is redundant I would say.

<u>Author response:</u> Thank you for your feedback. We agree with your comment. Former Table 2 has been removed (Comment 6.1) and all relevant information for readers are now presented in the main text (Comment 6.2).

For more comments on the Results section see General Comments.

<u>Author response:</u> Thank you again. We have taken note of the General Comments section and have amended the manuscript accordingly.

Reviewer #3: Analysis matches described goals of the work.

REsults are clearly presented.

Figures are sufficient quality

Conclusions

-Are the conclusions supported by the data presented?

-Are the limitations of analysis clearly described?

-Do the authors discuss how these data can be helpful to advance our understanding of the topic under study?

-Is public health relevance addressed?

Reviewer #1:

-Are the conclusions supported by the data presented?

Yes

Comment 7

-Are the limitations of analysis clearly described?

No

Author response: We agree. We have paid particular attention to bring in the limitations of our work. We did so in the discussion section, for mate choice experiments (Comment 7.1), for the interpretation of biological processes revealed by the transcriptomic analysis (Comment 7.2) and regarding the need to investigate the post-zygotic isolation mechanisms to better understand the hetero-specific pairing patterns in the field (Comment 7.3) (see also response to Comment 1).

-Do the authors discuss how these data can be helpful to advance our understanding of the topic under study?

Ye

-Is public health relevance addressed?

Yes

Further comments on the discussion are available in the attached

Author response: **We have taken in consideration all comments and have amended the manuscript accordingly.**

Reviewer #2:

Comment 8

As put in my General comments, the Discussion needs reworking and should be more substantiated. The conclusions are not always clear or strong, I miss more references to similar studies, but also a proper discussion on the limitations and recommendations for future research are missing. The public health relevance is not really thoroughly discussed.

Author response: **Thank you for these constructive comments.**

The discussion as been substantially modified and we have brought in information on similar studies and limitations (Comment 8.1, Comment 8.2, and Comment 8.3).

We have recommended several times throughout the manuscript that further studies would be needed when our experiments did not allow a strong conclusion (Comment 7.1 and 7.2). Concerning the public health relevance, we now explain better the concerns that our findings bring in terms of potential hybridization frequency (Comment 8.4) and the consequences on the parasites virulence and hybrids vigour (Comment 8.5)

Reviewer #3:

Conclusions are supported by the data. They speculate quite a bit in the discussion about the potential importance of various DE genes. But this type of speculation is rampant in gene expression papers and they don't make any hard conclusions.

<u>Author response:</u> Thank you very much for taking time to review our paper and for thoughtful criticism. We agree with the speculations inherent to gene expression analysis and have nuanced our conclusions and discussed our hypothesis in respect to the literature (cf Comment 7.2).

Editorial and Data Presentation Modifications?

Use this section for editorial suggestions as well as relatively minor modifications of existing data that would enhance clarity. If the only modifications needed are minor and/or editorial, you may wish to recommend “Minor Revision” or “Accept”.

Reviewer #1: The main modifications are needed in the clarity of the data and how it is presented. Comments are in the attached document.

<u>Author response:</u> We are very grateful for your feedback and have taken particular attention to modify the manuscript accordingly. Thank you.

In particular, regarding some major comments we found in the attached document you will find our detailed answer below in order to explain some changes we made:

1) **Overall, we re-structured the Material and Method section following your recommendation to clarify following the structure used in Result section**

2) **More detail has been given on the experimental procedure.**

3) **In order to gain clarity, we amended table content and table caption. Please note that it is normal that there is no total sum in the Table 2 (former Table 3) since Chi-square test involved comparison of expected number of pairs under random mating hypothesis to observed one at the level of the host (row) but the statistic of the test and its significance is computed considering all the hosts of a particular experiment. Similarly even if expected number of hetero-specific pairs could seem high some time compared to observed one, the aim of**

the test is to compare homo- and hetero-specific pairs number as expected if worms were mating randomly and considering the initial number of worms provided.

4) **We considered your comment regarding the fact that we could not distinguished between mate choice and competition all along the manuscript.**

5) **A more extensive discussion about the asymmetry between some *Schistosoma* crosses as observed in the field is now provided in a dedicated paragraph of the discussion.**

Reviewer #2: (No Response)

Reviewer #3: (No Response)

Summary and General Comments

Use this section to provide overall comments, discuss strengths/weaknesses of the study, novelty, significance, general execution and scholarship. You may also include additional comments for the author, including concerns about dual publication, research ethics, or publication ethics. If requesting major revision, please articulate the new experiments that are needed.

Reviewer #1:

<u>**Comment 9**</u>

This is an important and interesting study that shows the lack of pre zygotic isolation between S. *haematobium* and S. *bovis* supporting inter-species hyrbidisation an important scenario for human and animal health in Africa. Although, I consider the study of value and there is a substantial body of high quality of work, which is not easy to do (particularly the generation of the isolates and the mating experiments) the paper needed considerable improvements before it can be reviewed further. The authors should consider that the readers will not be familiar with these types of experiments and there is a need to add the detail that allows the readers to understand the experimental procedures and the data produced. There are also english and gramma errors many of which can be improved with careful reading. In the attached these are highlighted in the text showing the errors and where changes are needed. I have tried to cover the whole document but due to time some places may have been missed and careful reading and revision is needed. There are also comments on the attached to highlight where more clarity is needed and also where further information or discuss is warranted. The discussion is also very long and could be condensed by not repeating what is in the results.

<u>**Author response:**</u>

Thank you for all the very useful comments and the precious time you have spent on this. We think we have considerably improved the paper, by amending the parts of the manuscript that needed clarification and by providing further information so that readers can better understand the experimental procedures and the data produced. For that we have added details on the experiments (e.g., Comment 9.1, but most of the Material and Method section has been re-written and re-organized, cf Comment 2).

We also amended Figure 1 and added a new figure presenting the experiments for the transcriptomic analysis (Figure 2, Comment 9.2).

The English and grammar errors have been carefully revised.

Finally, we have reduced some parts of the discussion that were redundant with the results (Comment 9.3-9.6) or condensed some very long parts to be more straightforward (Comment 9.7-9.9). However, regarding the interest and thorough comments of the second reviewer we have also added supplementary discussion on the asymmetry in the direction of hybridization and introgression between schistosomes.

Reviewer #2:

<u>Comment 10:</u>

This is a very interesting study, with interesting results. Experimental infections in hamsters show that there are no pre-zygotic barriers to mating between the human Schistosoma *haematobium* and the animal S. *bovis* parasite, neither are there any major transcriptomic responses following hetero-specific pairings. Even though a previous study by Webster and colleagues already showed that S. *bovis* and S. *haematobium* readily paired in laboratory hamsters (to which the present authors not refer, which I think is an omission), this is the first time that any transcriptomic study is done on hybrid crosses.

I was frustrated by the sloppy grammar and spelling throughout the entire manuscript, which gave the impression that the authors were in a hurry to submit this manuscript or they didn't really care about this.

I also lack a discussion on the asymmetry in the direction of hybridisation and introgression between schistosomes, which is sometimes even unidirectional, but nothing is mentioned on this.

Also, it is repeatedly said that isolation 'by the host' is apparently the only barrier to hybridisation between these two schistosome species, but it is never specified which host they mean with that, the final or the intermediate one. Since mate choice and reproduction takes place in the final host, this one is of particular interest of course, but if the different intermediate hosts are not sympatric, then hybridisation will also be less frequent (if they final host does not move around too much). For S. *bovis* and S. *haematobium* this is more complicated, as intermediate host specificity of the latter varies with geography, but still this should be properly discussed as it could also explain why we see such regional differences in the distribution of hybrids.

Then finally, I also have some problems with parts of the discussion: some questions remain unanswered (e.g. why would only S. *haematobium* males in heterospecific pairing have this transcriptomic response?), the statistical power, and with the fact that I miss the broader picture, the reference to all the previous work on schistosome mating experiments. So therefore I think that these concerns should be addressed first before it can be accepted for publication.

<u>Author response:</u> Thank you for all these very useful comments. We have included the reference to Webster and colleagues' paper that showed that *S. bovis* and *S. haematobium* readily pair in laboratory hamster, which support our predictions (Comment 10.1). More generally, we amended the manuscript with more extensive references to previous works

on schistosome mating experiments, which is most noticeable in the discussion section (e.g. Comment 10.2). Following your advice, we have added some discussion about the potential asymmetry in the direction of hybridization and introgression between schistosomes. We now discuss that although our data suggest that hybridization may be frequent in the field, recent genomic evidence suggest that introgressed hybrids may be ancient and that there is a strong bias in the hybrids profile (that also depend on the foci and the dynamics that are in place) (Comment 10.3). We then propose that post-zygotic mechanisms are the most likely to be shaping the outcome of hybridization (Comment 10.4).

We agree with you and acknowledge that there is indeed a lot to discuss about. However, in response to other comments about the length of the discussion we have tried to reach a middle ground by keeping the focus here on the pre-zygotic isolation mechanisms. We have specified wherever required that the final host is the one responsible for species reproductive isolation as it is only in that host that sexual reproduction occurs (Comment 10.5.0-10.5.2). Even though the idea of intermediate host specificity is highly relevant and important, we have hence preferred to keep on pre-zygotic barrier in the "site" in which species can interact. Moreover, as you underlie the situation with Sh and Sb is a bit more complex, both species can use the same mollusc (*Bulinus*) and it is difficult to predict the host-parasite compatibility outcome. This could be the entire focus of a dedicated study.

Regarding the biological explanation on why only male S. *haematobium* displayed more DEGs, we acknowledge that this is not straightforward and that we can only speculate. We thus re-wrote the paragraph dedicated to this topic in discussion section (Comment 10.6) in order to provide potential explanations: This particular response of male *S. haematobium* is most probably due to technical bias (replicate variability); the amount

of DEGs seems high but does not imply a strong transcriptomic response compared to females worms since the log2Fold Change are low and/or this molecular plasticity in expression of genes is a mechanism by which *S. haematobium* males manage to be more competitive (compared to females from both species and *S. bovis* males) in hetero-specific pairing.

Regarding the mate choice experiment and in response to other comments we also now discuss more directly the issue raised by the statistical power in our study (Comments 7.1, 4.2).

On a final note, we apologize for the sloppy grammar and spelling. We carefully addressed this issue in this new version of the manuscript.

Abstract & author summary

- line 44: make two sentences out of this long sentence

Author response: This has been amended

- line 47: delete 'allowing them to maintain…' this is repetition from above

Author response: This has been amended

- line 48: what do you mean with misunderstood? Not understood? Or are there really mistakes and / or misconceptions out there in the literature?

Author response: We were referring to the fact that it is not the focus of many studies and so it is still unclear. This has been amended to "have been overlooked"

- line 57: 'by the host': replace with 'final host choice' or something like that, because at the snail host level there is not always spatial isolation

Author response: this has been amended

- line 67-68: evolutionary biology?

<u>Author response:</u> Thank you for the suggestion this has been amended

- Line 68: replace ‘If’ by ‘While’

<u>Author response:</u> this has been amended

- Line 73: S. *haematobium* (species names always in full when first mentioning) and parasitize

<u>Author response:</u> this has been amended

- Line 70-74: sentence too long, split in two and rephrase ‘including out of endemic areas’

<u>Author response:</u> this has been amended

- Line 75: ‘…rather than having a homo-specific mate preference’

<u>Author response:</u> this has been amended

- Line 78: mechanisms

<u>Author response:</u> this has been amended

- Line 78: ‘but the one imposed by host specificity’? what do you mean? Please rephrase ‘except the one imposed by final host specificity’

<u>Author response:</u> this has been amended to “except those imposed by parasites species final host specificities”

- Line 81: ‘encounter each other’

<u>Author response:</u> this has been amended

Especially the author summary does not read very fluently, and there are quite some grammar mistakes.

<u>Author response:</u> apologies we hope that this new version will be suitable for publication

Introduction

- line 88= mechanism

<u>Author response:</u> this has been amended

- line 90: this sounds more like mate choice rather than behavioural isolation

<u>Author response:</u> we agree although we consider mate choice as behaviour. This has been amended

- line 91: individuals

<u>Author response:</u> this has been amended

- line 91: I would replace copulation by reproduction

<u>Author response:</u> this has been amended as required

- line 93: encounter

<u>Author response:</u> this has been amended according to reviewers #1 comment to "sperm and eggs mix but fertilization does not occur"

- line 96: less fertile **this has been amended and as suggested by reviewer #1 we included "non-viable"**

<u>Author response:</u> this has been amended

- line 96: hybrid lines

<u>Author response:</u> this has been amended

- line 98: making it difficult to predict

<u>Author response:</u> this has been amended

- line 104: terms

<u>Author response:</u> this has been amended

- line 105 and others: free-living

<u>Author response:</u> this has been amended

- line 108: compared to those of free-living…

Author response: this has been amended to “have received less attention than other free-living organisms regarding both hybridization and the role of reproductive isolation mechanisms”

- line 109: hostile rather than inimical?

Author response: this has been amended to “are dynamic habitats imposing”

- Line 112-113: rephrase this sentence, it is not really shaped through the host alone, it is shaped through the host – parasite interactions, and you first call this a strong isolation mechanism and a few words later you say ‘potentially’ preventing hybridization… the sounds less convinced. Also, you write ‘its hosts’, so plural, I would make the distinction already here between intermediate and final hosts, because in case of schistosomes sexual reproduction only takes place in the final host, so host choice at this level is more important than at the other level. You should discuss it at least somewhere, because in the abstract you only talk about ‘host’ choice.

Author response: thank you this has been amended to ease the understanding of the sentence

- Line 113: you mean ‘closely related’ species? Because all species are related somehow…

Author response: yes this has been amended thank you

- Line 116: plasmodium species (or you provide the genus names for the other two parasites you add here)

Author response: this has been amended

- Line 123: rephrase ‘schistosomiasis debilitating diseases’, this is not an official term (also, the disease is of great concern, rather than the parasites themselves I would say)

Author response: this has been amended to “Schistosomes are parasitic agents that cause schistosomiasis, a debilitating disease affecting [...]”

- Line 128: rephrase ‘among other trematodes’, you want to say here that they are an exception within the Trematoda

Author response: **this has been amended to “a feature not observed in other trematodes that are hermaphroditic”**

- Line 133: ‘this can lead to hybridisation’

Author response: **this has been amended**

- Line 136: influence rather than interest

Author response: **this has been amended**

- Line 136: First,

Author response: **this has been amended**

- Line 138: same host individual and schistosome species

Author response: **this has been amended for host individual. However, even though *Schistosoma* species could constitute a pre-zygotic isolation mechanism we would not include it as habitat barrier.**

- Line 144: female’s

Author response: **this has been amended**

- Line 145: of a sexually …

Author response: **this has been amended**

- Line 145: what does not depend upon species-specific pairing? Discuss this in a separate sentence in order to avoid too long sentences

Author response: **this has been amended. Our main idea was to precise that female’s maturation can be induced and maintained in the presence of males even if of another species**

- Line 147: stimulate

<u>Author response:</u> this has been amended

- Line 149: male worms' physiology or the physiology of male worms

<u>Author response:</u> this has been amended to "the physiology of male worms"

- Line 153: groups instead of clades?

<u>Author response:</u> this has been amended

- Line 155: schistosome

<u>Author response:</u> this has been amended

- Line 157: others, you mean other combinations?

<u>Author response:</u> in some species combination, there seems to by a mate recognition system that acts to avoid then from paring. They prefer their conspecifics in comparison to other species that may randomly mate. We rephrased in order to clarify this.

- Line 158: S. mattheei

<u>Author response:</u> this has been amended

- Line 156-159: in all these cases it should be mentioned that the viability of these crosses depend on the type of crosses, which parental species provides the male and which the female in the hybrid cross. Also, the way you write this you suggest that the first group of species can readily pair because there is less divergence between them (because this is what you write in the preceding sentence), while others are more selective because they are more divergent… but the divergence between S. *haematobium* and S. intercalatum is similar to the divergence between S. *haematobium* and S. mattheei. Also how can a combination be more selective or readily pair? It is the species that forms this combination that can be selective I would say. The grammar is quite sloppy in many cases, please take care of this.

<u>Author response:</u> Thank you or this feedback. We have amended this paragraph in order to add some element about the directionality of the cross (Comment 11) and followed your advice. Apologies for the sloppy grammar, we hope the revised version of

the manuscript is now suitable for publication.

- Line 174: non-human; also Cetartiodactyla is a superorder, and a superorder or a genus cannot be infected by parasites, but their members can

<u>Author response:</u> this has been amended

- Line 175: ruminants

<u>Author response:</u> this has been amended

- Line 177: rodents

<u>Author response:</u> this has been amended

- Line 191: outperforms the fitness of parental species

<u>Author response:</u> this has been amended

- Line 194: the sentence starting with 'Relying on such an integrative…' is redundant as it is mainly repetition

<u>Author response:</u> this has been amended

Material and methods

- Line 214: in or with Bulinus

<u>Author response:</u> this has been amended

- Line 219: B. truncates

<u>Author response:</u> this has been amended

- Line 223: were performed

<u>Author response:</u> this paragraph has been for most part re-written to provide more details on experimental infections and this sentence is no longer in it.

- Line 242: versus, What did you refer to here ?

<u>Author response:</u> We meant that we counted and collected worms that remained single and worms that were paired. This has been amended to make it clearer.

- Line 244: each worm and its

<u>Author response:</u> this has been amended

- Figure 1: I am a bit confused why you use the same color red for S. *haematobium* and S. *bovis* females?

<u>Author response:</u> this has been amended with a new Figure 1 showing each sex and species with a different colour. We also added examples of adult worms homospecific and heterospecific pairs

- Table 1: line 248: 'and' should not be in italic.

<u>Author response:</u> this has been amended

Line 250: the number of male and female S. *haematobium* and S. *bovis* worms

<u>Author response:</u> this has been amended

- Line 254: rephrase this sentence, grammatically incorrect

<u>Author response:</u> this has been amended

- Table 2: I think this table can be left out as everything is already explained in the text

<u>Author response:</u> Agreed and deleted from the manuscript. We kept the information relevant information in the text

- Line 278-279: parasite species names not in italic since the title is in italic

<u>Author response:</u> this has been amended

- Line 289: rephrase: as the volume of each reagent was halved

<u>Author response:</u> this has been amended

- Line 296: library construction

<u>Author response:</u> this has been amended

- Line 302: vs in full and not italic

<u>Author response:</u> this has been amended

- Line 307: all sample reads

<u>Author response:</u> this has been amended

- Line 318: each newly assembled gene or all newly assembled genes

<u>Author response:</u> this has been amended

- Line 319: were extracted from the S. *haematobium*

<u>Author response:</u> this has been amended

- Line 321: transcript

<u>Author response:</u> this has been amended

- Line 324: hetero-specific paired worms (or elsewhere you write hetero-specifically paired worms)

<u>Author response:</u> this has been amended

- Line 327: gene expression

<u>Author response:</u> this has been amended

- Line 338: sets of genes

<u>Author response:</u> this has been amended

Results

- line 349: experiment

<u>Author response:</u> this has been amended

- line 353: partners

<u>Author response:</u> this has been amended

- line 362: these are the risks of experimental research of course, and this cannot be avoided, but it does raise the question whether 5 replica's (5 hamsters) is enough to make robust

conclusions as in this particular case no reliable statistics can be done for S. *bovis* males' choice

<u>Author response:</u> **We agree, however the number of replicates are function of the number of paired worms that one is expected to recover from hamsters. From previous analyses we considered *cercariae* infectivity to be approximately 15% (15% of cercariae are expected to develop into adults). This means that for a minimum of 150 cercariae used for infestation, we expected recovering at least 23 paired worms per hamster x5 = 115 which is sufficient for robust statistical comparison. In the end, the infectivity for some combination was not as high as initially expected and this was exemplified by the mortality observed for hamsters. The downstream limitations are now included in the discussion (see also Comment 4.2) but as explained we believe that although strong conclusions cannot always be drawn for specific species and sex, our sample size was big enough to test our main hypothesis of random mating.**

- line 367: find instead of found

<u>Author response:</u> **this has been amended**

- Table 3, line 376: remaining single or that remained single

<u>Author response:</u> **this has been amended**

- Line 379: elsewhere you write P-value

<u>Author response:</u> **this has been homogenized throughout the text**

- Line 380: experiments

<u>Author response:</u> **this has been amended**

- line 385: male and female S. *haematobium*

<u>Author response:</u> **this has been amended**

- line 412: providing from?

<u>Author response:</u> this has been amended by rephrasing the sentence

- Line 426: does this 7% means 7% of all schistosome genes? Please specify this

<u>Author response:</u> 7% of the 18,648 unique genes of the reference transcriptome assembly. We have specified this in the text

- line 427: vs. in full as elsewhere

<u>Author response:</u> this has been amended

- line 462: GO terms

<u>Author response:</u> this has been amended

- line 463: male S. *haematobium*

<u>Author response:</u> this has been amended

- line 468: biological

<u>Author response:</u> this has been amended

- line 489: and not in italic

<u>Author response:</u> this has been amended

- line 500: correspond (in present tense)

<u>Author response:</u> this has been amended

- line 503: involved in ion

<u>Author response:</u> this has been amended

- line 506: functions

<u>Author response:</u> this has been amended

Discussion

- line 513: reproductive isolation mechanisms?

<u>Author response:</u> this has been amended

- Line 521: male and female

<u>Author response:</u> this has been amended

- Line 523: definitive host. This sentence is actually a final conclusion before starting the Discussion itself

<u>Author response:</u> this has been amended, we removed this sentence

- Line 532: S. mattheei

<u>Author response:</u> this has been amended

- Line 534: so this calls for more replica's in future experiments to verify this possibility!

<u>Author response:</u> we have added a sentence mentioning this

- Line 539: there are no barriers or there is no barrier

<u>Author response:</u> this has been amended

- Line 557: previous instead of precedent

<u>Author response:</u> this has been amended

- Line 558: there are no

<u>Author response:</u> this has been amended

- Line 560: what do you mean with 'male and female S. *haematobium* and S. *bovis*'? Between male S. *haematobium* and female S. *bovis*?

<u>Author response:</u> we were referring to all type of heterospecific pairings. This has been amended in the text to make it clearer

- Line 561: male S. *haematobium*

<u>Author response:</u> this has been amended

- Line 571: display a more

<u>Author response:</u> this has been amended

- Line 576: 'females species sexual maturation'?

Author response: **this has been amended**

- Line 587: female S. *bovis*

Author response: **this has been amended**

- Line 602: gonad-specific

Author response: **this has been amended**

- Line 579 – 612: this lengthy discussion is confusing and less convincing because right before this discussion you conclude that only few transcriptomic adjustments are associated with hetero-specific pairing and that the log2-Fold change in males were low, and that it seems difficult to conclude that one or the other sex is preferentially impacted, suggesting that your results are not so convincing. This is not so motivating for the reader then to follow this subsequent discussion

Author response: **We agree we have amended our discussion to improve this**

- Line 627-628: these observations could have indeed important consequences with respect to drug treatment, but again, how serious do we have to take this, and why would only S. *haematobium* males in heterospecific pairing have this response, and not S. *bovis* males?

Author response: **We agree and mentioned that our study does not allow to decipher whether the genes and biological process we identify are a particularity of *S. haematobium* or of our study only and that more work would be require in this direction.**

- Line 632: avenues

Author response: **this was amended**

- Line 634: but these report unidirectional introgression of a few *bovis* genes into the *haematobium* genome, so a dominance of *haematobium* genomic DNA, how can you reconcile or link this with your results? Wouldn't you expect more differences between the different crosses?

It has been proven in co-infection experiments that crosses are not always reciprocal, i.e. one cross performing better than the reverse cross. This could also be expected in these crosses as in Senegal many hybrids that were found appeared to have arisen of a female S. *bovis* x male S. *haematobium* pairing (although backcrossing in nature obscures these patterns, and in other areas, like Niger, the reverse crosses are more frequently found). I do not see any reference to this or to the topic of unidirectional hybridisation and unidirectional introgression, which I think is missing.

<u>Author response:</u> Indeed, most of the hybrids analysed to date show unidirectional introgression of *S. bovis* genes into the *haematobium* genome and this is a critical topic for which we have particular interest. However our data do not allow us to address this issue has we can see that both crosses are equally formed. This suggests that the asymmetry that is shown in the field and not everywhere is caused by a bias in selection of the hybrids' human host and urogenital tropism and/or result from post-zygotic barriers. We now discuss this topic more in depth (Comment 10.3, 10.4).

- line 648: I don't completely agree, because the different 'compartments' (strange term) do not prevent them from meeting each other in the liver, where mating takes place, only after that stage the couple moves through the hepatic portal vein to the egg-laying site. So there is plenty of opportunity in the liver, irrespective of the difference in tropism

<u>Author response:</u> we agree and thus removed this statement.

- line 653: how can these parasites be co-occurring (I would use the word sympatric) of their hosts are not? Please adapt

<u>Author response:</u> This has been amended.

- line 657: rephrase 'parental species individuals'

<u>Author response:</u> This has been amended.

- line 655-659: what do the authors want to say here exactly? Does ‘such hybrids’ refer to those hybrids resulting from an ancient hybridisation event? And do only ‘those hybrids’ present heterosis, in contrast to ‘other, more recent hybrids’? Please rephrase. Also, how do your results explain the fact that previous studies show that mainly ‘ancient hybrids’ are found in nature (Platt et al, 2019), while your experiments show that hybridisation is so ‘easy’? In line 668 you write that your result ‘echoes recent evidence of introgression’. I am not sure what you mean with this. Do you mean ‘evidence of recent introgression’ or ‘recent evidence of (ancient) introgression’? I would say the opposite, the high prevalence of hybrids is an echo or a reflection of the weak pre-zygotic barrier that you observed. But still, I don’t see how your results can be matched to the outcome of Platt et all, suggestion ancient hybridisation.

Author response: **We agree and have amended the discussion accordingly.**

- Line 669: a higher

Author response: **this has been amended**

- Line 670: if the hybrids always have higher fitness, why do you still see ‘pure S. *haematobium*’ and ‘pure’ S. *bovis* in places where they overlap? Wouldn’t the hybrids take over?

Author response: **This indeed a quite likely possibility. For instance, this has been demonstrated previously for *S. haematobium* and S. *intercalatum* hybrids in Cameroon that took over the parental species in less than 30 years (Tchuem Tchuenté et al. 2003).**

- Line 672: ‘new issues in the disease control’ and ‘alteration in the efficacy’ sound rather vague as a closing sentence, please be more specific.

Author response: **we have amended this sentence and changed it to “Understanding the modifications in the parasites life history traits, including their zoonotic potential and epidemiological outcomes are warranted if we are willing to control human and animal morbidity, reduce transmission and ultimately eliminate of schistosomiasis”**

Reviewer #3: Summary

The goal of this work appeared to be to test for evidence of species-specific mate choice between S. haemotobium and S. *bovis*, and also to see whether there are differentially expressed genes in adults engaged in homo vs. hetero-specific pairings. The found minimal evidence for mate choice and few strongly DE genes. In the discussion they speculate on possible roles of the few DE genes, but make no strong conclusions about any of them.

Comments

The authors did a mate choice experiment using cercariae of known sex in hamsters. They found no strong evidence that species-specific mate choice occurs by males or females of either species. The statistical analysis of the mate choice experiments seemed appropriate to me. If they have the data, it would be interesting if the authors could comment on the physical location of the hetero vs homo-specific pairings within the hamster (urogenital vs. mesenteric). I wondered whether different behavior might be observed if the definitive host was a larger mammal such as a bovine or human. Perhaps the authors would care to speculate or at least mention the possibility.

Author response: **The question of the physical location of the hetero vs homo-specific pairings within the host is very interesting. In experimentally infected hamsters it is known that "pure" *S haematobium* parasites are localized in the rectal part of the gut, while *S. bovis* parasite live in the mesenteric veins surrounding the small intestine. In our experiment we did not collected the information about the location of each couple whatever hetero- or homo-specifically paired since all the worms were recovered by portal perfusion. So far field studies conducted on *Mastomys* rodents tend to show that there is no specific localization of the worms and that both species can be found in the mesenteric**

vein (eg. Catalano et al. 2018). Moreover, the fact that infected humans emit hybrid parasites from both urine and feces (Huyse et al 2009) suggest that hetero-specific couple can be localized in either the veins surrounding the bladder or the gut. However, we agree that further studies especially on bigger host such as cattle or sheep would be worth conducted to tackle this question.

The authors also looked for evidence of differential gene expression in individuals of each sex and species when engaged in hetero vs. homo-specific pairings. They observed only a few dozen DE genes in females of either species. Oddly, they found zero DE genes in male S. *bovis*, but over 1000 in male S. haemotobium (although I wonder if figure 2a suggests the difference in S. haemotobium might be driven by one individual).

The almost complete lack of even minimally DE genes in S. *bovis* males shown in figure 3d is puzzling. I have never seen a volcano plot like this one. I would have expected more by chance alone with only n = 3 per treatment. Analysis of gene expression data is not my expertise, so I defer to other reviewers to comment on this. Or perhaps the authors could head off puzzled readers by explaining why this pattern obtains.

<u>**Author response:**</u> **The biological explanation for this is not straightforward. We now provide several hypotheses to explain such a result as detailed in the response to the comment 10.6. Moreover, we do not think that one individual among the male *S. haematobium* drove the number of DEGs identified since DESeq2 focus on DEG that have a similar profile between individuals (i.e., lower inter-individual variance). Hence if a particular response was only noticeable in one individual the DEGs would not necessary retained as significant (but might have a high log2 Fold Change). Conversely, as mentioned in the MS it is probably because the male *S. haematobium* displayed a homogeneous transcriptomic response with less interindividual variability that we**

identified a higher amount of DEG (Comment 10.6) but with overall low Log2 Fold Change.

Regarding the fact we did not identify any DEG for the comparison between homo- and hetero-specifically paired male *S. bovis* the results DESeq2, the volcano plot and the heatmap of sample distance seem to indicate congruent results: we cannot distinguish the hetero- and homo-specific worms based on their gene expression profile (Fig. 3, heatmap), we do have some DEG with relatively high Log2 Fold Change but they are not significant (Fig. 4, volcano plot) and this is confirmed by DESeq2 analysis (Supplementary File S1, sheet 5). We have nevertheless carefully reviewed these analyses and confirm our results.

Minor comments:

The manuscript could use some editing to fix various small grammatical errors and instances of odd English usage. Perhaps asking a native English speaker to read it through once for them would be helpful.

Author response: **Our apologies, we have paid particular attention into improving this.**

It would help Figure 3 if the authors would label, on the figure, which combination of sex and species is represented by each panel. Going back and forth between the legend and the figure is tedious for the reader.

Author response: **Thank you for the suggestion, we added this information directly in the figure.**

Figure 4. Is there some measure of statistical significance associated with the difference between blue and red bars that could be indicated on the figure?

Author response: **as mentioned in the material and methods section the statistical significance associated with the difference between blue and red bars were determined with a FDR threshold of 5%. We have included this in the figure caption.**

Measures of significance (p-values and FDR) associated with each biological process presented in this figure are given in the supplementary file S1 (Sheet 8). Since it would make the figure a bit messy if we report these measures at each bar, we included in the caption that the biological processes presented are significantly enriched at a FDR threshold of 5%.

Supplementary table S1 would be helped by species identifications.

Author response: **we have added the species identifications in the table**

PLOS authors have the option to publish the peer review history of their article (what does this mean?). If published, this will include your full peer review and any attached files.

If you choose "no", your identity will remain anonymous but your review may still be made public.

Do you want your identity to be public for this peer review? For information about this

choice, including consent withdrawal, please see our Privacy Policy.

Reviewer #1: No

Reviewer #2: No

Reviewer #3: No

Figure Files:

While revising your submission, please upload your figure files to the Preflight Analysis and Conversion Engine (PACE) digital diagnostic tool, https://pacev2.apexcovantage.com. PACE helps ensure that figures meet PLOS requirements. To use PACE, you must first register as a user. Then, login and navigate to the UPLOAD tab, where you will find detailed instructions on how to use the tool. If you encounter any issues or have any questions when using PACE, please email us at figures@plos.org.

Data Requirements:

Please note that, as a condition of publication, PLOS' data policy requires that you make available all data used to draw the conclusions outlined in your manuscript. Data must be deposited in an appropriate repository, included within the body of the manuscript, or uploaded as supporting information. This includes all numerical values that were used to generate graphs, histograms etc.. For an example see here:

http://www.plosbiology.org/article/info%3Adoi%2F10.1371%2Fjournal.pbio.1001908#s5.

Reproducibility:

To enhance the reproducibility of your results, PLOS recommends that you deposit laboratory protocols in protocols.io, where a protocol can be assigned its own identifier (DOI) such that it can be cited independently in the future. For instructions see https://journals.plos.org/plosntds/s/submission-guidelines#loc-methods

---

In compliance with data protection regulations, you may request that we remove your personal registration details at any time. (Use the following URL: https://www.editorialmanager.com/pntd/login.asp?a=r). Please contact the publication office if you have any questions.

Click here to access/download
Revised Article with Changes Highlighted
MS_resubmission_with_highlights.docx
