# Supplementary material for: No pre-zygotic isolation mechanisms between Schistosoma haematobium and Schistosoma bovis parasites: From mating interactions to differential gene expression
Source: PLoS Negl Trop Dis. 2021 May 4;15(5):e0009363. doi: 10.1371/journal.pntd.0009363 (PMC8127863; doi:10.1371/journal.pntd.0009363)
Supplement: Attachment — Submitted filename: 1_Letter_responses to reviewer comments_def.docx [file pntd.0009363.s004.docx]

**Dear Dr Lamberton,**

**Please find attached a significantly revised version of our manuscript PNTD-D-20-01594 taking into account the reviewers concerns.**

**We would like to thank you and anonymous reviewers for the detailed and thoughtful criticism on the manuscript. We have modified the manuscript according to reviewers’ suggestions. All changes are indicated in the manuscript and you will find below our responses after each point that were raised by the two reviewers. To ease the reading, the major reviewer’s comments have been numbered and reported as comments in the revised manuscript to locate the corrections using the “search” command.**

**We have substantially revised the title, authors** **summary, materiel and methods section as well as some result sections, discussion and conclusion to clarify and focus shortcomings. We believe this new version of the manuscript addresses the concerns of the reviewers and represents a substantially improved article.**

**The level of English and grammar within this paper has also been revised and we hope that you will find it suitable for publication in *Plos Neglected Tropical Disease.***

**We appreciate the time that you and anonymous reviewers have taken and would like to thank you for the constructive editorial process.**

**Sincerely,**

**Dr Mathieu-Bégné on behalf of all co-authors.**

Dear Mrs Mathieu-Bégné,
 
Thank you very much for submitting your manuscript "Pre-zygotic isolation mechanisms between *Schistosoma* *haematobium* and *Schistosoma* *bovis* parasites: from mating interactions to differential gene expression" for consideration at PLOS Neglected Tropical Diseases. As with all papers reviewed by the journal, your manuscript was reviewed by members of the editorial board and by several independent reviewers. In light of the reviews (below this email), we would like to invite the resubmission of a significantly-revised version that takes into account the reviewers' comments.

All three reviewers have commented on the level of English and grammar within this paper, that makes it hard to properly review this paper. I would recommend that in addition to all that scientific comments and specific language comments included that all authors really help rewrite this manuscript and if needed that you ask a native English speaker to help with the next version. I will send this out to reviewers again upon resubmission if the level of English has improved, but that really does need to be addressed.

We cannot make any decision about publication until we have seen the revised manuscript and your response to the reviewers' comments. Your revised manuscript is also likely to be sent to reviewers for further evaluation.

When you are ready to resubmit, please upload the following:
[1] A letter containing a detailed list of your responses to the review comments and a description of the changes you have made in the manuscript. Please note while forming your response, if your article is accepted, you may have the opportunity to make the peer review history publicly available. The record will include editor decision letters (with reviews) and your responses to reviewer comments. If eligible, we will contact you to opt in or out.
[2] Two versions of the revised manuscript: one with either highlights or tracked changes denoting where the text has been changed; the other a clean version (uploaded as the manuscript file).

Important additional instructions are given below your reviewer comments.
Please prepare and submit your revised manuscript within 60 days. If you anticipate any delay, please let us know the expected resubmission date by replying to this email. Please note that revised manuscripts received after the 60-day due date may require evaluation and peer review similar to newly submitted manuscripts.

Thank you again for your submission. We hope that our editorial process has been constructive so far, and we welcome your feedback at any time. Please don't hesitate to contact us if you have any questions or comments.

Sincerely,


Poppy Lamberton
Deputy Editor
PLOS Neglected Tropical Diseases

***********************

**Key Review Criteria Required for Acceptance?**
As you describe the new analyses required for acceptance, please consider the following:
**Methods**
-Are the objectives of the study clearly articulated with a clear testable hypothesis stated?
-Is the study design appropriate to address the stated objectives?
-Is the population clearly described and appropriate for the hypothesis being tested?
-Is the sample size sufficient to ensure adequate power to address the hypothesis being tested?
-Were correct statistical analysis used to support conclusions?
-Are there concerns about ethical or regulatory requirements being met?

Reviewer #1:

-Are the objectives of the study clearly articulated with a clear testable hypothesis stated?
Yes

**Comment 1**
-Is the study design appropriate to address the stated objectives?
Yes but more discussion is needed on the limitations of the study design

**Author response: We agree, and the discussion has been amended to include limitations of the study design. In particular we now discuss the impact of the low number of replicates (due to premature death of hamsters) on the conclusions drawn in the experiment 1 (Comment 1). Although we cannot conclude with certitude for this particular mate choice, this does not affect our general observation of random mate choice.**

**Comment 2**
-Is the population clearly described and appropriate for the hypothesis being tested?
More information is needed on the strains used and the details of the experiments

**Author response: Thank you for highlighting this unclear section; we have amended the text to help readers in understanding the experimental procedure and strains used. We added more information regarding the strains in the material and method section of our revised version of the manuscript (Comment 2.1). Concerning details of the experiments, we have now divided the protocol in three sections (“Experimental infections”, “Mate choice analysis” and “Forced reciprocal pairings”) that bring in more details and clarify (Comment 2.2, Comment 2.3 and Comment 2.4 respectively) common experimental procedure and how each objective was addressed (Comment 2.4).**

**We have amended Figure 1 to include an example of both homo and hetero-specific pairs where each species and sexes are represented with a different colour. In addition, we propose a new figure (Fig 2) to allow a better understanding of the procedure for homo and hetero- forced pairings.**

-Is the sample size sufficient to ensure adequate power to address the hypothesis being tested?
Yes
-Were correct statistical analysis used to support conclusions?
I would like this checked by a statistician

-Are there concerns about ethical or regulatory requirements being met?
No

Reviewer #2:

**Comment 3:**

- The overall objective is clear but the hypotheses could be more clearly formulated in the introduction. For example, somewhere in the paper it was stated that the authors expected the opposite outcome (more transcriptomic changes in females than in males), so this hypothesis could have been included at the end of the introduction, instead of the vague kind of conclusion in line 194-197. Additional testable hypotheses can be formulated.

**Author response: Thank you very much for taking time to review our paper and for your thorough comments and feedback; they were very helpful. We now have more clearly formalized hypothesis and predictions at the end of the introduction (Comment 3).**

**We suggest that in line to the current knowledge in schistosomes – mating does not seem to be a major reproductive isolation mechanism since hybrids are frequent in the field and parental species readily pair in the lab. However mating choice are necessary not only to assess the frequency of the phenomenon but also if any dominance could explain biased patters observed in the field. Moreover, as females undergo strong developmental changes upon pairing, we mentioned that we first expected finding a stronger molecular response in comparison to males.**

**Comment 4**:
- The study design is appropriate although some issues arise regarding statistical power. The results discussed in line 362 (premature death of two hamsters) does raise the question whether 5 replica’s (5 hamsters) is enough to make robust conclusions as in this particular case no reliable statistics can be done for S. *bovis* males’ choice.

**Author response: We do agree that we cannot make any robust conclusions for the result of the Experiment 3 due to the premature death of two hamsters and thus the poor statistical power (see also response to Comment 1).**

**This was explicitly stated in the results section (and now including in the Table 3, Comment 4.1) but also now stated in discussion section. We therefore suggest that this combination has to be tested in the future, but, although this hampers our ability to infer** ***S. bovis*** **males’ choice, our overall results strongly indicate that mating occur in a random fashion between the two species whatever the direction of the cross (Comment 4.2).**

- No concerns about ethical issues.
For more comments on the Methods section see General Comments.

**Author response: We have taken particular attention in the comments made in the General Comments and have amended the manuscript accordingly. Thank you for the helpful thoughts and the time spent at editing our manuscript.**

Reviewer #3:

Methods seem appropriate
--------------------

**Results**
-Does the analysis presented match the analysis plan?
-Are the results clearly and completely presented?
-Are the figures (Tables, Images) of sufficient quality for clarity?

Reviewer #1:

-Does the analysis presented match the analysis plan?
Yes

**Comment 5:**
-Are the results clearly and completely presented?
Improvement is needed for clarity of the results to allow interpretation

**Author response: We added some substantial changes to the manuscript to address this issue, notably by amending the Table 2 and the main text of the result section (e.g., Comment 5.1 for clarification of Table 2 regarding crosses made).**

-Are the figures (Tables, Images) of sufficient quality for clarity?
More information and clarity is needed as detailed in the attached document
**Author response: This has been addressed as proposed in the attached document, which we think, helped us a lot to improve the manuscript.** **To improve the clarity of the tables, we added information on each paired and single worm sex, hence allowing the readers to better identify tested combinations (Comment 5.1). Information was also included in the captions. For instance regarding the expected number of worms under random mating hypothesis, an example was given in material and method section and is now referred to in Table 2 caption (Comment 5.2). A new figure 2 has also been added to clarify the experimental mating procedure (see also response to Comment 2).**

Reviewer #2:

The analysis is sound and the results are well presented.

**Comment 6:**

Table 2 is redundant I would say.

**Author response: Thank you for your feedback. We agree with your comment.**

**Former Table 2 has been removed (Comment 6.1) and all relevant information for readers are now presented in the main text (Comment 6.2).**

For more comments on the Results section see General Comments.
**Author response: Thank you again. We have taken note of the General Comments section and have amended the manuscript accordingly.**

Reviewer #3: Analysis matches described goals of the work.
REsults are clearly presented.
Figures are sufficient quality

--------------------

**Conclusions**
-Are the conclusions supported by the data presented?
-Are the limitations of analysis clearly described?
-Do the authors discuss how these data can be helpful to advance our understanding of the topic under study?
-Is public health relevance addressed?


Reviewer #1:

-Are the conclusions supported by the data presented?
Yes

**Comment 7**
-Are the limitations of analysis clearly described?
No

**Author response: We agree. We have paid particular attention to bring in the limitations of our work. We did so in the discussion section, for mate choice experiments (Comment 7.1), for the interpretation of biological processes revealed by the transcriptomic analysis (Comment 7.2) and regarding the need to investigate the post-zygotic isolation mechanisms to better understand the hetero-specific pairing patterns in the field (Comment 7.3) (see also response to Comment 1).**

-Do the authors discuss how these data can be helpful to advance our understanding of the topic under study?
Ye
-Is public health relevance addressed?
Yes

Further comments on the discussion are available in the attached
**Author response: We have taken in consideration all comments and have amended the manuscript accordingly.**

Reviewer #2:

**Comment 8**

As put in my General comments, the Discussion needs reworking and should be more substantiated. The conclusions are not always clear or strong, I miss more references to similar studies, but also a proper discussion on the limitations and recommendations for future research are missing. The public health relevance is not really thoroughly discussed.
**Author response: Thank you for these constructive comments.**

**The discussion as been substantially modified and we have brought in information on similar studies and limitations (Comment 8.1, Comment 8.2, and Comment 8.3).**

**We have recommended several times throughout the manuscript that further studies would be needed when our experiments did not allow a strong conclusion (Comment 7.1 and 7.2). Concerning the public health relevance, we now explain better the concerns that our findings bring in terms of potential hybridization frequency (Comment 8.4) and the consequences on the parasites virulence and hybrids vigour (Comment 8.5)**

Reviewer #3:

Conclusions are supported by the data. They speculate quite a bit in the discussion about the potential importance of various DE genes. But this type of speculation is rampant in gene expression papers and they don't make any hard conclusions.

**Author response: Thank you very much for taking time to review our paper and for thoughtful criticism. We agree with the speculations inherent to gene expression analysis and have nuanced our conclusions and discussed our hypothesis in respect to the literature (cf Comment 7.2).**

--------------------

**Editorial and Data Presentation Modifications?**

Use this section for editorial suggestions as well as relatively minor modifications of existing data that would enhance clarity. If the only modifications needed are minor and/or editorial, you may wish to recommend “Minor Revision” or “Accept”.

Reviewer #1: The main modifications are needed in the clarity of the data and how it is presented. Comments are in the attached document.

**Author response: We are very grateful for your feedback and have taken particular attention to modify the manuscript accordingly. Thank you.**

**In particular, regarding some major comments we found in the attached document you will find our detailed answer below in order to explain some changes we made:**

1) **Overall, we re-structured the Material and Method section following your recommendation to clarify following the structure used in Result section**
2) **More detail has been given on the experimental procedure.**
3) **In order to gain clarity, we amended table content and table caption. Please note that it is normal that there is no total sum in the Table 2 (former Table 3) since Chi-square test involved comparison of expected number of pairs under random mating hypothesis to observed one at the level of the host (row) but the statistic of the test and its significance is computed considering all the hosts of a particular experiment. Similarly even if expected number of hetero-specific pairs could seem high some time compared to observed one, the aim of the test is to compare homo- and hetero-specific pairs number as expected if worms were mating randomly and considering the initial number of worms provided.**
4) **We considered your comment regarding the fact that we could not distinguished between mate choice and competition all along the manuscript.**
5) **A more extensive discussion about the asymmetry between some *Schistosoma* crosses as observed in the field is now provided in a dedicated paragraph of the discussion.**

Reviewer #2: (No Response)

Reviewer #3: (No Response)

--------------------

**Summary and General Comments**

Use this section to provide overall comments, discuss strengths/weaknesses of the study, novelty, significance, general execution and scholarship. You may also include additional comments for the author, including concerns about dual publication, research ethics, or publication ethics. If requesting major revision, please articulate the new experiments that are needed.

Reviewer #1:

**Comment 9**

This is an important and interesting study that shows the lack of pre zygotic isolation between S. *haematobium* and S. *bovis* supporting inter-species hyrbidisation an important scenario for human and animal health in Africa. Although, I consider the study of value and there is a substantial body of high quality of work, which is not easy to do (particularly the generation of the isolates and the mating experiments) the paper needed considerable improvements before it can be reviewed further. The authors should consider that the readers will not be familiar with these types of experiments and there is a need to add the detail that allows the readers to understand the experimental procedures and the data produced. There are also english and gramma errors many of which can be improved with careful reading. In the attached these are highlighted in the text showing the errors and where changes are needed. I have tried to cover the whole document but due to time some places may have been missed and careful reading and revision is needed. There are also comments on the attached to highlight where more clarity is needed and also where further information or discuss is warranted. The discussion is also very long and could be condensed by not repeating what is in the results.
**Author response:**

**Thank you for all the very useful comments and the precious time you have spent on this.**

**We think we have considerably improved the paper, by amending the parts of the manuscript that needed clarification and by providing further information so that readers can better understand the experimental procedures and the data produced. For that we have added details on the experiments (e.g., Comment 9.1, but most of the Material and Method section has been re-written and re-organized, cf Comment 2).**

**We also amended Figure 1 and added a new figure presenting the experiments for the transcriptomic analysis (Figure 2, Comment 9.2).**

**The English and grammar errors have been carefully revised.**

**Finally, we have reduced some parts of the discussion that were redundant with the results (Comment 9.3-9.6) or condensed some very long parts to be more straightforward (Comment 9.7-9.9). However, regarding the interest and thorough comments of the second reviewer we have also added supplementary discussion on the asymmetry in the direction of hybridization and introgression between schistosomes.**

Reviewer #2:

**Comment 10:**

This is a very interesting study, with interesting results. Experimental infections in hamsters show that there are no pre-zygotic barriers to mating between the human Schistosoma *haematobium* and the animal S. *bovis* parasite, neither are there any major transcriptomic responses following hetero-specific pairings. Even though a previous study by Webster and colleagues already showed that S. *bovis* and S. *haematobium* readily paired in laboratory hamsters (to which the present authors not refer, which I think is an omission), this is the first time that any transcriptomic study is done on hybrid crosses.

I was frustrated by the sloppy grammar and spelling throughout the entire manuscript, which gave the impression that the authors were in a hurry to submit this manuscript or they didn’t really care about this.

I also lack a discussion on the asymmetry in the direction of hybridisation and introgression between schistosomes, which is sometimes even unidirectional, but nothing is mentioned on this.

Also, it is repeatedly said that isolation ‘by the host’ is apparently the only barrier to hybridisation between these two schistosome species, but it is never specified which host they mean with that, the final or the intermediate one. Since mate choice and reproduction takes place in the final host, this one is of particular interest of course, but if the different intermediate hosts are not sympatric, then hybridisation will also be less frequent (if they final host does not move around too much). For S. *bovis* and S. *haematobium* this is more complicated, as intermediate host specificity of the latter varies with geography, but still this should be properly discussed as it could also explain why we see such regional differences in the distribution of hybrids.

Then finally, I also have some problems with parts of the discussion: some questions remain unanswered (e.g. why would only S. *haematobium* males in heterospecific pairing have this transcriptomic response?), the statistical power, and with the fact that I miss the broader picture, the reference to all the previous work on schistosome mating experiments. So therefore I think that these concerns should be addressed first before it can be accepted for publication.

**Author response: Thank you for all these very useful comments. We have included the reference to Webster and colleagues’ paper that showed that *S. bovis* and *S. haematobium* readily pair in laboratory hamster, which support our predictions (Comment 10.1). More generally, we amended the manuscript with more extensive references to previous works on schistosome mating experiments, which is most noticeable in the discussion section (e.g. Comment 10.2). Following your advice, we have added some discussion about the potential asymmetry in the direction of hybridization and introgression between schistosomes. We now discuss that although our data suggest that hybridization may be frequent in the field, recent genomic evidence suggest that introgressed hybrids may be ancient and that there is a strong bias in the hybrids profile (that also depend on the foci and the dynamics that are in place) (Comment 10.3). We then propose that post-zygotic mechanisms are the most likely to be shaping the outcome of hybridization (Comment 10.4).**

**We agree with you and acknowledge that there is indeed a lot to discuss about. However, in response to other comments about the length of the discussion we have tried to reach a middle ground by keeping the focus here on the pre-zygotic isolation mechanisms. We have specified wherever required that the final host is the one responsible for species reproductive isolation as it is only in that host that sexual reproduction occurs (Comment 10.5.0-10.5.2). Even though the idea of intermediate host specificity is highly relevant and important, we have hence preferred to keep on pre-zygotic barrier in the “site” in which species can interact. Moreover, as you underlie the situation with Sh and Sb is a bit more complex, both species can use the same mollusc (*Bulinus*) and it is difficult to predict the host-parasite compatibility outcome. This could be the entire focus of a dedicated study.**

**Regarding the biological explanation on why only male S*. haematobium* displayed more DEGs, we acknowledge that this is not straightforward and that we can only speculate. We thus re-wrote the paragraph dedicated to this topic in discussion section (Comment 10.6) in order to provide potential explanations: This particular response of male *S. haematobium* is most probably due to technical bias (replicate variability); the amount of DEGs seems high but does not imply a strong transcriptomic response compared to females worms since the log2Fold Change are low and/or this molecular plasticity in expression of genes is a mechanism by which *S. haematobium* males manage to be more competitive (compared to females from both species and *S. bovis* males) in hetero-specific pairing.**

**Regarding the mate choice experiment and in response to other comments we also now discuss more directly the issue raised by the statistical power in our study (Comments 7.1, 4.2).**

**On a final note, we apologize for the sloppy grammar and spelling. We carefully addressed this issue in this new version of the manuscript.**

Abstract & author summary

- line 44: make two sentences out of this long sentence

**Author response: This has been amended**
- line 47: delete ‘allowing them to maintain…’ this is repetition from above

**Author response: This has been amended**
- line 48: what do you mean with misunderstood? Not understood? Or are there really mistakes and / or misconceptions out there in the literature?

**Author response: We were referring to the fact that it is not the focus of many studies and so it is still unclear. This has been amended to “have been overlooked”**
- line 57: ‘by the host’: replace with ‘final host choice’ or something like that, because at the snail host level there is not always spatial isolation

**Author response: this has been amended**
- line 67-68: evolutionary biology?

**Author response: Thank you for the suggestion this has been amended**
- Line 68: replace ‘If’ by ‘While’

**Author response: this has been amended**
- Line 73: S. *haematobium* (species names always in full when first mentioning) and parasitize

**Author response: this has been amended**
- Line 70-74: sentence too long, split in two and rephrase ‘including out of endemic areas’

**Author response: this has been amended**
- Line 75: ‘…rather than having a homo-specific mate preference’

**Author response: this has been amended**
- Line 78: mechanisms

**Author response: this has been amended**
- Line 78: ‘but the one imposed by host specificity’? what do you mean? Please rephrase ‘except the one imposed by final host specificity’

**Author response: this has been amended to “except those imposed by parasites species final host specificities”**
- Line 81: ‘encounter each other’

**Author response: this has been amended**

Especially the author summary does not read very fluently, and there are quite some grammar mistakes.

**Author response: apologies we hope that this new version will be suitable for publication**

Introduction
- line 88= mechanism

**Author response: this has been amended**
- line 90: this sounds more like mate choice rather than behavioural isolation

**Author response: we agree although we consider mate choice as behaviour. This has been amended**
- line 91: individuals

**Author response: this has been amended**
- line 91: I would replace copulation by reproduction

**Author response: this has been amended as required**
- line 93: encounter

**Author response: this has been amended according to reviewers #1 comment to “sperm and eggs mix but fertilization does not occur”**
- line 96: less fertile **this has been amended and as suggested by reviewer #1 we included “non-viable”**

**Author response: this has been amended**
- line 96: hybrid lines

**Author response: this has been amended**
- line 98: making it difficult to predict

**Author response: this has been amended**
- line 104: terms

**Author response: this has been amended**
- line 105 and others: free-living

**Author response: this has been amended**
- line 108: compared to those of free-living…

**Author response: this has been amended to “have received less attention than other free-living organisms regarding both hybridization and the role of reproductive isolation mechanisms”**
- line 109: hostile rather than inimical?

**Author response: this has been amended to “are dynamic habitats imposing”**
- Line 112-113: rephrase this sentence, it is not really shaped through the host alone, it is shaped through the host – parasite interactions, and you first call this a strong isolation mechanism and a few words later you say ‘potentially’ preventing hybridization… the sounds less convinced. Also, you write ‘its hosts’, so plural, I would make the distinction already here between intermediate and final hosts, because in case of schistosomes sexual reproduction only takes place in the final host, so host choice at this level is more important than at the other level. You should discuss it at least somewhere, because in the abstract you only talk about ‘host’ choice.

**Author response: thank you this has been amended to ease the understanding of the sentence**
- Line 113: you mean ‘closely related’ species? Because all species are related somehow…

**Author response: yes this has been amended thank you**
- Line 116: plasmodium species (or you provide the genus names for the other two parasites you add here)

**Author response: this has been amended**
- Line 123: rephrase ‘schistosomiasis debilitating diseases’, this is not an official term (also, the disease is of great concern, rather than the parasites themselves I would say)

**Author response: this has been amended** **to “Schistosomes are parasitic agents that cause schistosomiasis, a debilitating disease affecting [...]”**
- Line 128: rephrase ‘among other trematodes’, you want to say here that they are an exception within the Trematoda

**Author response: this has been amended** **to** **“a feature not observed in other trematodes** **that are hermaphroditic”**
- Line 133: ‘this can lead to hybridisation’

**Author response: this has been amended**
- Line 136: influence rather than interest

**Author response: this has been amended**
- Line 136: First,

**Author response: this has been amended**
- Line 138: same host individual and schistosome species

**Author response: this has been amended for host individual. However, even though *Schistosoma* species could constitute a pre-zygotic isolation mechanism we would not include it as habitat barrier.**
- Line 144: female’s

**Author response: this has been amended**
- Line 145: of a sexually …

**Author response: this has been amended**
- Line 145: what does not depend upon species-specific pairing? Discuss this in a separate sentence in order to avoid too long sentences

**Author response: this has been amended. Our main idea was to precise that female’s maturation can be induced and maintained in the presence of males even if of another species**
- Line 147: stimulate

**Author response: this has been amended**
- Line 149: male worms’ physiology or the physiology of male worms

**Author response: this has been amended** **to “the physiology of male worms”**
- Line 153: groups instead of clades?

**Author response: this has been amended**
- Line 155: schistosome

**Author response: this has been amended**
- Line 157: others, you mean other combinations?

**Author response: in some species combination, there seems to by a mate recognition system that acts to avoid then from paring. They prefer their conspecifics in comparison to other species that may randomly mate. We rephrased in order to clarify this.**
- Line 158: S. mattheei

**Author response: this has been amended**
- Line 156-159: in all these cases it should be mentioned that the viability of these crosses depend on the type of crosses, which parental species provides the male and which the female in the hybrid cross. Also, the way you write this you suggest that the first group of species can readily pair because there is less divergence between them (because this is what you write in the preceding sentence), while others are more selective because they are more divergent… but the divergence between S. *haematobium* and S. intercalatum is similar to the divergence between S. *haematobium* and S. mattheei. Also how can a combination be more selective or readily pair? It is the species that forms this combination that can be selective I would say. The grammar is quite sloppy in many cases, please take care of this.

**Author response: Thank you or this feedback. We have amended this paragraph in order to add some element about the directionality of the cross (Comment 11) and followed your advice. Apologies for the sloppy grammar, we hope the revised version of the manuscript** **is now suitable for publication.**
- Line 174: non-human; also Cetartiodactyla is a superorder, and a superorder or a genus cannot be infected by parasites, but their members can

**Author response: this has been amended**
- Line 175: ruminants

**Author response: this has been amended**
- Line 177: rodents

**Author response: this has been amended**
- Line 191: outperforms the fitness of parental species

**Author response: this has been amended**
- Line 194: the sentence starting with ‘Relying on such an integrative…’ is redundant as it is mainly repetition

**Author response: this has been amended**


Material and methods

- Line 214: in or with Bulinus

**Author response: this has been amended**
- Line 219: B. truncates

**Author response: this has been amended**
- Line 223: were performed

**Author response: this paragraph has been for most part re-written to provide more details on experimental infections and this sentence is no longer in it.**
- Line 242: versus, What did you refer to here ?

**Author response: We meant that we counted and collected worms that remained single and worms that were paired. This has been amended to make it clearer.**
- Line 244: each worm and its

**Author response: this has been amended**
- Figure 1: I am a bit confused why you use the same color red for S. *haematobium* and S. *bovis* females?

**Author response: this has been amended with a new Figure 1 showing each sex and species with a different colour. We also added examples of adult worms homospecific and heterospecific pairs**

- Table 1: line 248: ‘and’ should not be in italic.

**Author response: this has been amended**

Line 250: the number of male and female S. *haematobium* and S. *bovis* worms

**Author response: this has been amended**
- Line 254: rephrase this sentence, grammatically incorrect

**Author response: this has been amended**
- Table 2: I think this table can be left out as everything is already explained in the text

**Author response: Agreed and deleted from the manuscript. We kept the information relevant information in the text**
- Line 278-279: parasite species names not in italic since the title is in italic

**Author response: this has been amended**
- Line 289: rephrase: as the volume of each reagent was halved

**Author response: this has been amended**
- Line 296: library construction

**Author response: this has been amended**
- Line 302: vs in full and not italic

**Author response: this has been amended**
- Line 307: all sample reads

**Author response: this has been amended**
- Line 318: each newly assembled gene or all newly assembled genes

**Author response: this has been amended**
- Line 319: were extracted from the S. *haematobium*

**Author response: this has been amended**
- Line 321: transcript

**Author response: this has been amended**
- Line 324: hetero-specific paired worms (or elsewhere you write hetero-specifically paired worms)

**Author response: this has been amended**
- Line 327: gene expression

**Author response: this has been amended**
- Line 338: sets of genes

**Author response: this has been amended**

Results

- line 349: experiment

**Author response: this has been amended**
- line 353: partners

**Author response: this has been amended**
- line 362: these are the risks of experimental research of course, and this cannot be avoided, but it does raise the question whether 5 replica’s (5 hamsters) is enough to make robust conclusions as in this particular case no reliable statistics can be done for S. *bovis* males’ choice

**Author response: We agree, however the number of replicates are function of the number of paired worms that one is expected to recover from hamsters. From previous analyses we considered *cercariae* infectivity to be approximately 15% (15% of cercariae are expected to develop into adults). This means that for a minimum of 150 cercariae used for infestation, we expected recovering at least 23 paired worms per hamster x5 = 115 which is sufficient for robust statistical comparison. In the end, the infectivity for some combination was not as high as initially expected and this was exemplified by the mortality observed for hamsters. The downstream limitations are now included in the discussion (see also Comment 4.2) but as explained we believe that although strong conclusions cannot always be drawn for specific species and sex, our sample size was big enough to test our main hypothesis of random mating.**

- line 367: find instead of found

**Author response: this has been amended**
- Table 3, line 376: remaining single or that remained single

**Author response: this has been amended**
- Line 379: elsewhere you write P-value

**Author response: this has been homogenized throughout the text**
- Line 380: experiments

**Author response: this has been amended**
- line 385: male and female S. *haematobium*

**Author response: this has been amended**
- line 412: providing from?

**Author response: this has been amended by rephrasing the sentence**
- Line 426: does this 7% means 7% of all schistosome genes? Please specify this

**Author response: 7% of the 18,648 unique genes of the reference transcriptome assembly. We have specified this in the text**
- line 427: vs. in full as elsewhere

**Author response: this has been amended**
- line 462: GO terms

**Author response: this has been amended**
- line 463: male S. *haematobium*

**Author response: this has been amended**
- line 468: biological

**Author response: this has been amended**
- line 489: and not in italic

**Author response: this has been amended**
- line 500: correspond (in present tense)

**Author response: this has been amended**
- line 503: involved in ion

**Author response: this has been amended**
- line 506: functions

**Author response: this has been amended**

Discussion

- line 513: reproductive isolation mechanisms?

**Author response: this has been amended**
- Line 521: male and female

**Author response: this has been amended**
- Line 523: definitive host. This sentence is actually a final conclusion before starting the Discussion itself

**Author response: this has been amended, we removed this sentence**
- Line 532: S. mattheei

**Author response: this has been amended**
- Line 534: so this calls for more replica’s in future experiments to verify this possibility!

**Author response: we have added a sentence mentioning this**
- Line 539: there are no barriers or there is no barrier

**Author response: this has been amended**
- Line 557: previous instead of precedent

**Author response: this has been amended**
- Line 558: there are no

**Author response: this has been amended**
- Line 560: what do you mean with ‘male and female S. *haematobium* and S. *bovis*’? Between male S. *haematobium* and female S. *bovis*?

**Author response: we were referring to all type of heterospecific pairings. This has been amended in the text to make it clearer**
- Line 561: male S. *haematobium*

**Author response: this has been amended**
- Line 571: display a more

**Author response: this has been amended**
- Line 576: ‘females species sexual maturation’?

**Author response: this has been amended**
- Line 587: female S. *bovis*

**Author response: this has been amended**
- Line 602: gonad-specific

**Author response: this has been amended**
- Line 579 – 612: this lengthy discussion is confusing and less convincing because right before this discussion you conclude that only few transcriptomic adjustments are associated with hetero-specific pairing and that the log2-Fold change in males were low, and that it seems difficult to conclude that one or the other sex is preferentially impacted, suggesting that your results are not so convincing. This is not so motivating for the reader then to follow this subsequent discussion

**Author response: We agree we have amended our discussion to improve this**
- Line 627-628: these observations could have indeed important consequences with respect to drug treatment, but again, how serious do we have to take this, and why would only S. *haematobium* males in heterospecific pairing have this response, and not S. *bovis* males?

**Author response: We agree and mentioned that our study does not allow to decipher whether the genes and biological process we identify are a particularity of *S. haematobium*** **or of our study only and that more work would be require in this direction.**
- Line 632: avenues

**Author response: this was amended**

- Line 634: but these report unidirectional introgression of a few *bovis* genes into the *haematobium* genome, so a dominance of *haematobium* genomic DNA, how can you reconcile or link this with your results? Wouldn’t you expect more differences between the different crosses?

It has been proven in co-infection experiments that crosses are not always reciprocal, i.e. one cross performing better than the reverse cross. This could also be expected in these crosses as in Senegal many hybrids that were found appeared to have arisen of a female S. *bovis* x male S. *haematobium* pairing (although backcrossing in nature obscures these patterns, and in other areas, like Niger, the reverse crosses are more frequently found). I do not see any reference to this or to the topic of unidirectional hybridisation and unidirectional introgression, which I think is missing.

**Author response: Indeed, most of the hybrids analysed to date show unidirectional introgression of *S. bovis* genes into the *haematobium* genome and this is a critical topic for which we have particular interest. However our data do not allow us to address this issue has we can see that both crosses are equally formed. This suggests that the asymmetry that is shown in the field and not everywhere is caused by a bias in selection of the hybrids’ human host and urogenital tropism and/or result from post-zygotic barriers. We now discuss this topic more in depth (Comment 10.3, 10.4).**

- line 648: I don’t completely agree, because the different ‘compartments’ (strange term) do not prevent them from meeting each other in the liver, where mating takes place, only after that stage the couple moves through the hepatic portal vein to the egg-laying site. So there is plenty of opportunity in the liver, irrespective of the difference in tropism

**Author response: we agree and thus removed this statement.**
- line 653: how can these parasites be co-occurring (I would use the word sympatric) of their hosts are not? Please adapt

**Author response: This has been amended.**
- line 657: rephrase ‘parental species individuals’

**Author response: This has been amended.**

- line 655-659: what do the authors want to say here exactly? Does ‘such hybrids’ refer to those hybrids resulting from an ancient hybridisation event? And do only ‘those hybrids’ present heterosis, in contrast to ‘other, more recent hybrids’? Please rephrase. Also, how do your results explain the fact that previous studies show that mainly ‘ancient hybrids’ are found in nature (Platt et al, 2019), while your experiments show that hybridisation is so ‘easy’? In line 668 you write that your result ‘echoes recent evidence of introgression’. I am not sure what you mean with this. Do you mean ‘evidence of recent introgression’ or ‘recent evidence of (ancient) introgression’? I would say the opposite, the high prevalence of hybrids is an echo or a reflection of the weak pre-zygotic barrier that you observed. But still, I don’t see how your results can be matched to the outcome of Platt et all, suggestion ancient hybridisation.

**Author response: We agree and have amended the discussion accordingly.**
- Line 669: a higher

**Author response: this has been amended**

- Line 670: if the hybrids always have higher fitness, why do you still see ‘pure S. *haematobium*’ and ‘pure’ S. *bovis* in places where they overlap? Wouldn’t the hybrids take over?

**Author response: This indeed a quite likely possibility. For instance, this has been demonstrated previously for *S. haematobium* and S*. intercalatum* hybrids in Cameroon that took over the parental species in less than 30 years (Tchuem Tchuenté et al. 2003).**

- Line 672: ‘new issues in the disease control’ and ‘alteration in the efficacy’ sound rather vague as a closing sentence, please be more specific.

**Author response: we have amended this sentence and changed it to “Understanding the modifications in the parasites life history traits, including their zoonotic potential and epidemiological outcomes are warranted if we are willing to control human and animal morbidity, reduce transmission and ultimately eliminate of schistosomiasis”**

Reviewer #3: Summary

The goal of this work appeared to be to test for evidence of species-specific mate choice between S. haemotobium and S. *bovis*, and also to see whether there are differentially expressed genes in adults engaged in homo vs. hetero-specific pairings. The found minimal evidence for mate choice and few strongly DE genes. In the discussion they speculate on possible roles of the few DE genes, but make no strong conclusions about any of them.

Comments
The authors did a mate choice experiment using cercariae of known sex in hamsters. They found no strong evidence that species-specific mate choice occurs by males or females of either species. The statistical analysis of the mate choice experiments seemed appropriate to me.
If they have the data, it would be interesting if the authors could comment on the physical location of the hetero vs homo-specific pairings within the hamster (urogenital vs. mesenteric). I wondered whether different behavior might be observed if the definitive host was a larger mammal such as a bovine or human. Perhaps the authors would care to speculate or at least mention the possibility.

**Author response: The question of the physical location of the hetero vs homo-specific pairings within the host is very interesting. In experimentally infected hamsters it is known that "pure" *S haematobium* parasites are localized in the rectal part of the gut, while *S. bovis* parasite live in the mesenteric veins surrounding the small intestine. In our experiment we did not collected the information about the location of each couple whatever hetero- or homo-specifically paired since all the worms were recovered by portal perfusion. So far field studies conducted on *Mastomys* rodents tend to show that there is no specific localization of the worms and that both species can be found in the mesenteric vein (eg. Catalano et al. 2018). Moreover, the fact that infected humans emit hybrid parasites from both urine and feces (Huyse et al 2009) suggest that hetero-specific couple can be localized in either the veins surrounding the bladder or the gut. However, we agree that further studies especially on bigger host such as cattle or sheep would be worth conducted to tackle this question.**

The authors also looked for evidence of differential gene expression in individuals of each sex and species when engaged in hetero vs. homo-specific pairings. They observed only a few dozen DE genes in females of either species. Oddly, they found zero DE genes in male S. *bovis*, but over 1000 in male S. haemotobium (although I wonder if figure 2a suggests the difference in S. haemotobium might be driven by one individual).

The almost complete lack of even minimally DE genes in S. *bovis* males shown in figure 3d is puzzling. I have never seen a volcano plot like this one. I would have expected more by chance alone with only n = 3 per treatment. Analysis of gene expression data is not my expertise, so I defer to other reviewers to comment on this. Or perhaps the authors could head off puzzled readers by explaining why this pattern obtains.

**Author response: The biological explanation for this is not straightforward*.* We now provide several hypotheses to explain such a result as detailed in the response to the comment 10.6. Moreover, we do not think that one individual among the male *S. haematobium* drove the number of DEGs identified since DESeq2 focus on DEG that have a similar profile between individuals (i.e., lower inter-individual variance). Hence if a particular response was only noticeable in one individual the DEGs would not necessary retained as significant (but might have a high log2 Fold Change). Conversely, as mentioned in the MS it is probably because the male *S. haematobium* displayed a homogeneous transcriptomic response with less interindividual variability that we identified a higher amount of DEG (Comment 10.6) but with overall low Log2 Fold Change.**

**Regarding the fact we did not identify any DEG for the comparison between homo- and hetero-specifically paired male *S. bovis* the results DESeq2, the volcano plot and the heatmap of sample distance seem to indicate congruent results: we cannot distinguish the hetero- and homo-specific worms based on their gene expression profile (Fig. 3, heatmap), we do have some DEG with relatively high Log2 Fold Change but they are not significant (Fig. 4, volcano plot) and this is confirmed by DESeq2 analysis (Supplementary File S1, sheet 5). We have nevertheless carefully reviewed these analyses and confirm our results.**

Minor comments:

The manuscript could use some editing to fix various small grammatical errors and instances of odd English usage. Perhaps asking a native English speaker to read it through once for them would be helpful.

**Author response: Our apologies, we have paid particular attention into improving this.**

It would help Figure 3 if the authors would label, on the figure, which combination of sex and species is represented by each panel. Going back and forth between the legend and the figure is tedious for the reader.

**Author response: Thank you for the suggestion, we added this information directly in the figure.**

Figure 4. Is there some measure of statistical significance associated with the difference between blue and red bars that could be indicated on the figure?

**Author response: as mentioned in the material and methods section the statistical significance associated with the difference between blue and red bars were determined with a FDR threshold of 5%. We have included this in the figure caption.**

**Measures of significance (p-values and FDR) associated with each biological process presented in this figure are given in the supplementary file S1 (Sheet 8). Since it would make the figure a bit messy if we report these measures at each bar, we included in the caption that the biological processes presented are significantly enriched at a FDR threshold of 5%.**

Supplementary table S1 would be helped by species identifications.

**Author response: we have added the species identifications in the table**

--------------------

PLOS authors have the option to publish the peer review history of their article ([what does this mean?](https://journals.plos.org/plosntds/s/editorial-and-peer-review-process#loc-peer-review-history)). If published, this will include your full peer review and any attached files.


If you choose “no”, your identity will remain anonymous but your review may still be made public.


**Do you want your identity to be public for this peer review?** For information about this choice, including consent withdrawal, please see our [Privacy Policy](https://www.plos.org/privacy-policy).

Reviewer #1: No

Reviewer #2: No

Reviewer #3: No


Figure Files:
While revising your submission, please upload your figure files to the Preflight Analysis and Conversion Engine (PACE) digital diagnostic tool, https://pacev2.apexcovantage.com. PACE helps ensure that figures meet PLOS requirements. To use PACE, you must first register as a user. Then, login and navigate to the UPLOAD tab, where you will find detailed instructions on how to use the tool. If you encounter any issues or have any questions when using PACE, please email us at figures@plos.org.
 
Data Requirements:
Please note that, as a condition of publication, PLOS' data policy requires that you make available all data used to draw the conclusions outlined in your manuscript. Data must be deposited in an appropriate repository, included within the body of the manuscript, or uploaded as supporting information. This includes all numerical values that were used to generate graphs, histograms etc.. For an example see here: http://www.plosbiology.org/article/info%3Adoi%2F10.1371%2Fjournal.pbio.1001908#s5.
 
Reproducibility:
To enhance the reproducibility of your results, PLOS recommends that you deposit laboratory protocols in protocols.io, where a protocol can be assigned its own identifier (DOI) such that it can be cited independently in the future. For instructions see https://journals.plos.org/plosntds/s/submission-guidelines#loc-methods

__________________________________________________
In compliance with data protection regulations, you may request that we remove your personal registration details at any time. (Use the following URL: https://www.editorialmanager.com/pntd/login.asp?a=r). Please contact the publication office if you have any questions.
