# Supplementary material for: No pre-zygotic isolation mechanisms between Schistosoma haematobium and Schistosoma bovis parasites: From mating interactions to differential gene expression
Source: PLoS Negl Trop Dis. 2021 May 4;15(5):e0009363. doi: 10.1371/journal.pntd.0009363 (PMC8127863; doi:10.1371/journal.pntd.0009363)
Supplement: Attachment — Submitted filename: response reviewers.docx [file pntd.0009363.s006.docx]

**Dear Dr Lamberton,**

**Please find attached our revised manuscript PNTD-D-20-01594 according to the review recommendations. You can find below a detailed list of our responses to all review comments. Any change in the manuscript are localized in the manuscript based on line numbers of the version with highlight.**

**We are grateful our manuscript is likely to be accepted for publication in *PLOS Neglected Tropical Disease* and would like to thank you and reviewers for the constructive editorial process.**

**Sincerely,**

**Dr Mathieu-Bégné for all co-authors**

Dear Mrs Mathieu-Bégné,

Thank you very much for submitting your manuscript "No pre-zygotic isolation mechanisms between Schistosoma haematobium and Schistosoma bovis parasites: from mating interactions to differential gene expression" for consideration at PLOS Neglected Tropical Diseases. As with all papers reviewed by the journal, your manuscript was reviewed by members of the editorial board and by several independent reviewers. The reviewers appreciated the attention to an important topic and we all agree that the manuscript is now greatly improved. Based on the reviews, we are likely to accept this manuscript for publication, providing that you modify the manuscript according to the review recommendations. I look forward to seeing an updated paper resubmitted very soon.

Please prepare and submit your revised manuscript within 30 days. If you anticipate any delay, please let us know the expected resubmission date by replying to this email.

When you are ready to resubmit, please upload the following:

[1] A letter containing a detailed list of your responses to all review comments, and a description of the changes you have made in the manuscript.

Please note while forming your response, if your article is accepted, you may have the opportunity to make the peer review history publicly available. The record will include editor decision letters (with reviews) and your responses to reviewer comments. If eligible, we will contact you to opt in or out

[2] Two versions of the revised manuscript: one with either highlights or tracked changes denoting where the text has been changed; the other a clean version (uploaded as the manuscript file).

Important additional instructions are given below your reviewer comments.

Thank you again for your submission to our journal. We hope that our editorial process has been constructive so far, and we welcome your feedback at any time. Please don't hesitate to contact us if you have any questions or comments.

Sincerely,

Poppy H L Lamberton

Deputy Editor

PLOS Neglected Tropical Diseases

Poppy Lamberton

Deputy Editor

PLOS Neglected Tropical Diseases

***********************

The manuscript is now greatly improved and I look forward to seeing these minor changes suggested by two of the reviewers amended and an updated paper resubmitted very soon.

Reviewer's Responses to Questions

Key Review Criteria Required for Acceptance?

As you describe the new analyses required for acceptance, please consider the following:

Methods

-Are the objectives of the study clearly articulated with a clear testable hypothesis stated?

-Is the study design appropriate to address the stated objectives?

-Is the population clearly described and appropriate for the hypothesis being tested?

-Is the sample size sufficient to ensure adequate power to address the hypothesis being tested?

-Were correct statistical analysis used to support conclusions?

-Are there concerns about ethical or regulatory requirements being met?

Reviewer #1: The methods are now clear and easy to follow

Reviewer #2: yes

Reviewer #3: Acceptable

--------------------

<b>Results</b>

-Does the analysis presented match the analysis plan?

-Are the results clearly and completely presented?

-Are the figures (Tables, Images) of sufficient quality for clarity?

Reviewer #1: The results are clearly presented.

Reviewer #2: yes

Reviewer #3: Acceptable

--------------------

Conclusions

-Are the conclusions supported by the data presented?

-Are the limitations of analysis clearly described?

-Do the authors discuss how these data can be helpful to advance our understanding of the topic under study?

-Is public health relevance addressed?

Reviewer #1: The discussion supported the data presented and is informative. The limitations are clearly presented and there relevance to public health is addressed.

Reviewer #2: yes

Reviewer #3: Acceptable

--------------------

Editorial and Data Presentation Modifications?

Use this section for editorial suggestions as well as relatively minor modifications of existing data that would enhance clarity. If the only modifications needed are minor and/or editorial, you may wish to recommend “Minor Revision” or “Accept”.

Reviewer #1: See general comments

Reviewer #2: The authors did a good job in addressing all the comments of the referees, they adapted the text and figures/tables where needed or suggested, and this resulted in a much-improved version of the manuscript. I think this version can be accepted for publication.

A few minor comments:

Line 43-44: before reproduction is also ‘during the life cycle’, so why not simply saying ‘These preventive barriers can act before reproduction, “pre-zygotic barriers”, or after reproduction/fertilisation, “post-zygotic barriers”?

🡺 **Thank you for the suggestion. The text has been amended accordingly (Line 49).**

Line 561; draw

🡺 **This has been amended accordingly (Line 582).**

Line 569: male transcriptomes

🡺 **This has been amended accordingly (Line 592).**

Line 640; to overcome their divergence only I would say, not to overcome their relatedness

**🡺 Agreed.** **This has been amended accordingly (Line 665).**

Line 654: a lower sensitivity to PZQ of the hybrid being at the origin of the spread was not at all proposed by Huyse et al., 2009 so please adapt.

🡺 **We removed the reference “Huyse et al., 2009” (Line 681).**

Line 708: S. mansoni [28]).

🡺 **This has been amended accordingly (Line 736).**

Line 711; I think the biased patterns in the field might indeed by due to post-zygotic isolation but not the rare encounter of first generation hybrids (or not only because of that). The latter is probably (also) due geographical variation in the level of sympatry between parental species, no?

**🡺 The idea here is that first generation hybrids may be produced on a relatively high frequency (indeed according to the geographical variation in the level of sympatry between species, for example, although not exclusive), but that many individuals could be rapidly counter-selected because of post-zygotique barriers (affecting their survival either in the definitive or intermediate hosts). Therefore, although hybridization could be frequent, only few first generation hybrids survive and this could be a cause of the relatively rare encounter in early generation hybrids (amongst other causes, indeed). Nevertheless, this remains an open question to address in the field. We have added “and also potentially the relatively rare encounter in early generation hybrids” to nuance this in the text (Line 739).**

Reviewer #3: They adequately addressed the issues of language usage.

--------------------

Summary and General Comments

Use this section to provide overall comments, discuss strengths/weaknesses of the study, novelty, significance, general execution and scholarship. You may also include additional comments for the author, including concerns about dual publication, research ethics, or publication ethics. If requesting major revision, please articulate the new experiments that are needed.

Reviewer #1: The paper is much improved, and the authors have taken the time and effort to amend the manuscript according to the reviewer’s comments. This is a highly informative paper that adds to our understand on the interactions between S. haematobium and S. bovis. It will also open several research avenues for future work.

**🡺 Thank you very much for your feedback.**

I have the following minor comments.

Gramma punctuation is needed in several places so a good go over will help

Some further modifications of the English is needed. Areas that I managed to pick up are highlighted.

Line 120 – there are 23 species not 25

🡺 **Thank you, we revised the manuscript in the highlighted regions. We also took note of the information on the number of species and amended the MS accordingly (Line 127-128).**

Line 121 – to be precise 20 infect animals if you include S. mansoni that is found in rodents and monkeys.

🡺 **Indeed, we amended the text accordingly (Line 128).**

Line 188-189 – make it clear that the heterosis has been observed in experimental infections not in the natural setting.

**🡺 Yes, we added “in experimental infections” to make it clear (Line 198).**

Line 220-222- the animal license information should be checked with the editors regarding how it should be written.

**🡺 Although this is the version provided by our institution for other publications we will refer to the editors.**

Line 229 – what do you mean by sympatric B. truncatus – do you mean local natural hosts?

🡺 **Yes, the host collected on the same transmission site - We changed sympatric to local (Line 241).**

Line 250 – add “below” to the end of the sentence.

🡺 **This has been amended (Line 264).**

Figure 2 – S. h = S. bovis needs correcting. Also, the symbols are both in the figure and legend. Only one is needed.

🡺 **We corrected the figure 2 and remove the symbols in the legend. (Line 319-321).**

Table 1: in Exp. 2 and 4 you say that this is a female choice experiment. Is this not more competition of the two male species?

🡺 **Because sexual interactions are hidden in a host it is very difficult to differentiate between the two classical driving forces of sexual selection (choice and/or competition). We propose to change “choice” by "limiting sex" to avoid making any assumption on the choice and/or competition (see table 1 line 270-271).**

Line 290 – make it clear that this section is moving onto the molecular work and not the analysis of pairings.

🡺 **We changed the title of this section to “Forced pairing and underlying molecular analysis” to precise that we are moving onto molecular analysis of pairing (Line 305).**

Line 296 – make it clear how the worms were separated. It is important here that there is not contamination between males and females, so the detail is needed.

**🡺 Indeed, with the female being in the male’s gynecophoral** **canal contamination could occur easily if no precaution is taken when separating the worms. Each pair was delicately separated under a binocular magnifier using a small paintbrush to avoid causing damages to the worms. This has been added to the text (Line 312-313).**

Line 327 – “effect” not needed

🡺 **We amended the text accordingly (Line 343).**

Line 328 – reference the Galaxy instance. E.g., is it a software or a machine?

🡺 **Galaxy is a web interface that provides a multitude of software. We reference Galaxy instance as suggested on Galaxy website.**

Table 2 column 6 and 7 remove the words or change homo specific or hetero specific as they are not paired. Thy can just be called unpaired worms. It would also be better to have the exp. row above the data rather and below.

For figure 4 is it important to distinguish between hetero and homo paired worms ?

**🡺 We amended the Table 2 and changed homo specific or hetero specific to “Single worms” (Line 412-418). Concerning Figure 4 is not important to distinguish between hetero and homo paired worms the genes expression profiles here correspond to that of hetero-specifically compared to homo-specifically paired worms*.* This is mentioned in the figure’s caption (Line 488).**

Line 481 – show what GO means,

🡺 **GO was defined line 377 (at its first occurrence).**

Line 553 – is there any evidence for female selecting their partners. They are very underdeveloped until they are paired?

🡺 **As we explained previously and in the MS, the pairing could result either from female choice and/or male competition. Some previous experimental work has shown that female worms could indeed select their partner and changes mate for more dissimilar males when new comers infect the same host (Beltran et al., 2008). This process seems to occur after females have reached their sexual maturity – but indeed females are very underdeveloped before then and initial mating could be in first instance attributed to males taking small females in their canal.**

Line 555 – this S. intercalatum strain is actually now named S. guineensis – maybe add a note on this and also where it is referenced in the intro.

**🡺 Indeed, until 2003 *S. guineensis* and *S. intercalatum* were considered as one species (i.e., *S. intercalatum*) and then split based on their mitochondrial divergence (Pagès et al., 2003, Webster et al., 2006). We added this precision in the introduction i.e., at the first occurrence of *S. intercalatum* (Line 163)and then added *S. guineensis* where *S. intercalatum* is cited (line 166, 171, 575, 735) .**

Line 565 – reference the study Webster et al., 2012 that also suggested that there was no mate choice

**🡺 Indeed, we added the information in the text (Line 585).**

Line 599 - suggest you add here that the majority of the hybrids found in the field appear to be a result of a cross between S. h male and S. b female based on cox1 and ITS sequencing. It is better to say this than state that they are as more work is needed to clarify that.

🡺 **We have slightly rephrased the sentence to make it clear (Line 621-623).**

Line 655 – I think it is important to point out that any changes in PZQ response by hybrids is very theoretical and there is not current evidence that there is any difference in drug response in natural infections.

🡺 **We have added a sentence in the text mentioning this (Line 681-684).**

Line 707- check if the S. intercalatum should be called S. guineensis. S. intercalatum is the Zaire/DRC strain. Cameroon and other areas is S. guineensis.

**🡺 Indeed, *S. intercalatum* is restricted to central Africa regions, namely the Democratic Republic of Congo and Zaire, whereas *S. guineensis* is found on the South West coastal countries. In the present study the strain was collected from Edea in Cameroun suggesting that it corresponds to *S. guineensis.* We added *S. guineensis* whenever it previously referred to *S. intercalatum* (Line 735).**

Reviewer #2: (No Response)

Reviewer #3: I believe the authors have adequately addressed my comments on their original submission. The main points of the paper, that there is random mating and few differentially expressed genes in interspecies pairings, are sufficiently supported and worth putting into the literature

**🡺 Thank you**

--------------------

PLOS authors have the option to publish the peer review history of their article (<a href=["https://journals.plos.org/plosntds/s/editorial-and-peer-review-process#loc-peer-review-history"](https://journals.plos.org/plosntds/s/editorial-and-peer-review-process#loc-peer-review-history) target="_blank">what does this mean?</a>). If published, this will include your full peer review and any attached files.<br><br>

If you choose “no”, your identity will remain anonymous but your review may still be made public.<br><br>

<b>Do you want your identity to be public for this peer review?</b> For information about this choice, including consent withdrawal, please see our <a href=["https://www.plos.org/privacy-policy"](https://www.plos.org/privacy-policy) target="_blank">Privacy Policy</a>.

Reviewer #1: Yes: Dr Bonnie Webster

Reviewer #2: No

Reviewer #3: No

Figure Files:

While revising your submission, please upload your figure files to the Preflight Analysis and Conversion Engine (PACE) digital diagnostic tool, <https://pacev2.apexcovantage.com>. PACE helps ensure that figures meet PLOS requirements. To use PACE, you must first register as a user. Then, login and navigate to the UPLOAD tab, where you will find detailed instructions on how to use the tool. If you encounter any issues or have any questions when using PACE, please email us at [figures@plos.org](mailto:figures@plos.org).

Data Requirements:

Please note that, as a condition of publication, PLOS' data policy requires that you make available all data used to draw the conclusions outlined in your manuscript. Data must be deposited in an appropriate repository, included within the body of the manuscript, or uploaded as supporting information. This includes all numerical values that were used to generate graphs, histograms etc.. For an example see here: <http://www.plosbiology.org/article/info%3Adoi%2F10.1371%2Fjournal.pbio.1001908#s5>.

Reproducibility:

To enhance the reproducibility of your results, PLOS recommends that you deposit laboratory protocols in protocols.io, where a protocol can be assigned its own identifier (DOI) such that it can be cited independently in the future. For instructions see <http://journals.plos.org/plosntds/s/submission-guidelines#loc-materials-and-methods>

__________________________________________________

In compliance with data protection regulations, you may request that we remove your personal registration details at any time. (Use the following URL: <https://www.editorialmanager.com/pntd/login.asp?a=r>). Please contact the publication office if you have any questions.
